# Gold(III), Mercury(II), and Palladium(II) Complexes of a Series of Isomeric Bis(mono- and dialkoxyphenyl)pyridines: Introduction of Gold through Transmetalation and Catalysis

**DOI:** 10.1021/acs.inorgchem.3c03791

**Published:** 2024-04-18

**Authors:** Alice Jane McEllin, Christopher A. Goult, Golam Mohiuddin, Liam J. Curtis, Theo F. N. Tanner, Adrian C. Whitwood, Jason M. Lynam, Duncan W. Bruce

**Affiliations:** † Department of Chemistry, University of York Heslington, YORK YO10 5DD, U.K.; ‡ Department of Chemistry, University of Science & Technology Meghalaya, Ri-Bhoi, Meghalaya 793101, India

## Abstract

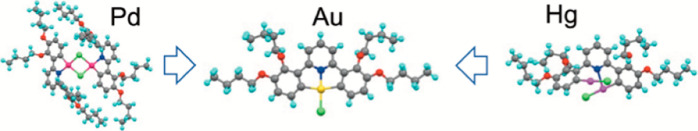

A series of isomeric bis-2,6-(monoalkoxyphenyl)pyridine and bis-2,6-(dialkoxyphenyl)pyridine ligands were synthesized and characterized. In order to prepare their chlorogold(III) complexes, intermediate chloromercury(II) complexes were first prepared, but unlike observations from previous studies where they were obtained impure and at best in moderate yield, here pure complexes were synthesized, many in rather high yields. Depending on the substitution pattern of the alkoxy chains on the ligands, mono- and/or dimercurated complexes were obtained, characterized by ^1^H, ^13^C{^1^H}, and ^199^Hg NMR spectroscopy as well as, in several cases, by X-ray crystallography. Factors that may explain this unusual reactivity are discussed. In most cases, transmetalation to the related chlorogold(III) complex proceeded smoothly, although lower yields were obtained when starting from doubly mercurated precursors. Prompted by the propensity of these ligands to mercurate, attempts were made to effect direct auration, but none was successful. However, dimeric, orthometalated complexes of palladium(II) could be prepared and were also amenable to transmetalation to the chlorogold(III) complex, providing for a mercury-free synthesis.

## Abstract 2

Varying the substitution pattern on a series of 2,6-bis(alkoxyphenyl)pyridine ligands allows high-yield syntheses and isolation and full characterization of a series of mercury(II) complexes that act as transmetalation precursors to analogous gold(III) complexes. Palladium complexes of these ligands can also be prepared and successfully transmetalated with gold(III), providing a synthetic route with much less harmful intermediates.

## Introduction

**Figure 1:**
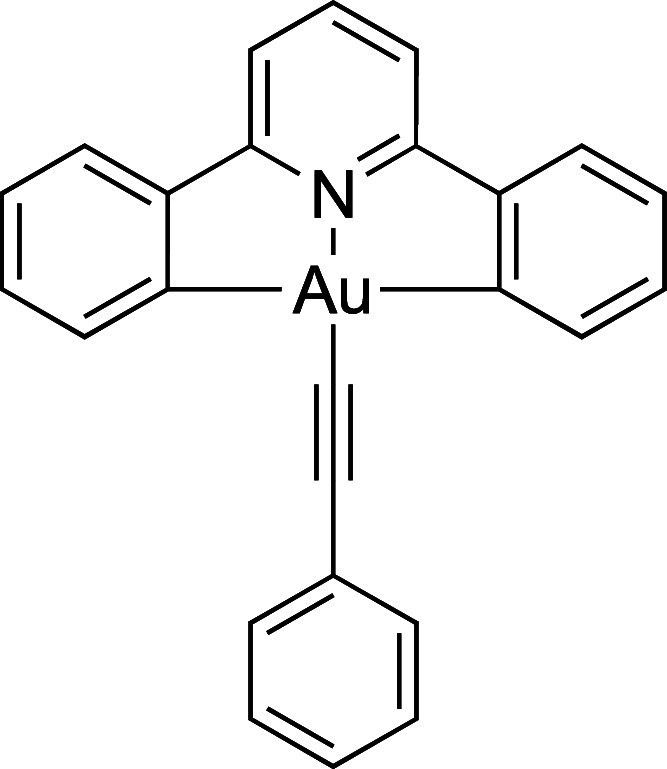
General structure of the emissive gold(III) phenylacetylide complexes.

When compared with gold(I), the chemistry of organogold(III) is arguably much less explored, but in particular, since the observation of phosphorescence in C,N,C-pincer complexes of gold(III) with acetylide coligands (Figure 1), there has been an upsurge in interest in its chemistry. Thus, the groups of Yam and of Che in particular have exploited the photophysics of complexes of this general type, demonstrating, for example, luminescent gels as well as developing their deployment as the emissive component in OLED devices.^1−9^

Our own contributions in this area have been to modify C,N,C-pincer ligands and attendant phenylacetylide coligands to confer liquid-crystal properties on them and we have reported on the preparation, mesomorphism, photophysics, and device behavior.^10,11^ In particular, one complex, which had hydrocarbon chains on the pincer ligand and semiperfluorocarbon chains on the phenylacetylide, showed rather remarkable liquid-crystal properties. Thus, a fluid nematic phase, assigned as columnar nematic (N_Col_), was found between two much more ordered phases, namely, a columnar hexagonal (Col_h_) phase to higher temperature and a columnar rectangular phase (Col_r_) to lower temperature. It is believed that the formation of the Col_r_ phase is driven by the incompatibility of the hydrocarbon and fluorocarbon chains, which is overcome thermally in the Col_h_ phase. As such, the intermediate N_Col_ phase is believed to arise due to the frustration as the incompatibility is overcome.^12,13^

However, one of the issues associated with the preparation of gold(III) complexes of this type is the difficulty in realizing direct C–H activation from a suitable proligand by gold. This has necessitated the preparation of mercury(II) intermediates which then readily undergo transmetalation to the desired gold product.

**Figure 2:**
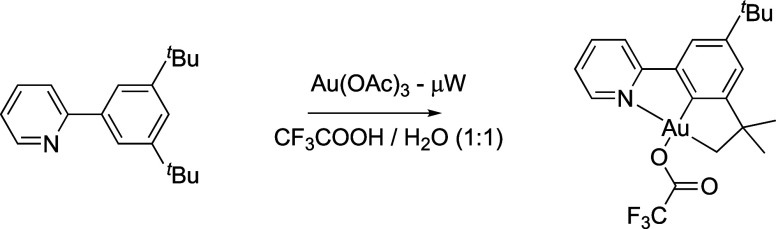
Formation of a C,C,N gold(III) chelate via activation of a ^*t*^Bu C–H bond of a 2-phenylpyridine.

There has, therefore, been some interest in direct auration reactions, and progress here has been realized quite effectively with 2-phenylpyridine ligands. In fact as long ago as 1989, Constable et al. found that by first preparing an η^1^-gold(III) complex bound through nitrogen, a 6-(thienyl)-2,2′-bipyridine will metalate to give the dichlorogold(III) product.^14^ Successful strategies reported more recently have used microwave irradiation, which has proved very effective with a wide range of substituted 2-phenylpyridines, including di-3,5-^*t*^butyl-2-phenylpyridine where the additional metalation of a ^*t*^Bu methyl group leads to a C,C,N chelating complex (Figure 2).^15−18^ It has also proved possible to undertake direct auration by a prefunctionalization of the substrate which, while giving good yields of the product, nonetheless requires additional synthetic chemistry by way of preparation of a diazonium salt or a lithiated reagent in order to obtain the precursor.^19−21^ In addition, there are various reports of the formation of Au(III) via oxidative addition to Au(I).^22−25^

However, perhaps the most significant new report has been that of Martín et al., who have used [Cp*RhCl_2_]_2_ to catalyze the direct auration of a series of 2-phenylpyridines in good yield as discussed in a little more detail below.^26^

Following on from our findings regarding the mesophase behavior of complexes of gold(III),^10−13^ we were keen to explore the possibility of binding the gold chromophore to other photoactive moieties with the thought of realizing photodyads. However, in considering binding another chromophore through the fourth coordination site on gold with the C,N,C pincer ligand attached already, it was believed that the two chains in the 4,4′-positions of the pincer might extend out and interfere sterically with a second chromophore. In order to avoid that possibility, the preparation of a series of isomeric di- and tetraalkoxy pincers with substitution patterns that keep the chains more remote from the gold center was undertaken to provide structural flexibility.

Once these ligands were obtained, their gold(III) complexes were to be obtained via a chloromercury(II) intermediate as previously described, but in the process of preparing the mercury complexes, it became apparent that it was possible to obtain them in high yields and with good purity. This is in stark contrast to our previous findings with 4,4′-dialkoxy and 3,3′,4,4′-tetraalkoxy ligands where the mercury intermediates were obtained impure and in low to moderate yield. We therefore undertook a more detailed study of these mercurations and the subsequent transmetalation to gold, which are now reported and discussed.

## Results

### Ligand Synthesis

**Scheme 1:**
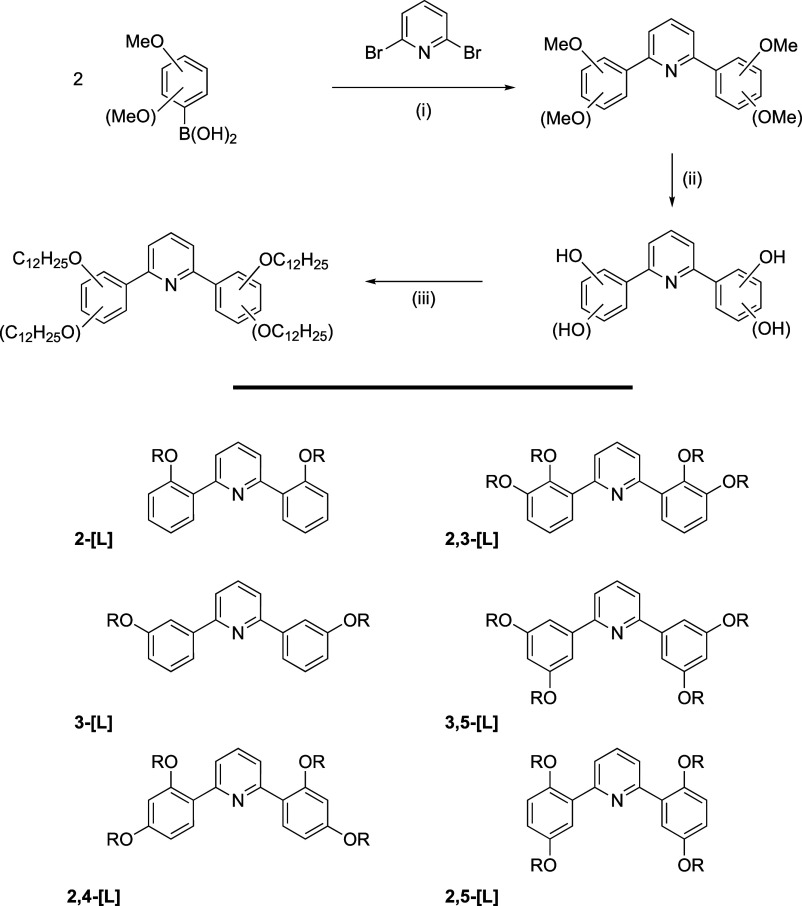
Top: Reaction Scheme for Synthesis of C,N,C Pincer Ligands: (i) [Pd_3_(OAc)_6_]/K_3_PO_4_/Glycol/80 °C/1.5 h; (ii) PyH^+^Cl^–^/200 °C; (iii) C_12_H_25_Br/K_2_CO_3_/Butanone/72 h. Bottom: Structures of Those Prepared in This Way with Labeling (R = C_12_H_25_)

The preparation of the ligands is as set out in Scheme 1 (which also includes the compound labeling used) and diphenylpyridines with 2,2′-, 2,2′,3,3′-, 2,2′,4,4′-, 2,2′,5,5′-, 3,3′-, and 3,3′,5,5′- substitution patterns were prepared. The scheme shows the preparation of ligands with C12 chains, although occasionally analogous materials with C4 chains were prepared in order to grow single crystals.

The ligands were prepared (Scheme 1) by first coupling the appropriate mono- or dimethoxyphenyl boronic acid with 2,6-dibromopyridine using a palladium catalyst (yields of around 90%) and then demethylating the resulting 2,6-di(methoxyphenyl)pyridines with pyridinium chloride (>90% yield). The final ligands were obtained by reaction with 1-bromododecane using standard *O*-alkylation conditions. The alkylation reactions proceeded smoothly except in the case of **2,4-[L]-OH** and **2,5-[L]-OH**, where the formation of an intramolecular hydrogen bond in the phenolic substrate meant that slightly more forcing conditions had to be used to obtain an acceptable yield. A small number of butyloxy homologues were also prepared in the same way as described later.

Single-crystal X-ray structures were obtained for the phenolic intermediate for **2,3-[L]**, and the methoxy intermediates for **3-[L]**, **4-[L]**, **2,3-[L]**, **2,4-[L]**, **2,5-[L]**, **3,4-[L]**, and **3,5-[L]**. The details are found in the Supporting Information (Figures S4–S12).

The ligands and their intermediates were characterized by NMR spectroscopy^a^ and it has been noted already that there is atropoisomerism in ligands where there is an alkoxy group in the 2,2′-positions on the phenyl rings.^27^ However, while the *anti*atropoisomer is chiral, it is not correct, as stated originally, that the complex signal arising from the coupling between the *p*-hydrogen and the *m*-hydrogens of the pyridine ring originates from a diastereotopic relationship between the *m*-hydrogens. Rather the spectrum arises as it is a second-order AB_2_ system (rather than an effectively first-order AX_2_ system, which would give rise to a simple triplet and doublet as observed in many of the ligands and complexes reported here).^28^ We have subsequently recorded the ^1^H NMR spectrum at 700 MHz and, while not reverting to a first-order AX_2_ pattern, it does come closer to a simple doublet and triplet (Figure S13). To evaluate the possibility that the atropoisomers of **2,3-[L]** might interchange thermally in solution, we conducted variable-temperature NMR experiments on both **2,3-[L]** and **2,3-[L]-OMe** over the temperature range 253 to 313 K. The spectra (Figures S14–S16) show no broadening of the peaks over this 60 K range (consistent with the absence of interchange), although as discussed in the Supporting Information, interestingly temperature-dependent ^1^H NMR chemical shifts were observed for both **2,3-[L]** and **2,3-[L]-OMe**.

### Synthesis of Mercury(II) Complexes

#### Warning

Mercury salts and organomercury complexes are toxic. Great care, as outlined in the Supporting Information, should be taken in preparing and handling materials of this type. Furthermore, while an extensive study is reported here, evidently, there are other confirmatory experiments that could have been carried out on some occasions. However, in order to reduce the handling of mercury, not all of these were attempted.

**Scheme 2:**
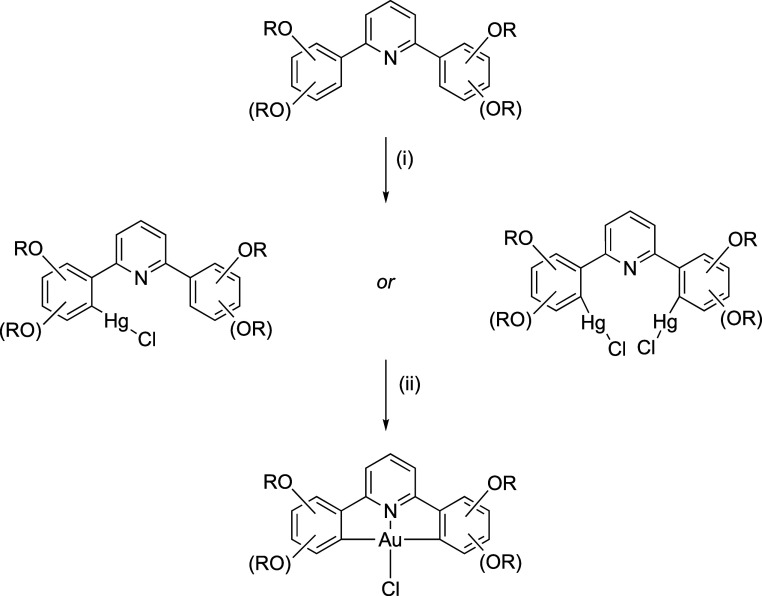
General Synthesis of the Mercury and Gold Complexes: (i) Hg(OAc)_2_/EtOH/Δ/LiCl; (ii) K[AuCl_4_]/MeCN(/CHCl_3_)/Δ; (R = C_12_H_25_)

**Figure 3:**
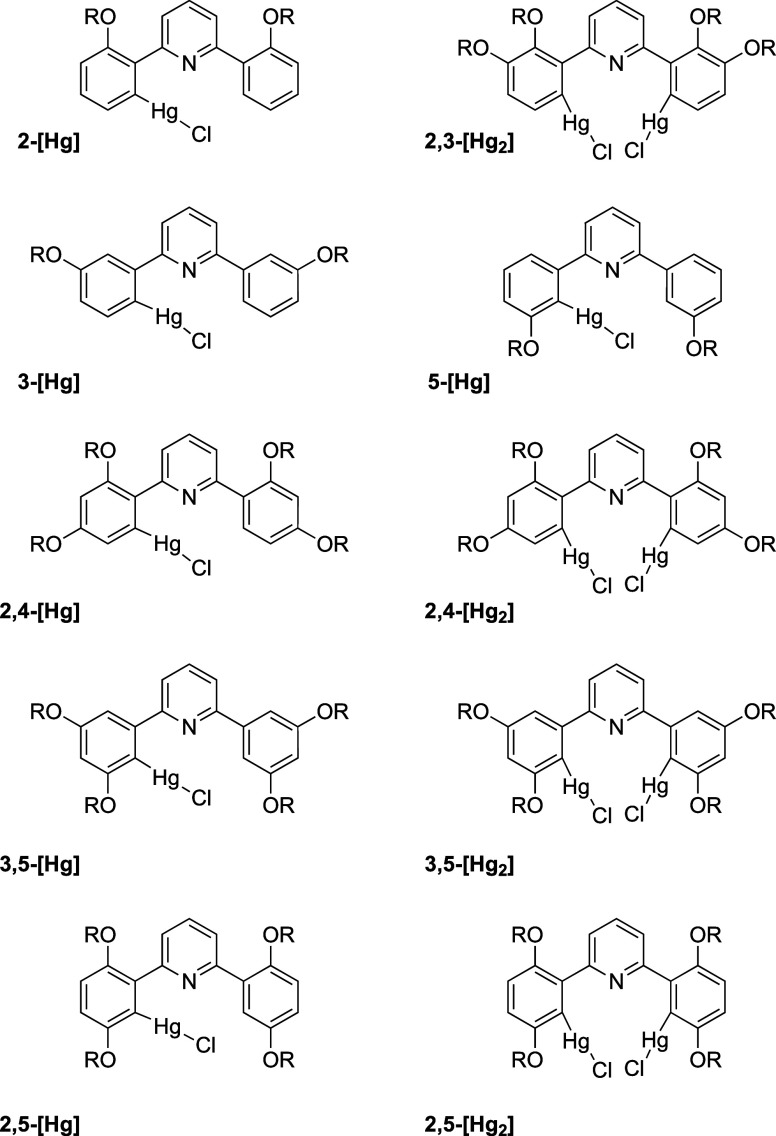
Structures of mercury(II) complexes. Note that in the diagram of **5-[Hg]**, the uncomplexed phenyl ring has been rotated about the Py–Ph bond to show the common orientation of the two alkoxy chains for ease of reference (R = C_12_H_25_).

As outlined above, mercury complexes are currently necessary if unfortunate (on account of their toxicity) intermediates en route to the target gold complexes. In previous work with **4-[L]** and **3,4-[L]**, they were obtained in yields of around 18–35% as impure, gray solids that were resistant to purification, even if their subsequent transmetalation to gold proceeded cleanly.^10,11^ The complexes are prepared (Scheme 2) by reaction of the appropriate ligand with 2 mol equiv of mercury(II) acetate and then working up with methanolic LiCl to give the chloromercury(II) product, whose analysis suggested that, despite the stoichiometry, the ligands were in fact monomercurated. Expecting the same gray powders with this new set of ligands, it was with some surprise that, using directly analogous conditions, it turned out to be possible to isolate pure mercury complexes and generally in high yields; their structures are found in Figure 3. Owing to the toxicity of the complexes, they were not worked up to absolute purity and so their essential integrity was established by NMR spectroscopy and mass spectrometry, with evaluation of purity coming from the former data. For some complexes, their identity was further confirmed by X-ray crystallography, and full details are found in the Supporting Information. As reported previously using **4-[L]** and **3,4-[L]**,^9^ the purity of the intermediate mercury complex appears not to influence the subsequent transmetalation reaction, with analytically pure gold complexes being obtained invariably. As such, the “casualty” of not knowing the precise purity of these intermediates is knowledge of an absolutely accurate yield.

All of the ligands formed complexes with mercury that were isolable in good to high yields (Table 1), even if some were obtained as mixtures. The two-chain ligands (**2-[L]**, **3-[L]**) gave only monomercurated products, noting that **3-[L]** gave rise to the isomeric complexes **3-[Hg]** and **5-[Hg]**, which could be separated by column chromatography. However, with the exception of **3,4-[L]**, which gave only a monomercurated complex, and **2,3-[L]**, which gave only a dimercurated complex, the other four-chain ligands gave both mono- and dimercurated products. Of the new ligands reported here, only **3,5-[L]** gave low isolated yields of the complex.

**Table 1:** Products and Yields on Formation of Two- and Four-Chain Mercury(II) Complexes

ligand	total yield (%)^a^	product
**2-[L]**	90	**2-[Hg]**
**3-[L]**	83	**3-[Hg]: 5-[Hg]** (0.7:1)
**4-[L]**	17	**4-[Hg]**
**3,4-[L]**	37	**3,4-[Hg]**
**2,3-[L]**	75	**2,3-[Hg**_**2**_**]**
**2,4-[L]**	57	**2,4-[Hg]: 2,4-[Hg**_**2**_**]** (1:1)
**2,5-[L]**	59	**2,5-[Hg]: 2,5-[Hg**_**2**_**]** (5:4)^b^
**3,5-[L]**	15	**3,5-[Hg]: 3,5-[Hg**_**2**_**]** (2:1)

a While data for **4-[L]** and **3,4-[L]** have been reported previously, yields recorded here reflect repreparation of the complexes.

b See also the discussion in the text.

As indicated above, given the toxicity of the complexes, few investigations were carried out to see how the ratio of mono- to dimercurated complex could be controlled, with the following exception. Thus, using **2,5-[L]**, the 5:4 ratio of **2,5-[Hg]**/ **2,5-[Hg**_**2**_**]** obtained under vigorous reflux became a 4:1 ratio under slightly less forcing conditions, although the overall yield remained unaffected. Likewise, a higher dilution similarly favored the formation of **2,5-[Hg]** over **2,5-[Hg**_**2**_**]**. Prepared together, the two complexes gave a mixture that proved inseparable. However, in attempting to realize complexes for characterization by X-ray crystallography, a variant of the ligand bearing butyloxy rather than dodecyloxy chains was used and here, the two complexes were separable by chromatography to give the dimercurated complex as a colorless solid and the monomercurated complexes as a colorless oil that solidified on standing.

**Figure 4:**
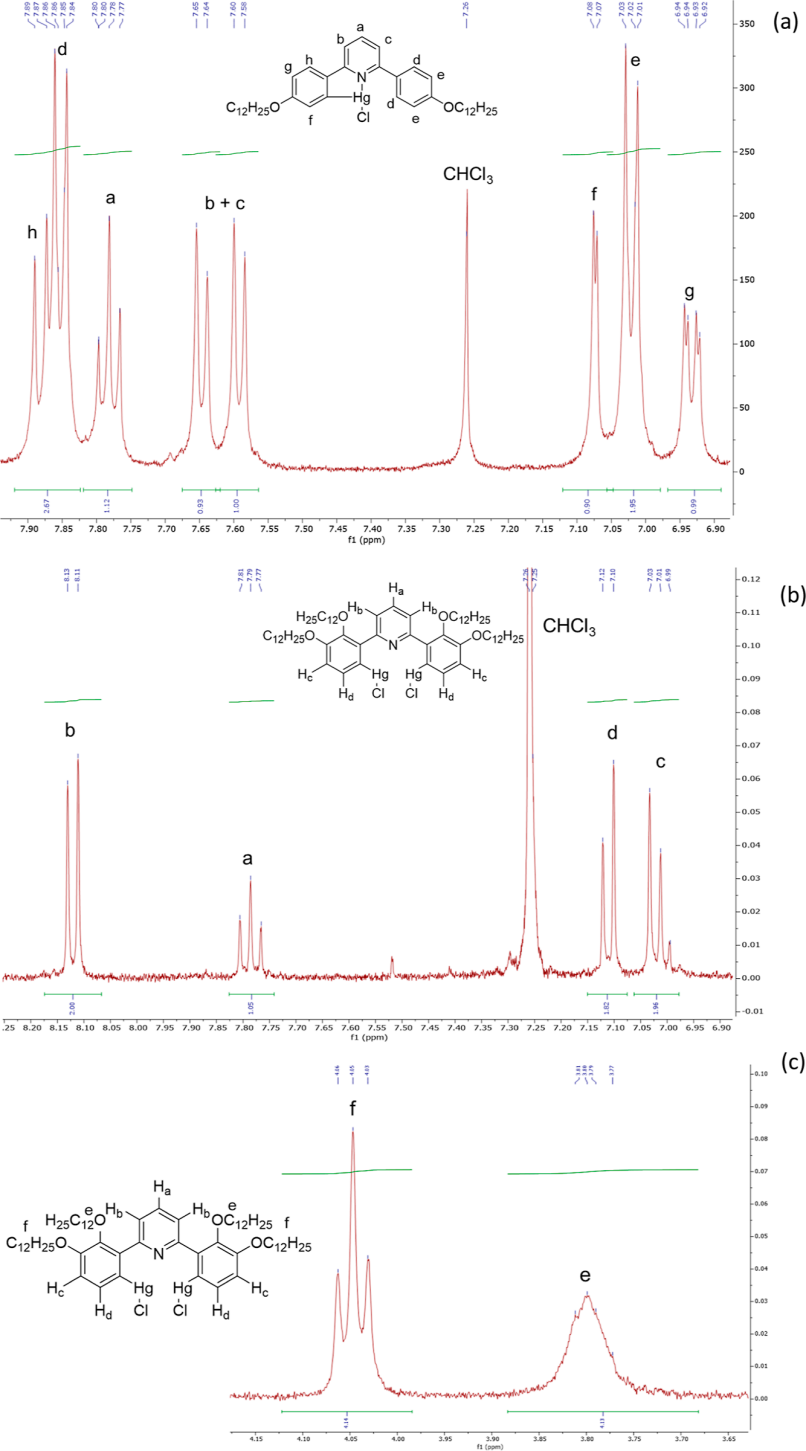
^1^H NMR spectrum (400 MHz) of (a) **4-[Hg]**, showing the aromatic region; (b) compound **2,3-[Hg**_**2**_**]**; and the (c) O–CH_2_—hydrogens of compound **2,3-[Hg**_**2**_**]**.

^1^H NMR spectroscopy was key to identifying the products, with the symmetric, dimercurated complexes giving spectra with relatively few signals and with one hydrogen resonance missing (site of mercuration) compared with the ligand. The spectra of the monomercurated products were, however, more complex reflecting the two different phenyl rings. For example, Figure 4a shows the assigned ^1^H NMR spectrum of **4-[Hg]** in the aromatic region, while Figure 4b shows the simpler spectrum for the dimercurated **2,3-[Hg**_**2**_**]**. ^1^H NMR spectroscopy was also instrumental in differentiating between the two monomercurated isomers of **3-[L]** (**3-[Hg]** and **5-[Hg]**—see Figures S1 and S2).

Interestingly, broadening was observed for one of the two oxymethylene (O–CH_2_) resonances in **2,3-[Hg**_**2**_**]**, (3.80 ppm) while the other remained a sharp triplet (4.05 ppm; Figure 4c). The two resonances were differentiated using NOE experiments, and the (sharp) lower-field resonance was assigned to the chains in the 3,3′-positions, while the broad, higher-field resonance was assigned to the chains in the 2,2′-positions. The crystal structure of **2,3-[Hg**_**2**_**]** (below—Figure 6) shows that the two metalated phenyl rings are twisted out of the plane of the central pyridyl ring (angles measured as 41 ± 0.5°). Libration of these rings would allow for the *O*-methylene hydrogens in the chains in the 2,2′-positions to sample quite different environments with the proximity of the pyridine ring, including over its face, which would account for the slight shielding. All this would imply that the noncovalent Hg–N interactions in the crystal structure (see below) do not persist in solution.

**Figure 5:**
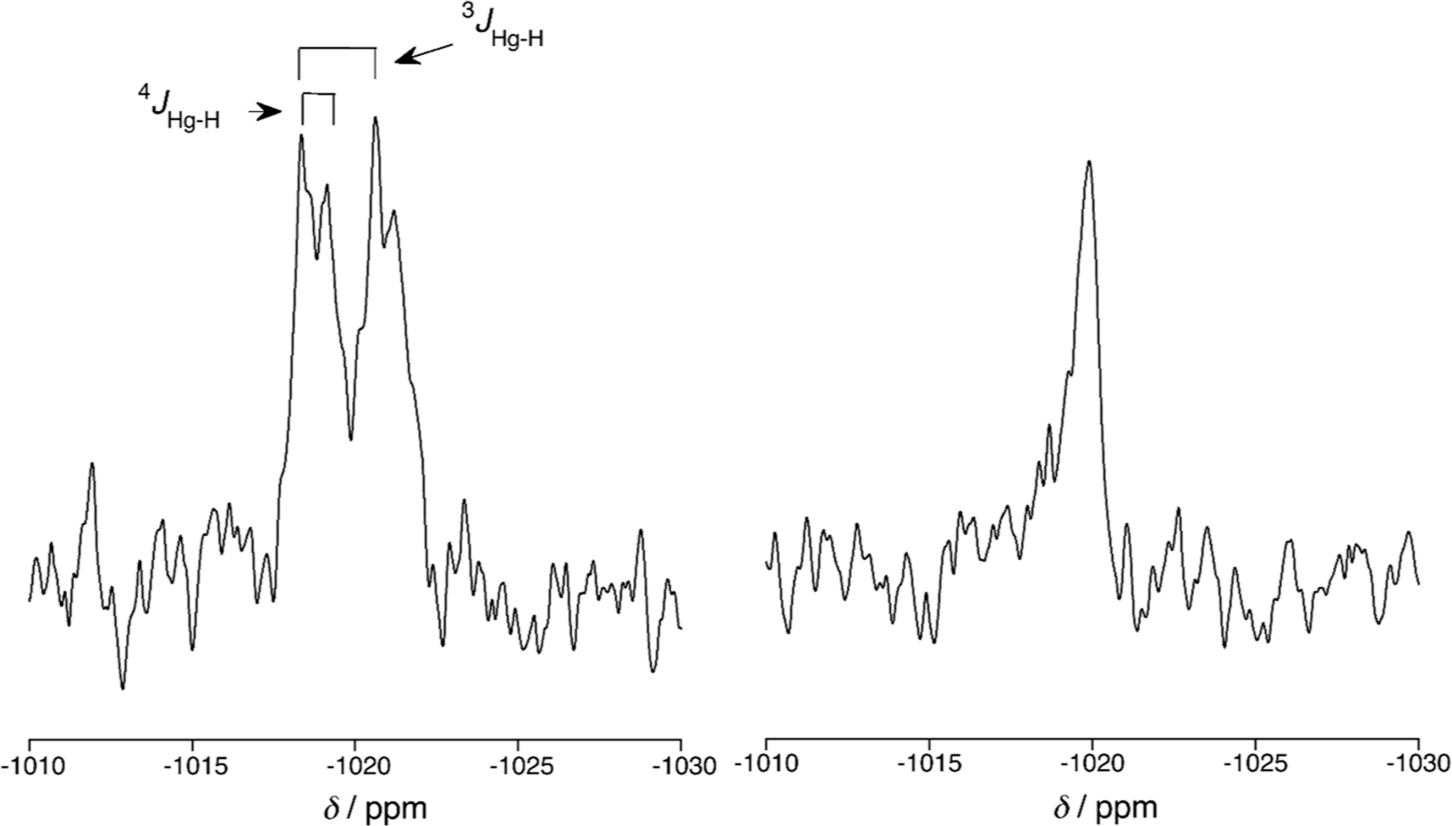
^199^Hg NMR spectrum (89.6 MHz) of **2-[Hg]**: (a) with hydrogen coupling and (b) with hydrogen decoupling.

The complexes were also characterized by ^199^Hg NMR spectroscopy (*I* = 1/2, 16.9% abundance), referenced externally to Hg(OAc)_2_ in D_2_O (−2351.48 ppm).^29^ Known chemical shifts for chloromercury(II) complexes^30^ were used in order to determine the spectral area of interest given the wide dispersion for δ(^199^Hg). Values of ^3^*J*_H–Hg_ were observed to be in a typical range for this type of coupling^31^ and are given where observed (**2-[Hg]** also showed ^4^*J*_H–Hg_), although interestingly they were not observed in the corresponding ^1^H spectra, neither was coupling to ^199^Hg observed in ^13^C spectra.^32,33^ This may be accounted for both by the relatively low abundance of ^199^Hg coupled with its large chemical shift anisotropy^33,34^ [indeed no coupling was observed in spectra recorded at 53.67 MHz (300 MHz instrument), compared to the 89.6 MHz otherwise used routinely]. Data are found in Table 2 and the ^199^Hg and ^199^Hg{^1^H} spectra of **2-[Hg]** are shown in Figure 5.

**Table 2:** ^199^Hg NMR Data for the Mercury(II) Complexes

complex	δ ^199^Hg/ppm	^3^*J*_H–Hg_/Hz
**4-[Hg]**	–1023 (d)	235
**2-[Hg]**	–1020 (dd)	203, 70 (^4^*J*_H–Hg_)
**3-[Hg]**	–991 (d)	191
**5-[Hg]**	–993 (s)	
**2,3-[Hg**_**2**_**]**	–1012 (d)	154
**2,5-[Hg**_**2**_**]**	–1010 (s)	
**2,5-[Hg]**	–1009 (s)	
**2,4-[Hg**_**2**_**]**	–1042 (s)^a^	
**2,4-[Hg]**	–1011 (s)^a^	
**3,5-[Hg**_**2**_**]**	–972 (s)^a^	
**3,5-[Hg]**	–954 (s)^a^	
**3,4-[Hg]**	–999 (s)	

a Neither pair of complexes could be separated into the two individual components and so chemical shifts are assigned as indicated in the text.

Despite the closely related nature of the complexes, the resonances are well-dispersed over around 100 ppm between *ca* −950 and −1040 ppm, consistent with an interaction with nitrogen as found in the spectra of related complexes where values of between −983 and −1069 ppm were found for chloromercury(II) complexes with a Hg–N interaction.^30^ The chemical shift is least negative where there is an alkoxy group in a conjugated position (3- and/or 5-) and conversely most negative when an alkoxy group is in a nonconjugated position (2- and/or 4-). This trend is emphasized where there are two alkoxy groups in one type of position and modulated when there is one in each type of position. These data will be discussed further.

### X-ray Single-Crystal Structures of the Mercury(II) Complexes

The structures of the three complexes were solved by X-ray methods following crystallization of the butoxy ligand homologues from a chloroform/ethanol solution. The structure of **2,3-[Hg**_**2**_**]** was reported previously, although some details are reprised here for purposes of comparison.^27^ For **2,5-[L]**, it was possible to obtain both mono- and dimercurated complexes.

#### Structure of **2,3-[Hg**_**2**_**]**

**Figure 6:**
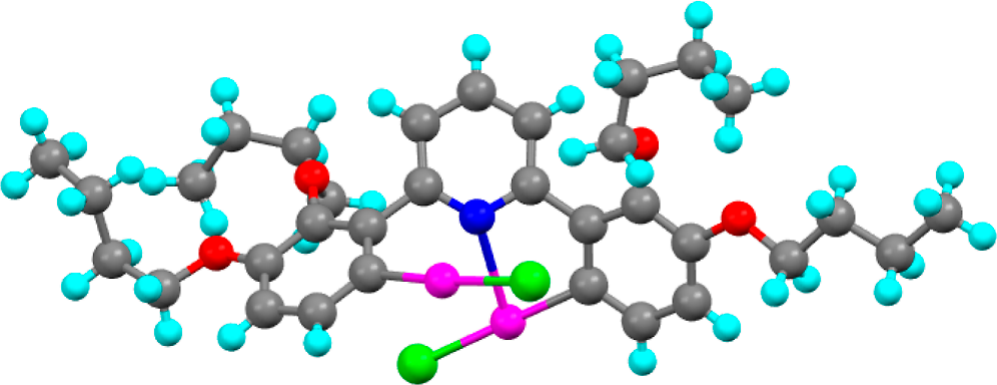
X-ray structure of one of the two complexes from the asymmetric unit of **2,3-[Hg**_**2**_**]** showing the shortest Hg–N distance [2.676(9) Å] and taken from ref (27).

Complex **2,3-[Hg**_**2**_**]** crystallized in the monoclinic space group *P*2/*c* with eight complexes in the unit cell based on two slightly different units (one of these is shown as Figure 6). Each mercury is coordinated in a linear fashion to carbon and a chloride ligand, with the angle at mercury being almost linear [179.0(3)° and 179.2(4)°]. Hg–C bond lengths are found to be 2.016(12), 2.027(14), 2.065(17), and 2.062(12) Å, all of which are statistically the same and of a magnitude very similar to those already in the literature.^32,35−39^ In addition, there is also a noncovalent interaction between each mercury and a pyridine nitrogen, and it is here where the two complexes in the asymmetric unit differ slightly. Thus, in one, the two Hg–N distances are identical with distances of 2.75(1) and 2.748(9) Å, whereas in the other, they are 2.767(9) and 2.676(9) Å, which are statistically different, the former being the same as those in the other complex with the latter being shorter. All of these interactions are between 81 and 84% of the sum of the van der Waals radii. The noncovalent interactions between mercury and chlorine are more involved and are described in the original publication. There is appreciable disorder toward the end of the chains.

### Structure of **2,5-[Hg**_**2**_**]**

Complex **2,5-[Hg**_**2**_**]** crystallized in triclinic space group *P*1̅, with three molecules in the asymmetric unit (six in the unit cell), with one exhibiting disorder in three of its butoxy chains (disorder removed for the purposes of diagrams but accessible in the deposited CIF file). For two of these, the whole chain was modeled in two positions with refined occupancies of 0.55:0.45(2) in one case and 0.538:0.462(17) in the other. For the third, just the terminal ethyl group was modeled in two positions with refined occupancies of 0.627:0.373(17). In the disordered chains, the C–C bond lengths were restrained to be equal.

**Figure 7:**
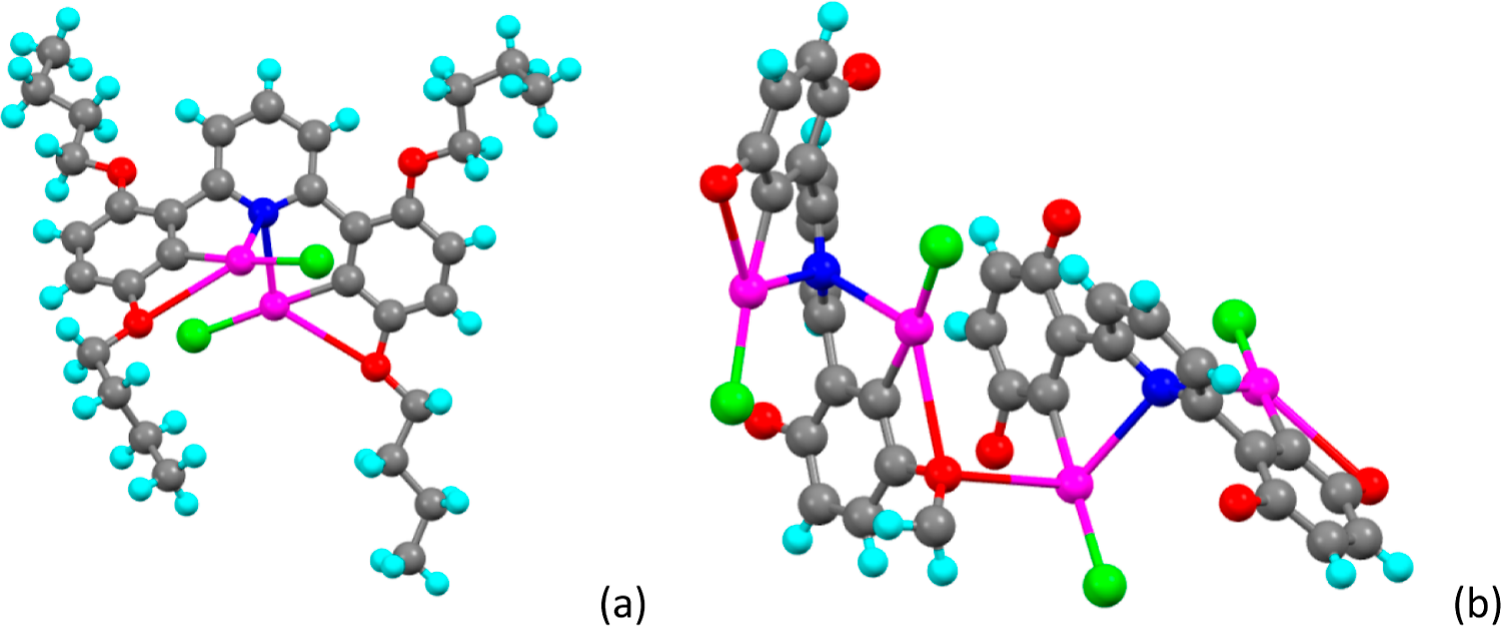
(a) Molecular structure of **2,5-[Hg**_**2**_**]** from X-ray crystallography; (b) arrangement of the two complexes linked through an intermolecular Hg–O interaction. Butyl chains are removed for clarity.

The six Hg–C bond lengths are identical [2.04(1) ± 0.02 Å] as are those of Hg–Cl [2.305(3) ± 0.03 Å], and in this structure, there are no short, noncovalent contacts between Cl and Hg. For one of the complexes in the asymmetric unit (Figure 7a), each mercury also has short contacts both to the pyridine nitrogen atom [2.664(8) Å and 2.763(8) Å] and to an alkoxy oxygen of the chains in the 5-positions [3.037(8) Å and 3.005(6) Å]. Of the remaining two complexes in the asymmetric unit, one is just as described above except that the pairs of Hg···N and Hg···O distances are the same [Hg···N = 2.729(9) and 2.701(9) Å; Hg···O = 3.00(1) and 2.956(8) Å]. However, for the other complex, while one mercury forms intramolecular coordination in a likewise manner [Hg···N = 2.827(8) Å; Hg···O 2.966(5) Å], the other mercury ion forms an intracomplex Hg···N interaction with the pyridyl nitrogen [2.767(7) Å] and two Hg···O interactions, one of which is the now expected intracomplex interaction at 3.041(9) Å in addition to an intercomplex interaction with the ether oxygen of the 5-butoxy chain of the neighboring complex [3.034(9) Å]. Representations of the individual units can be found in Figure S18. This is illustrated in Figure 7b in which the butyl elements of the chains have been removed for the sake of clarity.

### Structure of **2,5-[Hg]**

**Figure 8:**
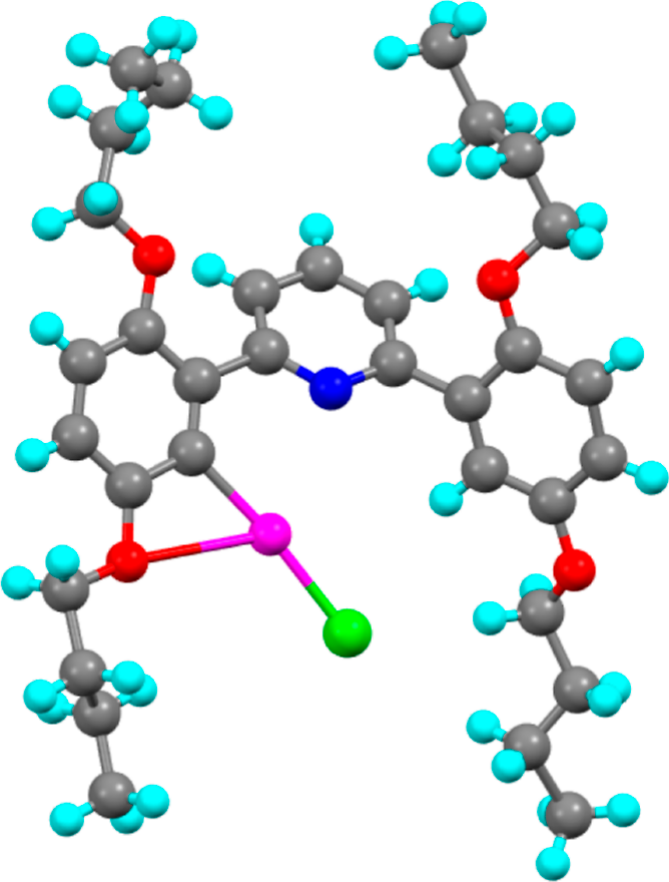
Molecular structure from X-ray crystallography of one complex of **2,5-[Hg]** from the asymmetric unit.

The complex **2,5-[Hg]** (Figure 8) also crystallized in the triclinic space group *P*1̅. However, it exhibited severe twinning, resulting in poor resolution and an *R*-factor > 12%. While this is good enough to establish the connectivity of the structure, the remaining discussion will be accordingly brief. There were three molecules in the asymmetric unit, with one exhibiting disorder about the central unit, within which the pyridine ring and mercury and chloride ions were modeled in two positions, with a refined occupancy of 0.568:0.432(3). In this disordered complex, there is an intramolecular Hg···O(ether) distance of 2.86(2) Å and a much longer Hg···N distance of 3.25(2) Å. Hg···O distances are similar in the other two nondisordered complexes [2.84(2) and 2.91(2) Å], as are the Hg···N distances [2.85(2) and 2.88(2) Å]. There are no short intercomplex interactions.

### Synthesis of Chlorogold(III) Complexes from Mercury(II) Precursors

The gold(III) complexes were prepared (Scheme 2) from the mono- and/or dimercury(II) precursors as exemplified in Figure 3 and using approaches described previously.^10^ Acetonitrile was used for the complexes substituted with two alkoxy chains, while solubility requirements dictated the use of a 1:1 mixture of chloroform/acetonitrile for complexes with four alkoxy chains. Where the mercury complex is isolated as an impure material (**4-[Hg]**, **3,4-[Hg]**, **2,4-[Hg]**, and **3,5-[Hg]**), the mixture is reacted with Na[AuCl_4_] without further purification and the resulting gold(III) complexes are obtained pure after either column chromatography, followed by crystallization, or by serial crystallizations as indicated in the Supporting Information. The identification system (Figure S3) mirrors that used for the ligands and mercury complexes.

### X-ray Structures of Gold(III) Complexes

Crystals suitable for single-crystal X-ray crystallography were obtained for several of the gold(III) complexes and are now described.

#### **2-[Au]-Cl**

**Figure 9:**
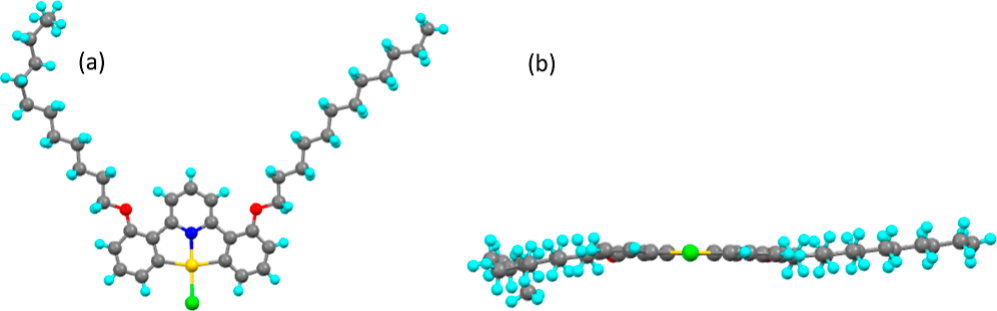
Molecular structure from X-ray crystallography of compound **2-[Au]-Cl** viewed (a) from above and (b) from the side. Disorder was removed for clarity.

Compound **2-[Au]-Cl** crystallized in the monoclinic crystal system in space group, *C*2/*c*. The crystal structure (Figure 9) shows that the complex is planar despite the steric clash between the 3-hydrogens on the pyridine ring and the alkoxy oxygens of the chains. The geometry is close to square planar, but to allow for the bite angle of the ligand, the CN[82.4(2) and 82.0(2)°] and ClC angles [97.8(2) and 97.8(2)°] deviate from 90°. The Au–N and Au–Cl bond lengths are 1.960(6) and 2.277(2) Å and are of a similar magnitude with other systems known in the literature.^10,40−45^ One of the dodecyl chains exhibited disorder from the carbon in the C8 position to the end of the chain.

#### **2,3-[Au]-Cl**

**Figure 10:**
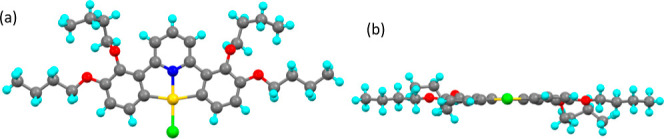
Molecular structure from X-ray crystallography of **2,3-[Au]-Cl**; (a) from above and (b) from the side showing the out-of-plane position of the butyloxy chain.

This structure has been reported previously^27^ but is reproduced here for the sake of completeness and comparison. Single crystals could not be obtained with dodecyloxy chains but were obtained when butyloxy groups were used instead. It crystallized in the monoclinic crystal system in space group *P*2_1_/*n* (Figure 10) and the asymmetric unit contains two molecules; the notionally square-planar geometry about gold is quite distorted. Interestingly and different from other structures, the butyloxy chains in the 2-position are twisted out of the plane of the molecule. There is some disorder toward the end of the chains.

#### **2,5-[Au]-Cl**

**Figure 11:**
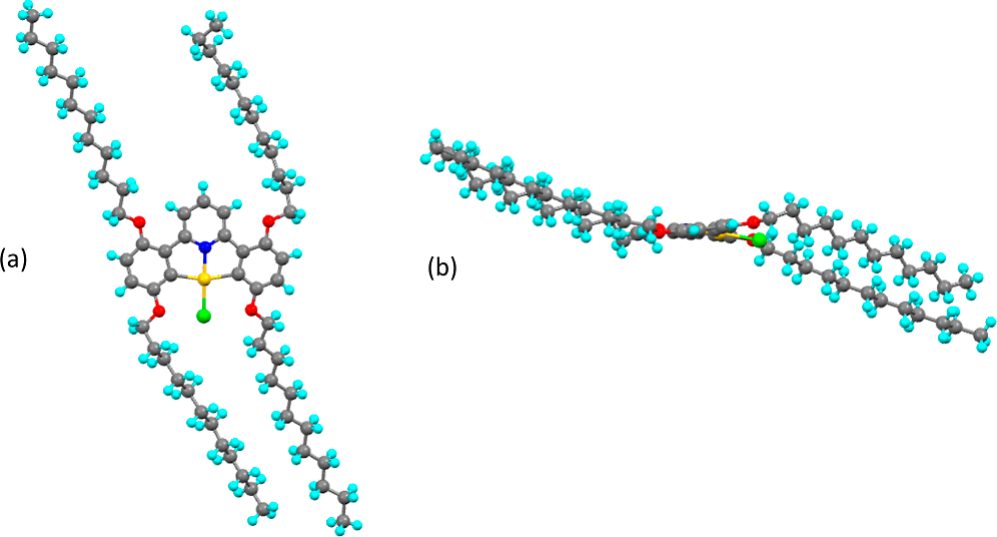
Molecular structure from X-ray crystallography of compound **2,5-[Au]-Cl**; (a) from above and (b) from the side.

The complex crystallized in a triclinic structure in space group *P*1̅. The crystal was layered, with slippage of the layers, resulting in very broad, streaked reflections (Figure S17) so that the final solution was of low quality with an *R*-factor of 16%. As such, while there is confidence in the overall disposition of the different parts of the molecule as shown in Figure 11, no other aspects of the structure will be discussed.

### ^1^H NMR Spectra of the Pyridyl Hydrogens in the Chlorogold(III) Complexes

**Figure 12:**
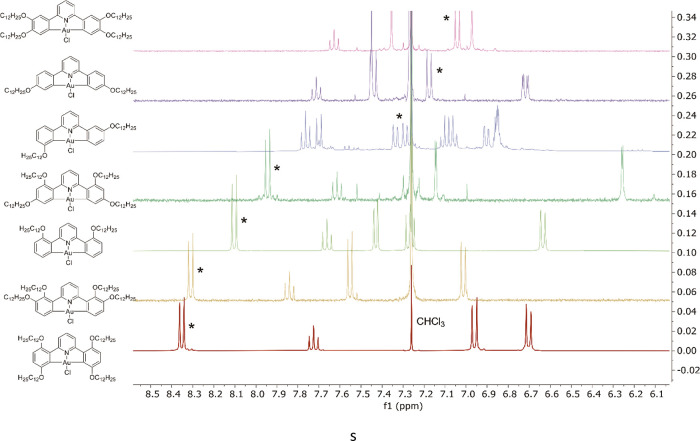
Aromatic region of the ^1^H NMR spectra (400 MHz) for all chlorogold(III) complexes in CDCl_3_. The *m*-pyridyl hydrogen resonance is indicated with “*”.

While, as described above, the pyridine hydrogens in the free ligands exhibit an eight-line AB_2_ multiplet, in the gold complexes, the same hydrogens show triplet (*p*-hydrogen) and doublet (*m*-hydrogens) resonances. On closer examination, the spectra reveal that while the chemical shift of the triplet of the *p*-pyridyl hydrogen varies only as 7.6 < δ < 7.9 ppm, that of the doublet corresponding to the *m*-pyridyl hydrogens varies much more as 7.04 < δ < 8.35 ppm (Figure 12). A similar wide variation in the chemical shift of an *m*-hydrogen on a pyridyl ring was also observed by Gutierrez et al. in a series of orthometalated di(μ-acetato)dipalladium(II) complexes prepared using a series of 2-phenylpyridines monofunctionalized on the phenyl ring with a range of electron-donating and -withdrawing substituents.^46^ The strongest influence on the chemical shift of the *m*-hydrogens is the presence of the oxygen from alkoxy chains in the 2,2′-positions, which leads to the greatest deshielding. Thus, the O···H separation, at between *ca* 2.2 and 2.3 Å in the three crystal structures reported here and in the previous publication,^27^ is shorter than the sum of the two van der Waals radii. However, the range of chemical shifts also shows that electronic effects are important, with very similar chemical shifts observed for **2,5-[Au]-Cl** and **2,3-[Au]-Cl**, being much more deshielded than the equivalent chemical shifts for **2-[Au]-Cl** and then **2,4-[Au]-Cl**. There is also a (smaller) isomer-dependent chemical shift dispersion for the three complexes that do not have 2,2′-alkoxy substitution.

Supporting the above assertions, consideration of the ^1^H NMR spectra for **2,5-[Hg**_**2**_**]** and **2,5-[Hg]** shows that in the former case where the 2-alkoxy chain on both (metalated) rings is held in closer proximity to the *m*-pyridyl hydrogen, its chemical shift is δ = 8.11 ppm, whereas in the latter case where the unmetalated ring is free to librate, two *m*-H chemical shifts are seen at δ = 8.11 ppm (metalated ring) and δ = 7.84 ppm (unmetalated ring).

### Isomer-Dependent Yields

**Scheme 3:**
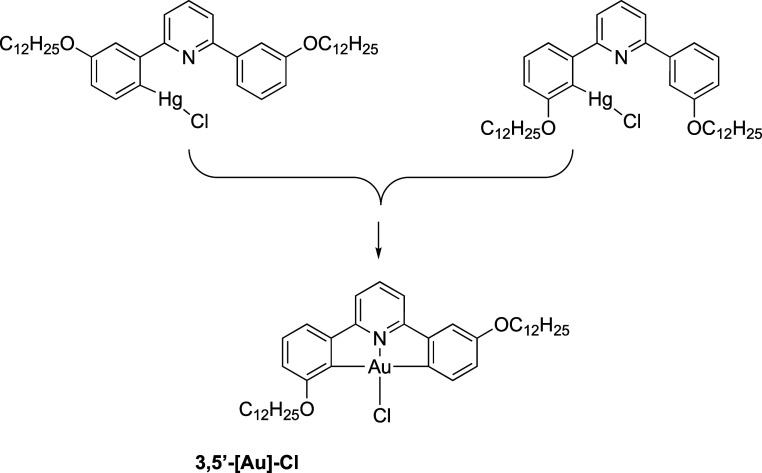
Formation of **3,5′-[Au]-Cl** Starting from a Mixture of **3-[Hg]** and **5-[Hg]**

The literature tends to show that yields of mercuration for cyclometalating C,N ligands can be quite high,^30,37,38,45^ but unfortunately, the literature for C,N,C ligands tends not to contain the same level of detail. The yields obtained here for the chlorogold(III) complexes depended on both the pattern of alkoxy chain substitution and the degree of mercuration (Table 3), so that dimercurated complexes displayed significantly lower yields on auration (<25%). Indeed, the ^1^H NMR spectra of the crude postreaction mixture indicated appreciable quantities of the unreacted dimercury complex in the preparation of **2,3-[Au]-Cl**, **2,5-[Au]-Cl**, and **2,4-[Au]-Cl**, suggesting preferential reaction of the monomercury complex. For **2,4-[Au]-Cl**, this led to real issues of purification as **2,4-[Hg**_**2**_**]** could not be removed via either column chromatography or crystallization. Consequently, while a pure sample of **2,4-[Au]-Cl** was eventually realized, the yield was very low (6%). The other difference in reactivity was observed starting from a mixture of **3-[Hg]** and **5-[Hg]**. Thus, while in principle three gold complexes could form (**3-[Au]-Cl**, **5-[Au]-Cl**, and **3,5′-[Au]-Cl**), in fact, only one—**3,5′-[Au]-Cl**—was isolated in *ca* 20% yield after chromatography along with unreacted **5-[Hg]**, suggesting strongly that **3-[Hg]** metalates preferentially while **5-[Hg]** is unreactive (Scheme 3). Interestingly, **3,5-[Hg]** also failed to transmetalate with only the unreacted mercury complex isolated postreaction. These latter observations are consistent with results obtained previously in a study of orthometalated 2-phenylpyridines.^46^ Here, 2-(3,5-dimethoxyphenyl)pyridine and palladium acetate did not react together, while using tetrachloropalladate(II) as the starting complex led to the N-bound *trans*, *bis*(η^1^-pyridine) complex. The authors attributed these findings to unfavorable steric factors, which would be consistent with the observations reported in this work.

**Table 3:** Yields of Auration Following Transmetalation and Total Overall Yield of the Gold Complexes Starting from the Free Ligand

starting complex	auration/%	overall yield from ligand/%
**2,3-[Hg**_**2**_**]**	24	18
**2-[Hg]**	40	36
**2,5-[Hg]/2,5-[Hg**_**2**_**]**^a^	17	10
**2,4-[Hg]**	6	3.5
**3,4-[Hg]**	13	5
**3,5-[Hg]/3,5-[Hg**_**2**_**]**^a^		0
**4-[Hg]**	48	8
**3-[Hg]/5-[Hg]**	20^b^	17

a Reaction started with the as-obtained mixture of the mono- and dimercurated complexes.

b Product obtained is **3,5′-[Au]-Cl**.

### Understanding the Reactivity

Given the unexpected formation of clean, isolable mercury complexes with some of these ligands, there was interest in trying to rationalize the observations in relation to the different substitution patterns of the alkoxy chains, which give rise to both steric and electronic effects. As such, ^13^C{^1^H} NMR data were recorded for the free ligands and their complexes with gold and mercury, and in each case, the resonance associated with the site of metalation was identified. Figure S19 shows that the trend in the chemical shifts with the substitution pattern is consistent across all three compound types and, in fact, the chemical shifts for complexes where there are both mono- and dimercurated examples are also in effect the same. The data are replotted in Figure S21 in the order of descending chemical shift for just the free ligands as indicative of the trend, and the variation of δ with the Hammett σ parameter is shown in Figure S21, showing a reasonable correlation as might be anticipated.

**Figure 13:**
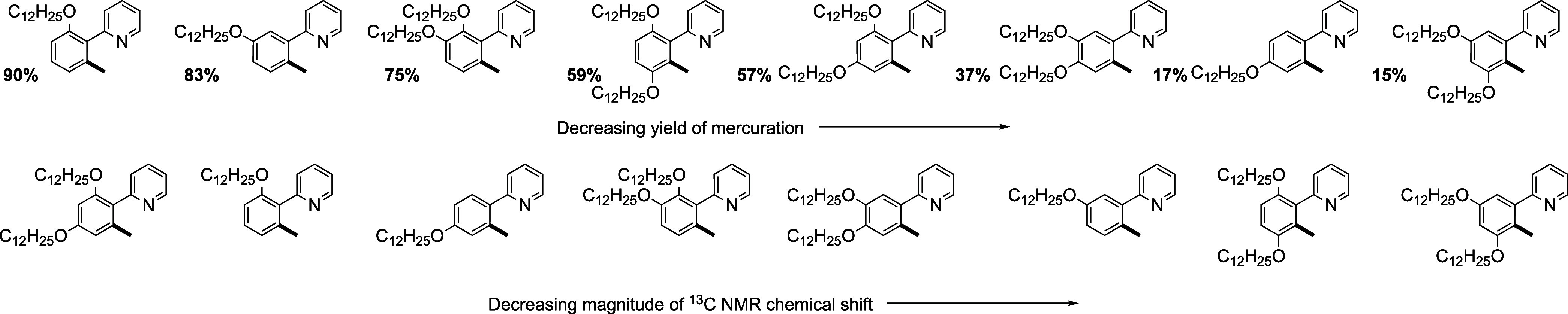
Juxtaposition of decreasing yield of mercuration (indicated in bold) and the magnitude of ^13^C NMR chemical shift for the ligands under study.

The data show that the greater the electron density at the metalating carbon, the lower the chemical shift. However, Figure 13 shows the same ordering of the ligands juxtaposed with decreasing yield of mercuration (using total yield where both mono- and dimercurated complexes are formed), and it is immediately apparent that the two are not correlated. This would suggest that electronic factors do not drive the metalation. This being the case, how might the relative yields of mercuration be understood? The ordering shows that four of the five highest yields are found where there is an alkoxy chain in the 2-position. (The exception is the ligand with the chain in the 3-position, but here there are two products, **3-[Hg]** and **5-[Hg]** and if the yields of these two isomers are considered individually, then they are 34 and 48%, respectively. This would place both of them lower than the lowest yield where there is a chain in the 2-position, observed for **2,4-[L]**.)

**Figure 14:**
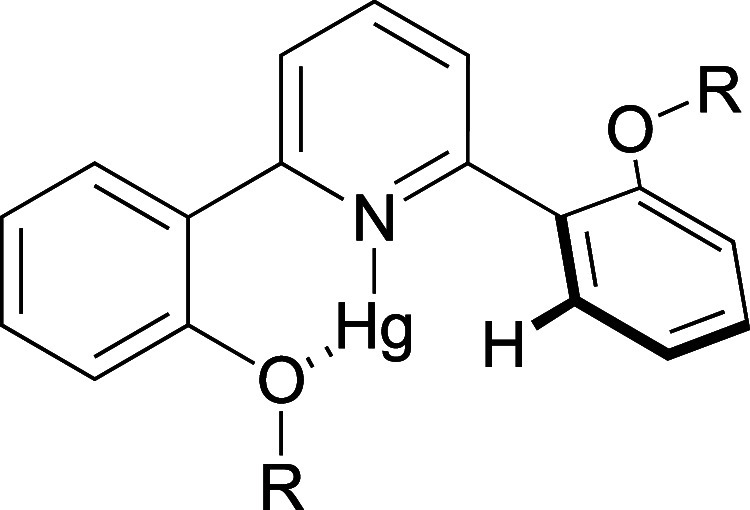
Postulated N,O-coordination of mercury to facilitate C–H activation.

If this is the case, then what might the mechanistic implications be? It is very likely that the first step in mercuration is binding of the mercury by pyridine nitrogen, followed by C–H activation. The single-crystal structures of both **2,5-[Hg]** and **2,5-[Hg**_**2**_**]** show the propensity for mercury also to bind to an ether oxygen and so it is possible to imagine an intermediate situation (Figure 14) in which an oxygen atom from an *ortho*alkoxy chain also binds the mercury. The other ring is of necessity twisted out of plane and through the mediation of the acetate ligand on the mercury, which is known to promote C–H activation,^47^ mercuration occurs. In the figure, no proposal is made regarding the possible orientation and binding of the acetate ligand, but its association with mercury and its known^47^ role in C–H activation are entirely consistent with the proposal made here. The fact that in the final complex, any Hg···N interactions are in effect of a stabilizing nature and are hence labile, indicates that the same mechanistic pathway can account for a second metalation.

### Alternative Routes to Auration

#### Attempted Direct Auration

While the literature contains examples of the direct auration of C–H bonds,^13−18,21−25^ none so far appears directly applicable across a wide range of ligand types.

The study described here is interesting as by changing the substitution pattern of alkoxy chains of the phenyl rings of 2,6-diphenylpyridine ligands, a rather remarkable reactivity with mercury emerges. Thus, whereas alkoxylation in the 4- or 3,4-positions of the phenyl rings in these ligands produced rather poor yields of impure organomercury compounds, with the chains in other positions, both mono- and dimercurated complexes are isolated pure and in very good yield. Given their propensity to mercurate directly, might they also be persuaded to aurate directly?

Attempts were made to aurate the ligands directly using in turn H[AuCl_4_], Na[AuCl_4_], and (Bu_4_N)[AuCl_4_] under the conditions used for transmetalation and, while ^1^H NMR evidence suggested the possibility of a very small conversion (≈1%), almost totally unreacted ligand was recovered after workup. In a different approach and based upon previous work,^19,20,48,49^ the ligand **2,5-[L]** was reacted with 2 mol equivalents of BuLi and the resulting solution treated with Na[AuCl_4_]. Unfortunately, under these conditions, reduction of the gold followed, as evidenced by the formation of a gold mirror.

Inspired by the published work of Tilset and co-workers^14−17^ and choosing **2,3-[L]** as the ligand, a series of experiments was undertaken under microwave irradiation in acetonitrile, aqueous acetonitrile, water, or ethanol and with or without an added acetate as base. Temperatures used were in the range of 100–160 °C and while [AuCl_4_]^−^ was the main source of gold, some experiments were undertaken using [AuCl(tht)] (tht = tetrahydrothiophene) instead. None of these reactions yielded any product.

#### Transmetalation via a Palladium Intermediate

The “mercury drop test” is a widely accepted indicator of the participation of nanoparticulate palladium in, for example, cross-coupling reactions. However, Gorunova et al.^31^ reported that the reaction of soluble *ortho*palladated complexes with metallic mercury led smoothly to analogous, soluble *ortho*mercurated complexes through a simple transmetalation. As such, they demonstrated that catalysis by *soluble* palladium complexes can also be inhibited by the addition of mercury. This prompted the thought that if gold can displace mercury and mercury can displace palladium, then can gold displace palladium, too?

**Scheme 4:**
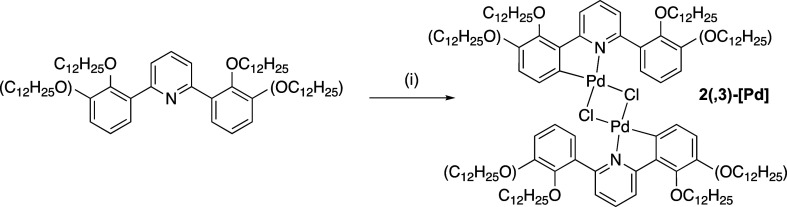
Preparation of the Dimeric, Orthometalated Palladium(II) Complexes: (i) Na_2_[PdCl_4_], EtOH, Δ

**Figure 15:**
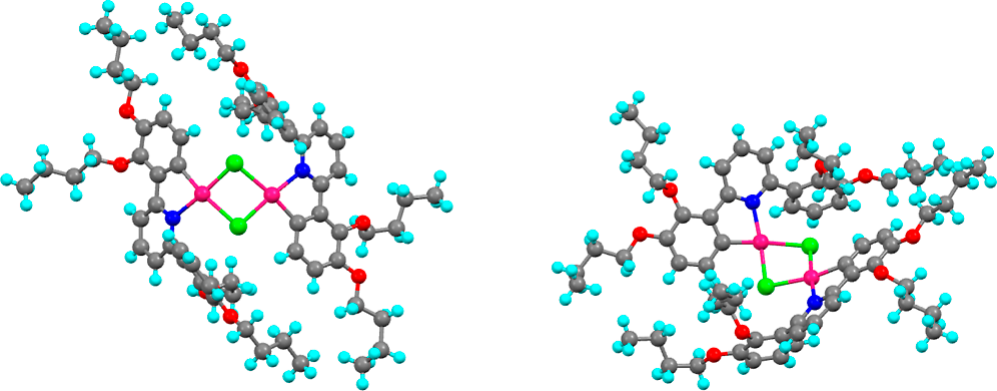
Two views of the structure of the μ-chloro dimer of **2,3-[Pd]**.

In order to demonstrate the proof of concept, two examples of orthopalladated ligands were prepared using **2-[L]** and **2,3-[L]** (Scheme 4). The unoptimized reaction led to the recovery of **2,3-[Pd]** and **2-[Pd]** in isolated yields of 33 and 14%, respectively, and the complexes were characterized by ^1^H NMR spectroscopy and mass spectrometry, while the structural integrity was confirmed by X-ray crystallography for the butyloxy homologues of **2,3-[Pd]**. This complex (Figure 15) crystallized in the *P*1̅ space group as a diethyl ether solvate, which sat in the cleft arising from the bent structure of the complex (Figure S22). The diethyl ether of crystallization was disordered and was modeled in two positions with refined occupancies of 0.708:0.292(10) and pairs of bonds in the disordered ether were restrained to be equal.

The structure shows an *ortho*metalated 2-phenylpyridine unit, with the other phenyl ring of the ligand uncomplexed and twisted out of the way of the central Pd_2_Cl_2_ unit (the angle between the plane of the coordinated pyridine and the plane of the noncoordinated phenyl ring is 51.5 ± 0.5°; there are two as they are not formally the same). The trans bond angles at the two palladiums are slightly distorted from a linear arrangement with the two ClCl angles being inequivalent at 170.4(2) and 173.0(2)°, while the NCl angles are also inequivalent at 173.7(1) and 171.0(1)° (in each case, the first angle of the pair refers to the same palladium in the structure). The two PdPd bond angles are equivalent at 92.1 ± 0.1°. While the four ligated atoms and the palladium to which they are bound are not perfectly coplanar, an idea of the bend that pivots about the μ_2_-Cl_2_ bridge is obtained by defining an average plane of the ligated atoms (N, C, and 2 × Cl) for each and determining the angle subtended, which is 132.5°.

These palladium complexes were then reacted with Na[AuCl_4_] in 1:1 CHCl_3_/MeCN and in each case, the corresponding gold complex was obtained, albeit in modest to poor, if unoptimized, yields (48% for **2,3-[Au]-Cl** and 14% for **2-[Au]-Cl**). Notwithstanding the cost of palladium salts, in comparison to the use of organomercury intermediates, this represents an attractive alternative route for exploration.

#### Rhodium-Mediated Catalytic Auration

Relatively recently, Martín et al. reported on the direct auration of a series of 2-phenylpyridine ligands in the presence of catalytic quantities of [Cp*RhCl_2_]_2_.^26^ This rhodium(III) complex shows a great propensity to undergo *ortho*metalation reactions with potentially chelating C,N ligands and, in conjunction with sodium benzoate as base, mediates catalytically the direct auration of these ligands using Na[AuCl_4_]. The nature of the mediation of the rhodium complex was nicely demonstrated by both isolation of the *ortho*rhodated ligand and its subsequent reaction with tetrachloroaurate(III) to give the target complex. Application of this methodology to the ligands described here, however, led to only an aurated product with **2,3-[L]** and in a low yield of 11%. Thus, a reaction that is successful with a range of 2-phenylpyridines giving yields of up to 86% so far appears less effective, modest when the substrate is a sterically more demanding 2,6-diphenylpyridine. As such, this transformation is currently under further investigation.

**Scheme 5:**
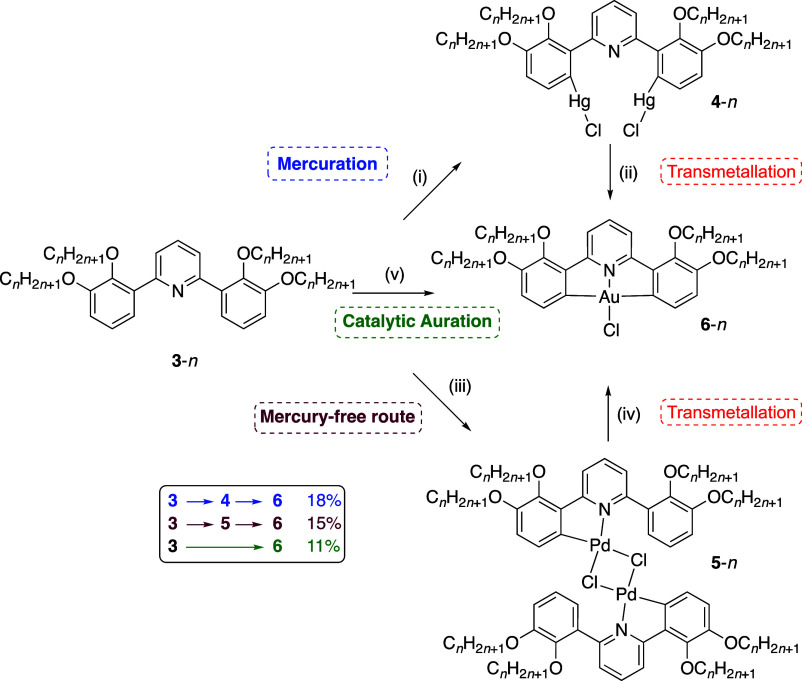
Summary of the Various Interconversions and Routes to Gold(III) Complexes of Alkoxylated Diphenylpyridines Conditions are found in the Supporting Information.

The various interconversions and routes to the target gold complexes are summarized in Scheme 5, which is modified slightly from the form used previously.^27^

## Summary and Conclusions

In expanding the range of 2,6-(mono- and dialkoxyphenyl)pyridines available for the preparation of chlorogold(III) complexes, a quite remarkable reactivity toward mercury(II) emerged. Mercurated ligands are required as intermediates for the subsequent preparation of the gold complexes via transmetalation, but whereas they are normally obtained in low to moderate yield as rather impure materials, here it was possible to obtain them pure and in moderate to high yields, with examples of both mono- and dimercurated complexes formed. Three complexes were characterized crystallographically (one monomercurated and two dimercurated). All showed the expected linear C–Hg–Cl coordination, and in addition, the mercury was stabilized by donation from the pyridine nitrogen as well as an alkoxy oxygen in one example. ^199^Hg NMR chemical shifts reflected the number and positions of the alkoxy groups on the pyridine ring to which the mercury was bound, with three-bond (and occasionally four-bond) coupling to hydrogen seen in most spectra. To the best of our knowledge, the reactivity represented here in forming dimer-curated complexes is without precedent. Subsequent auration gave lower yields when starting from dimercurated complexes and, in particular, **3,5-[Hg]** showing no evidence of auration, while use of a mixture of **3-[Hg]** and **5-[Hg]** implied that the latter was unreactive in this transmetalation reaction.

Consideration of the reactivity of the ligands toward mercuration revealed that high yields were obtained when a 2-alkoxy group was present. This was interpreted as the reaction proceeding through a preferred mechanistic pathway that would stabilize the mercury through chelate binding to the pyridine nitrogen and 2-alkoxy oxygen. In turn, this would facilitate C–H activation by the acetate ligand from the mercury starting material, with the mercury now well-positioned to bind to the activated carbon.

This unusual ligand reactivity rekindled interest in the possibility of achieving direct auration, but none of the routes attempted, which included the use of microwaves as developed by Tilset and co-workers, was successful. However, stimulated by the report of the formation of mercury complexes from palladium precursors, it proved possible to “run this reaction in reverse”, so that reaction of **2-[L]** and **2,3-[L]** with [PdCl_4_]^2–^ led to a dimeric, chloro-bridged, *ortho*palladated complex, which then underwent subsequent transmetalation with gold. As such, a mercury-free route to the gold complexes was demonstrated.

It also proved possible to deploy the rhodium(III)-catalyzed reaction reported by Martín et al., to realize a direct auration reaction using [AuCl_4_]^−^. However, the yields obtained with the 2,6-diphenylpyridines which are the subject of this paper are much lower than those found with 2-phenylpyridines, which suggests that there are still modifications that are possible in order to realize a very general auration protocol for tridentate C,N,C pincers.

Finally and at the request of one of the referees, we undertook analysis of residual mercury levels in samples of the chlorogold complexes (**2-[Au]-Cl**, **2,3-[Au]-Cl**, **2,5-[Au]-Cl**, and **3,4-[Au]-Cl**) prepared via transmetalation from their mercury analogues. The results varied between 0.02 and 0.94 wt %, at which level CHN data would remain within accepted limits. We do not know what form mercury takes as the initial step in the analysis is achieved via microwave-promoted acid digestion (see Supporting Information). While these are small numbers, they are not insignificant. We are not aware of such measurements having been reported previously and so, as is common with other groups, we have not taken particular steps to see the extent to which the complexes can be prepared mercury-free. It would then be of interest to determine systematically and in some detail the extent to which, for example, palladium or rhodium was carried over where these metals are used to mediate auration to understand how general the phenomenon might be. A second question might be the extent to which small, residual levels of these metals, in whatever form they take, affect the properties of any devices fabricated subsequently. All of this underlines the importance of understanding in detail routes to potentially applicable aurated complexes in order to realize target materials in high purity and under the most accessible of conditions.

## Data Availability

Full experimental data are found in the attached Supporting Information, including the single-crystal structures of the ligands and full synthetic details for the new compounds. There is also a data archive available at 10.15124/f4fb8d38-5d37-4343-9e7a-b5f642db4c8a, which contains all of the native NMR files as well as the other analytical data. CIF and checkCIF files are also available, and the structures [CCDC 2283590–2283602] are deposited at the Cambridge Crystallographic Database.

## Supporting Information Available

Details of instrumentation used; preparative details, NMR, and other analytical data for the new compounds; single-crystal structures of the ligands; temperature-dependent NMR spectra for **2,3-[L]**; and additional figures to supplement those in the main manuscript (PDF)

## Footnotes

a NMR data files for the ligands and complexes, including ^1^H, ^13^C{^1^H} and where appropriate ^199^Hg/^199^Hg{^1^H} as well as many 2D experiments, are collected in the freely available data archive referred to in the Data Availability Statement.
