# Supplementary material for: Gold(III), Mercury(II), and Palladium(II) Complexes of a Series of Isomeric Bis(mono- and dialkoxyphenyl)pyridines: Introduction of Gold through Transmetalation and Catalysis
Source: Inorg Chem. 2024 Apr 18;63(17):7589–603. doi: 10.1021/acs.inorgchem.3c03791 (PMC11061838; doi:10.1021/acs.inorgchem.3c03791)
Supplement: Supplementary file 1 — ic3c03791_si_001.pdf [file ic3c03791_si_001.pdf]

Supporting Information

Gold(III), Mercury(II) and Palladium(II) Complexes of a Series of Isomeric Bis(Mono- and Di-alkoxy-phenyl)pyridines: Introduction of Gold through Transmetallation and Catalysis

Alice Jane McEllin, Christopher A. Goult, Golam Mohiuddin, Liam J. Curtis, Theo F. N. Tanner, Adrian C. Whitwood, Jason M. Lynam* and Duncan W. Bruce*

Department of Chemistry
University of York
Heslington
YORK YO10 5DD (UK)
Tel: (+44) 1904 324085
E-mail: duncan.bruce@york.ac.uk

Instrumentation

^{1}H and $^{13}C\{^{1}H\}$ NMR spectra were recorded on a Jeol ECS400 spectrometer operating at 400 MHz, with chemical shifts referenced to appropriate residual non-deuterated solvent signal. ^{1}H and $^{13}C\{^{1}H\}$ NMR spectra, NOE measurements and temperature-dependent ^{1}H NMR spectra were recorded on a Bruker 500 AVANCE II spectrometer operating at 500 MHz, while ^{199}Hg NMR spectra were recorded on the same instrument at 89.6 MHz, being externally referenced to $Hg(OAc_2)$ in D_2O. A Bruker pulse program (*zg)* was used to give proton-coupled spectra, while their *zgpg30* pulse program gave proton-decoupled spectra. ^{19}F NMR spectra were recorded on a Jeol ECS400 spectrometer operating at 376.5 MHz.

APCI mass spectroscopy data was collected on a Bruker compact® time of flight mass spectrometer and MALDI-TOF on a Bruker Ultraflex III mass spectrometer, using the University of York Chemistry department MS service (Karl Heaton). Spectra were internally calibrated using sodium formate calibrant and samples were transferred to the spectrometer using an Agilent 1260 Infinity LC system. Elemental analysis (CHN) used an Exeter Analytical Inc. CE-440 analyser.

Diffraction data were collected at 110 K on an Oxford Diffraction SuperNova dual-source X-ray diffractometer with MoKα radiation (λ = 0.71073 Å) and CuKα radiation (λ = 1.54184 Å) using an EOS CCD camera. The crystal was cooled with an Oxford Instruments Cryojet. Diffractometer control, data collection, initial unit cell determination, frame integration and unit-cell refinement were carried out with 'Crysalis'.[3] Face-indexed absorption corrections were applied using spherical harmonics, implemented in SCALE3 ABSPACK scaling algorithm.[4] OLEX2[5] was used for overall structure solution, refinement and preparation of computer graphics and publication data. Within OLEX2,[5] the algorithms used for structure solution were ShelXT. Refinement by full-matrix least-squares used the SHELXL-97[6] algorithm within OLEX2.[5] All non-hydrogen atoms were refined anisotropically. Hydrogen atoms were placed using a 'riding model' and included in the refinement at calculated positions. In the figures of the molecular structures carbon atoms are coloured grey, chlorine green, gold yellow, hydrogen turquoise, nitrogen blue, mercury deep pink, oxygen red, silicon pale yellow, and zinc pink.

For mercury analysis, samples were digested by microwave digestion in Anton-Paar Multiwave Go Plus with HNO_3 (2.50 cm^3, 70% w/w, ThermoFisher AR grade) at ramp rate 20 °C min^{-1}, ultimate temperature 190 °C, dwell time 20 min. Samples were filtered with 0.22 μm filter (Chromatography Direct) and diluted with ultrapure water (Milli-Q type 1 ultrapure water system supplied by Merck).

Samples were analysed using an Agilent ICP-OES 5800 VDV spectrometer, using reference standard CCS-6 supplied by Inorganic Ventures traceable to NIST certified reference materials. Hg measurements were made at 184.887 nm in axial configuration. All standards were matrix matched to the digestion media. EDTA disodium salt was added at 1.5% w/v to the wash solution to minimise memory effect.

Synthesis - Ligands

4-[L]-OMe: 2,6-Bis(4-methoxyphenyl)pyridine

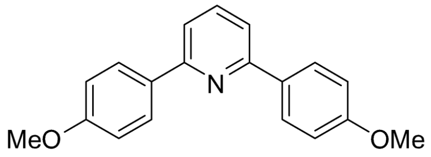

2,6-Dibromopyridine (12.21 g, 51.5 mmol) and 4-methoxyphenyl boronic acid (23.47 g, 154.5 mmol) were added to a flask containing [$Pd_3(OAc)_6$] (65.0 mg, 0.5 mol%) and K_3PO_4 (32.80 g, 154.5 mmol). Ethylene glycol (400 mL) was added, and the reaction mixture heated to 80 °C for 1.5 hr with vigorous stirring. The reaction mixture was then cooled to room temperature and the produced solid was isolated by filtration and washed with water (200 mL), after which it was air dried. The resulting grey solid was dissolved in warm CH_2Cl_2 (~400 mL), filtered through Celite® and reduced to dryness. The solid crystalline, off-white residue was crystallised from the minimum amount of hot ethanol to give the pure product as colourless crystals. Yield: 7.81 g (52%)

^{1}H NMR (400 MHz, $CDCl_3$) δ^H ppm: 8.11 (4H, AA'XX', J = 8.6 Hz), 7.74 (1H, t, J = 7.5 Hz), 7.57 (2H, d, J = 7.8 H), 7.02 (2H, AA'XX', J = 8.7 Hz), 3.88 (6H, s).

4-[L]-OH: 2,6-*Bis*(4-hydroxyphenyl)pyridine

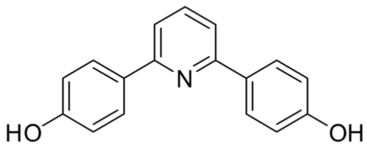

2,6-*Bis*(4-methoxyphenyl)pyridine (5.23 g, 18.0 mmol) was added to molten pyridinium chloride (26.85 g, 232.3 mmol) at 200 °C and stirred for 16 hr. The still molten mixture was added carefully to distilled water (350 mL) and the resulting yellow precipitate was isolated by filtration, air-dried and was used without further purification. Yield: 5.20 g (110%, product contaminated with water).

^{1}H NMR (400 MHz, d^6-DMSO) δ^H ppm: 9.85 (2H, s), 8.02 (4H, AA'XX', *J* = 8.3 Hz), 7.89 (1H, t, *J* = 8.8 Hz), 7.73 (2H, d, *J* = 7.8 H), 6.90 (4H, AA'XX', *J* = 8.8 Hz).

4-[L]: 2,6-Bis(4-dodecyloxyphenyl)pyridine

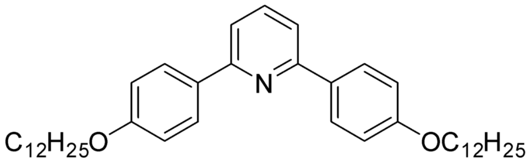

2,6-*Bis*(4-hydroxyphenyl)pyridine (5.20 g, 19.75 mmol), 1-bromododecane (11.9 mL, 12.31 g, 94.39 mmol) and K_2CO_3 (8.1783 g, 59.18 mmol) were heated to 90 °C in DMF (300 mL) for 16 hr. The reaction mixture was cooled to room temperature and the off-white solid was isolated by filtration, washed with water (300 mL) and acetone (60 mL) before being left to air dry. Yield: 7.00 g (59%)

^{1}H NMR (400 MHz, $CDCl_3$) δ^H ppm: 8.09 (4H, AA'XX', *J* = 9.0 Hz), 7.73 (1H, t, *J* = 8.2 Hz), 7.56 (2H, d, *J* = 7.8 H), 7.00 (2H, AA'XX', *J* = 8.9 Hz), 4.02 (4H, t, *J* = 6.0 Hz), 1.81 (4H, m), 1.46 (4H, m), 1.27 (32H, broad m), 0.88 (6H, t, $^3J_{HH}$ = 7.0 Hz).

^{13}C{^{1}H} NMR (125.8 MHz, $CDCl_3$) δ^C ppm: 160.01, 156.37, 137.22, 132.07, 128.15, 117.05, 114.57, 68.11, 31.92, 29.66, 29.63, 29.60, 29.58, 29.41, 29.35, 29.28, 26.05, 22.68, 14.11.

2-[L]-OMe: 2,6-*Bis*(2-methoxyphenyl)pyridine

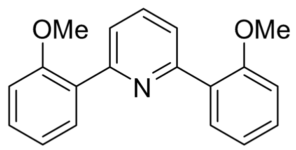

2,6-Dibromopyridine (12.20 g, 51.5 mmol) and 2-methoxyphenyl boronic acid (23.48 g, 154.5 mmol) were added to a flask containing [$Pd_3(OAc)_6$] (60.0 mg, 0.5 mol%) and K_3PO_4 (32.82 g, 154.5 mmol). Ethylene glycol (400 mL) was added, and the reaction mixture heated to 80 °C for 1.5 hr with vigorous stirring, then the reaction mixture was cooled to room temperature. The grey precipitated solid was recovered by filtration and washed with water (150 mL), after which it was air dried. The solid was dissolved in warm CH_2Cl_2 and filtered through Celite®. The filtrate was collected and, following solvent removal, the crystalline solid, off-white residue was crystallised from the minimum amount of hot ethanol to give the pure product as colourless needle-like crystals. Yield: 12.86 g (86%)

^{1}H NMR (400 MHz, $CDCl_3$) δ^H ppm: 7.97 (2H, dd, $^3J_{HH}$ = 7.6 Hz, $^4J_{HH}$ = 1.8 Hz), 7.77 (2H, AB_2), 7.77 (1H, AB_2), 7.39 (2H, ddd, $^3J_{HH}$ = 7.4 Hz, $^3J_{HH}$ = 8.3 Hz, $^4J_{HH}$ = 1.8 H), 7.12 (2H, ddd, $^3J_{HH}$ = 7.4 Hz, $^3J_{HH}$ = 8.3 Hz, $^4J_{HH}$ = 1.8 Hz), 7.03 (2H, dd, $^3J_{HH}$ = 8.3 Hz, $^4J_{HH}$ = 0.9 Hz), 3.89 (6H, s).

^{13}C{^{1}H} NMR (100.5 MHz, $CDCl_3$) δ^H ppm: 157.01, 155.32, 135.11, 131.42, 129.61, 129.46, 123.03, 120.97, 111.29, 55.54.

2-[L]-OH: 2,6-*Bis*(2-hydroxyphenyl)pyridine

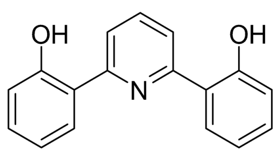

2,6-*Bis(*2-methoxyphenyl)pyridine (11.53 g, 39.57 mmol) was added to molten pyridinium chloride (64.90 g, 554.1 mmol) at 200 °C and stirred for 18 hr. The still molten mixture was carefully added to distilled water (350 mL) and the resulting beige precipitate was isolated by filtration, air-dried and was used without further purification. Yield: 10.25 g (93%)

^{1}H NMR (400 MHz, $CDCl_3$) δ^H ppm: 10.03 (2H, br s), 8.01 (1H, t, $^3J_{HH}$ = 8.3 Hz), 7.72 (2H, AB_2), 7.72 (2H, d, $^3J_{HH}$ = 8.0 Hz), 7.69 (2H, dd, $^3J_{HH}$ = 7.8 Hz, $^4J_{HH}$ = 1.7 Hz), 7.36 (2H, AB_2), 7.03 (2H, d, $^3J_{HH}$ = 8.3 Hz, $^4J_{HH}$ = 0.8 Hz), 7.01 (2H, ddd, $^3J_{HH}$ = 8.0 Hz, $^3J_{HH}$ = 8.0 Hz, $^4J_{HH}$ = 0.8 Hz).

2-[L]: 2,6-*Bis*(2-dodecyloxyphenyl)pyridine

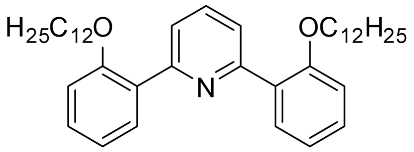

2,6-*Bis(*2-hydroxyphenyl)pyridine (10.10 g, 37.98 mmol), 1-bromododecane (17.25 mL, 23.66 g, 94.95 mmol) and K_2CO_3 (15.76 g, 113.9 mmol) were heated to reflux in 2-butanone (100 mL) for 67 hr, then filtered and reduced to dryness *in vacuo* to give an orange oil, which was crystallised from the minimum amount of hot methanol to give colourless plates. Yield: 3.47 g (64%)

^{1}H NMR (400 MHz, $CDCl_3$) δ^H ppm: 7.98 (2H, dd, $^3J_{HH}$ = 7.6 Hz, $^4J_{HH}$ = 1.8 Hz), 7.78 (2H, AB_2), 7.68 (1H, AB_2), 7.33 (2H, ddd, $^3J_{HH}$ = 8.3 Hz, $^3J_{HH}$ = 7.4 Hz, $^4J_{HH}$ = 1.8 H), 7.06 (2H, ddd, $^3J_{HH}$ = 7.6 Hz, $^3J_{HH}$ = 7.6 Hz, $^4J_{HH}$ = 1.1 Hz), 6.99 (2H, dd, $^3J_{HH}$ = 8.4 Hz, $^4J_{HH}$ = 1.2 Hz), 4.03 (4H, t, $^3J_{HH}$ = 6.5 Hz), 1.79 (4H, m), 1.44 (4H, m), 1.35-1.15 (32H, br m), 0.88 (6H, t, $^3J_{HH}$ = 6.8 Hz)

$^{13}C\{^1H\}$ NMR (100.5 MHz, $CDCl_3$) δ^H ppm: 156.69, 155.25, 134.82, 131.49, 129.59, 129.55, 123.02, 120.85, 112.46, 68.58, 31.91, 29.66, 29.63, 29.58, 29.56, 29.35, 29.32, 29.27, 26.13, 22.68, 14.11

ESI MS (m/z): Expected for $C_{41}H_{62}NO_2$ = 600.4775; Observed: 600.4759 $[M + H]^+$ (Error = 1.6 mDa)

3-[L]-OMe: 2,6-*Bis*(3-methoxyphenyl)pyridine

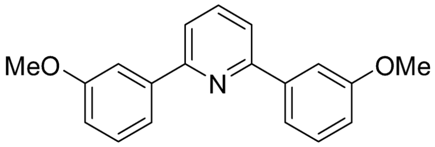

2,6-Dibromopyridine (12.20 g, 52.5 mmol) and 3-methoxyphenyl boronic acid (23.48 g, 154.5 mmol) were added to a flask containing $[Pd_2(OAc)_6]$ (61.6 mg, 0.5 mol%) and K_3PO_4 (32.80 g, 154.5 mmol). Ethylene glycol (400 mL) was added, and the reaction mixture heated to 80 °C for 2 hr with vigorous stirring. The reaction mixture was cooled to room temperature and the black, tar-like

solid was isolated by filtration and washed with water (150 mL), after which it was air dried. The resulting solid was dissolved in warm CH_2Cl_2 (~100 mL) and filtered through Celite®. The filtrate was collected and the solvent removed under reduced pressure to give an off-white solid, which was crystallized from the minimum amount of hot ethanol to give the pure product as white needle-like crystals. Yield = 13.29 g (89%)

^{1}H NMR (400 MHz, $CDCl_3$) δ^H ppm: 7.81 (1H, AB_2), 7.75 (2H, dd, $^4J_{HH}$ = 2.8 Hz, $^4J_{HH}$ = 1.6 Hz), 7.70 (2H, ddd, $^3J_{HH}$ = 7.8 Hz, $^4J_{HH}$ = 1.7 Hz, $^4J_{HH}$ = 0.9 Hz), 7.69 (2H, dd, J = 8.0 Hz, J = 7.6 Hz), 7.41 (2H, AB_2), 6.89 (2H, ddd, $^3J_{HH}$ = 8.3 Hz, $^4J_{HH}$ = 2.6 Hz, $^4J_{HH}$ = 0.9 Hz), 3.92 (6H, s).

^{13}C NMR (100.6 MHz, $CDCl_3$) δ^C ppm: 159.97, 1566.42, 140.88, 137.42, 129.62, 119.35, 118.87, 119.35, 114.60, 112.43, 55.29.

3-[L]-OH: 2,6-*Bis*(3-hydroxyphenyl)pyridine

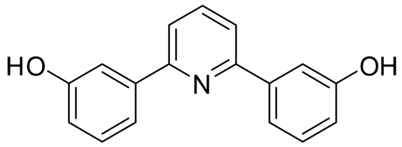

2,6-*Bis*(3-methoxyphenyl)pyridine (12.50 g, 42.90 mmol) was added to molten pyridinium chloride (62.35 g, 539.8 mmol) at 200 °C and stirred for 16 hours. The still molten mixture was added to distilled water (200 mL) and the resulting treacle-like dark brown oil was isolated by filtration and air-dried. It was used without further purification. Yield: 11.59 g (103%, product contaminated with water)

^{1}H NMR (400 MHz, DMSO-d_6) δ^H ppm: 9.65 (2H, br s), 7.92 (1H, AB_2), 7.82 (1H, d, J = 7.2 Hz), 7.82 (1H, d, J = 8.4 Hz), 7.63 (2H, dd, $^4J_{HH}$ = 2.5 Hz, $^4J_{HH}$ = 1.6 Hz), 7.57 (2H, ddd, $^3J_{HH}$ = 7.8 Hz, $^4J_{HH}$ = 1.6 Hz, $^4J_{HH}$ = 1.1 Hz), 7.31 (2H, AB_2), 6.85 (2H, ddd, $^3J_{HH}$ = 8.0 Hz, $^4J_{HH}$ = 2.5 Hz, $^4J_{HH}$ = 1.2 Hz).

3-[L]: 2,6-*Bis*(3-dodecyloxyphenyl)pyridine

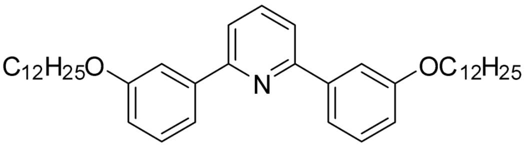

2,6-*Bis*(3-hydroxyphenyl)pyridine (11.30 g, 26.18 mmol), 1-bromododecane (48 mL, 65.28 g, 261.9 mmol) and K_2CO_3 (57.89 g, 418.9 mmol) were heated to reflux in 2-butanone (500 mL) for 21 hr, then cooled to room temperature and filtered to isolate a brown filtrate. On reduction to dryness to a brown oil was achieved, which was purified *via* column chromatography (silica, petroleum ether (40-60 °C), R_f = 0.00, then CH_2Cl_2, R_f = 0.91) to give a yellow oil, which solidified on standing. The product was crystallised from hot ethanol to give small pale yellow needle-like crystals, which were filtered, washed with ethanol and air dried. Yield: 15.23 g (97%)

^{1}H NMR (400 MHz, $CDCl_3$) δ^H ppm: 7.80 (1H, AB_2), 7.73 (2H, dd, $^4J_{HH}$ = 2.6 Hz, $^4J_{HH}$ = 1.6 Hz), 7.69 (2H, ddd, $^3J_{HH}$ = 7.7 Hz, $^4J_{HH}$ = 1.7 Hz, $^4J_{HH}$ = 1.0 Hz), 7.68 (2H, dd, J = 7.4 Hz, J = 8.3), 7.39 (2H, AB_2), 6.87 (2H, ddd, $^3J_{HH}$ = 8.2 Hz, $^4J_{HH}$ = 2.6 Hz, $^4J_{HH}$ = 1.0 Hz), 4.07 (4H, $^3J_{HH}$ = 6.5 Hz), 1.83 (4H, m), 1.49 (4H, m), 1.4-1.2 (32H, br m), 0.88 (6H, t, $^3J_{HH}$ = 6.9 Hz).

$^{13}C\{^1H\}$ NMR (100.6 MHz, $CDCl_3$) δ^C ppm: 159.53, 159.52, 140.84, 137.32, 129.54, 11.17, 118.79, 115.16, 113.06, 68.01, 31.89, 29.66, 29.62, 29.60, 29.59, 29.51, 29.43, 29.33, 26.07, 22.66, 14.08.

3,4-[L]-OMe: 2,6-*Bis*(3,4-dimethoxyphenyl)pyridine

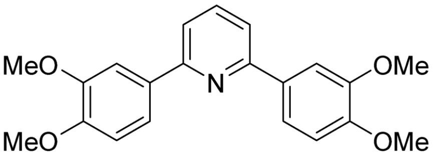

2,6-Dibromopyridine (6.75 g, 28.46 mmol) and 3,4-dimethoxyphenyl boronic acid (15.55 g, 85.37 mmol) were added to a flask containing $[Pd_3(OAc)_6]$ (24.7 mg, 0.5 mol%) and K_3PO_4 (18.19 g, 85.37 mmol). Ethylene glycol (200 mL) was added, and the reaction mixture heated to 80 °C for 1.5 hr with vigorous stirring. The reaction mixture was cooled to room temperature, isolated by filtration and washed with water (150 mL), after which it was air dried. The resulting grey solid was dissolved in warm CH_2Cl_2 (~75 mL) and filtered through Celite®. The filtrate was collected and the solvent removed under reduced pressure. The solid, off-white residue was crystallised from the

minimum amount of hot ethanol to give the pure product as colourless needle-like crystals. Yield: 9.20 g (92%)

^{1}H NMR (400 MHz, $CDCl_3$) δ^H ppm: 8.84 (2H, d, $^4J_{HH}$ = 2.0 Hz), 7.75 (1H, t, $^3J_{HH}$ = 8.1 Hz), 7.65 (2H, dd, $^3J_{HH}$ = 8.0 Hz, $^4J_{HH}$ = 2.0 Hz), 7.60 (2H, d, $^3J_{HH}$ = 7.9 Hz), 6.98 (2H, d, $^3J_{HH}$ = 8.4 Hz), 4.01 (6H, s), 3.95 (6H, s).

3,4-[L]-OH: 2,6-*Bis*(3,4-dihydroxyphenyl)pyridine

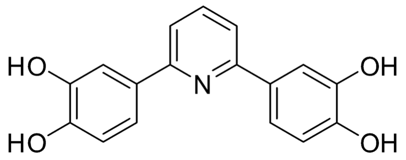

2,6-*Bis*(3,4-dimethoxyphenyl)pyridine (12.78 g, 36.37 mmol) was added to molten pyridinium chloride (53.93 g, 466.68 mmol) at 200 °C and stirred for 16 hr. The still molten mixture was carefully added to distilled water (350 mL) and the resulting bright yellow precipitate was isolated by filtration, air-dried, and was used without further purification. Yield: ~10 g (product heavily contaminated with water).

^{1}H NMR (400 MHz, d^6-DMSO) δ^H ppm: 9.20 (4H, s), 7.82 (1H, t, $^3J_{HH}$ = 8.2 Hz), 7.64 (2H, d, $^4J_{HH}$ ~ 2.0 Hz), 7.63 (2H, d, $^3J_{HH}$ ~ 7.8 Hz), 7.55 (2H, dd, $^3J_{HH}$ = 8.2 Hz, $^4J_{HH}$ = 2.0 H), 6.85 (2H, d, $^3J_{HH}$ = 8.3 Hz).

3,4-[L]: 2,6-*Bis*(3,4-didodecyloxyphenyl)pyridine

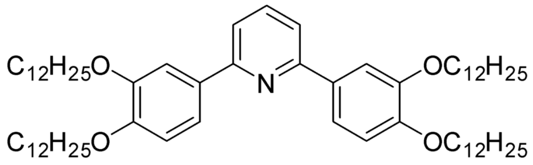

2,6-*Bis*(3,4-dihydroxyphenyl)pyridine (10 g, 4 mmol), 1-bromododecane (54.0 mL, 56.05 g, 224.9 mmol), K_2CO_3 (58.67 g, 424.5 mmol) were heated to 90 °C in DMF (400 mL) for 16 hr. The reaction mixture was cooled to room temperature and the solid precipitate isolated by filtration, washed with water (700 mL) and acetone (160 mL) and left to air dry to give a tan solid. Crude recrystallisation from hot ethanol, then filtered and washed with ethanol, distilled water (200 mL) and acetone (60 mL) to give an off-white solid. Subsequently the product was crystallised from hot

ethanol, filtered and washed with ethanol and acetone (60 mL) to give an off-white solid, then finally crystallised from hot CH_2Cl_2 and ethanol to give small off-white needle-like crystals. Yield: 22.42 g (69%)

^{1}H NMR (400 MHz, $CDCl_3$) δ^H ppm: 7.80 (2H, d, $^4J_{HH}$ = 2.1 Hz), 7.72 (1H, t, $^3J_{HH}$ = 8.2 Hz), 7.62 (2H, dd, $^3J_{HH}$ = 8.4 Hz, $^4J_{HH}$ = 2.0 Hz), 7.56 (2H, d, $^3J_{HH}$ = 7.8 Hz), 6.97 (2H, d, $^3J_{HH}$ = 8.4 Hz), 4.12 (4H, t, $^3J_{HH}$ = 6.6 Hz), 4.06 (4H, t, $^3J_{HH}$ = 6.7 Hz), 1.85 (8H, m), 1.49 (8H, m), 1.27 (64H, broad m), 0.87 (6H, t, $^3J_{HH}$ = 7.0 Hz), 0.87 (6H, t, $^3J_{HH}$ = 7.0 Hz).

^{13}C{^{1}H} NMR (100.6 MHz, $CDCl_3$) δ^C ppm: 156.23, 150.10, 149.17, 137.18, 132.49, 119.55, 117.28, 113.38, 112.52, 69.23, 69.19, 31.91, 29.72, 29.69, 29.66, 29.63, 29.49, 29.37, 29.25, 26.11, 2602, 22.68, 14.11.

2,3-[L]-OMe: 2,6-*Bis*(2,3-dimethoxyphenyl)pyridine

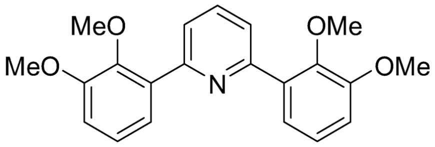

2,6-Dibromopyridine (6.74 g, 28.46 mmol) and 2,3-dimethoxyphenyl boronic acid (15.54 g, 85.37 mmol) were added to a flask containing [$Pd_2(OAc)_6$] (22.0 mg, 0.5 mol%) and K_3PO_4 (18.12 g, 85.37 mmol). Ethylene glycol (200 mL) was added, and the reaction mixture heated to 80 °C for 2 hr with vigorous stirring. The reaction mixture was cooled to room temperature, and the solid precipitate isolated by filtration and washed with water (150 mL), after which it was air dried. The resulting grey solid was dissolved in warm CH_2Cl_2 (~50 mL) and filtered through Celite® and reduced to dryness. The cubic crystalline, off-white residue was crystallised from the minimum amount of hot ethanol to give the pure product as white broad rectangular crystals. Yield: 8.91 g (89.1%)

^{1}H NMR (400 MHz, $CDCl_3$) δ^H ppm: 7.81 (3H, AB_2), 7.51 (2H, dd, $^3J_{HH}$ = 7.9 Hz, $^4J_{HH}$ = 1.6 Hz), 7.17 (2H, dd, $^3J_{HH}$ = 8.0 Hz, $^3J_{HH}$ = 8.0 Hz), 6.97 (2H, d, $^3J_{HH}$ = 8.2 Hz, $^4J_{HH}$ = 1.5 Hz),), 3.92 (6H, s), 3.72 (6H, s).

$^{13}C\{^{1}H\}$ NMR (100.5 MHz, $CDCl_3$) δ^H ppm: 155.23, 152.97, 147.20, 136.02, 134.59, 124.18, 123.02, 122.88, 112.46, 60.97, 55.92.

2,3-[L]-OH: 2,6-*Bis*(2,3-dihydroxyphenyl)pyridine

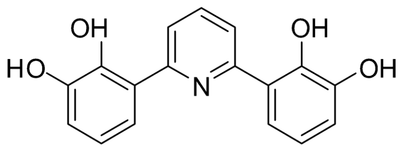

2,6-*Bis*(2,3-dimethoxyphenyl)pyridine (8.50 g, 24.19 mmol) was added to molten pyridinium chloride (38.93 g, 336.9 mmol) at 200 °C and stirred for 16 hr. The still molten mixture was added carefully to distilled water (350 mL) and the resulting mustard yellow precipitate was isolated by filtration, air-dried and used without further purification. Yield: 6.42 g (90%).

^{1}H NMR (400 MHz, $CDCl_3$) δ^H ppm: ~10.18 (2H, s br), 8.01 (1H, t, $^{3}J_{HH}$ = 8.2 Hz), 7.75 (2H, d, $^{3}J_{HH}$ = 8.0 Hz), 7.25 (2H, dd, $^{3}J_{HH}$ ~ 8 Hz, $^{4}J_{HH}$ = 1.4 Hz), 7.04 (2H, dd, $^{3}J_{HH}$ = 7.8 Hz, $^{4}J_{HH}$ = 1.4 Hz), 6.93 (2H, dd, $^{3}J_{HH}$ = 8.0 Hz, $^{3}J_{HH}$ = 7.9 Hz), 5.78 (2H, s br).

2,3-[L]: 2,6-*Bis*(2,3-didodecyloxyphenyl)pyridine

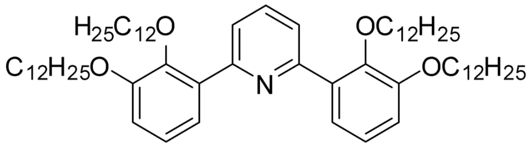

2,6-*Bis*(2,3-dihydroxyphenyl)pyridine (6.40 g, 21.33 mmol), 1-bromododecane (32.4 mL, 33.63 g, 134.9 mmol) and K_2CO_3 (47.21 g, 138.3 mmol) were heated to reflux in 2-butanone (200 mL) for 144 hr, then filtered and reduced to dryness *in vacuo* to give an orange oil, which solidified to a brown solid, which was crystallised from the minimum amount of hot methanol to give a red-brown solid. The product was purified *via* column chromatography (silica, 95:5 petroleum ether (40-60 °C):ethyl acetate, R_f = 0.35) to give an orange oil, which solidified on standing to give an off-white solid. The product was crystallised from hot hexane/ethyl acetate twice to give a white crystalline solid. Yield: 13.42 g (64%)

^{1}H NMR (400 MHz, $CDCl_3$) δ^H ppm: 7.83 (2H, AB_2), 7.70 (1H, AB_2), 7.47 (2H, dd, $^3J_{HH}$ = 7.8 Hz, $^4J_{HH}$ = 1.3 Hz), 7.12 (2H, dd, $^3J_{HH}$ = 8.2 Hz, $^3J_{HH}$ = 8.2 Hz), 6.94 (2H, dd, $^3J_{HH}$ = 7.9 Hz, $^4J_{HH}$ = 1.3 Hz), 4.02 (4H, t, $^3J_{HH}$ = 6.5 Hz), 3.80 (4H, t, $^3J_{HH}$ = 6.6 Hz), 1.86 (4H, m), 1.59 (4H, m), 1.51 (4H, m), 1.27 (68H, broad m), 0.88 (6H, t, $^3J_{HH}$ = 7.2 Hz), 0.88 (6H, t, $^3J_{HH}$ = 7.0 Hz).

^{13}C{^{1}H} NMR (100.6 MHz, $CDCl_3$) δ^C ppm: 155.45, 152.59, 146.57, 135.28, 135.11, 123.93, 123.34, 122.69, 113.37, 73.77, 68.68, 31.91, 30.19, 29.71, 29.70, 26.69, 29.68, 29.66, 29.63, 29.52, 29.45, 29.36, 29.17, 26.03, 22.67, 14.10.

2,3-[L]-C4: 2,6-*Bis*(2,3-dibutoxyphenyl)pyridine

2,6-*Bis*(2,3-dihydroxyphenyl)pyridine (1.4083 g, 4.7692 mmol), 1-bromobutane (9.5 mL, 12.12 g,

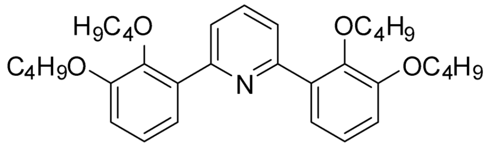

88.47 mmol) and K_2CO_3 (10.23 g, 74.02 mmol) were heated to reflux in 2-butanone (100 mL) for 89 hr. Then the resulting solution was filtered, washed with acetone (50 mL) and reduced to dryness *in vacuo* to give an orange oil. The product was purified *via* column chromatography (silica, 95:5 petroleum ether (40-60 °C):ethyl acetate, R_f = 0.23) to give an orange oil, then on application of a second column to give a viscous yellow oil. Yield: 1.165 g (53%)

^{1}H NMR (400 MHz, $CDCl_3$) δ^H ppm: 7.83 (2H, AB_2), 7.71 (1H, AB_2), 7.46 (2H, dd, $^3J_{HH}$ = 7.9 Hz, $^4J_{HH}$ = 1.5 Hz), 7.12 (2H, dd, $^3J_{HH}$ = 7.9 Hz, $^3J_{HH}$ = 7.9 Hz), 6.94 (2H, dd, $^3J_{HH}$ = 8.1 Hz, $^3J_{HH}$ = 1.6 Hz), 4.03 (4H, t, $^3J_{HH}$ = 6.6 Hz), 3.80 (4H, t, $^3J_{HH}$ = 6.6 Hz), 1.85 (4H, m), 1.55 (8H, m), 1.30 (4H, m), 1.00 (6H, t, $^3J_{HH}$ = 7.3 Hz), 0.88 (6H, t, $^3J_{HH}$ = 7.3 Hz).

2,4-[L]-OMe: 2,6-*Bis*(2,4-dimethoxyphenyl)pyridine

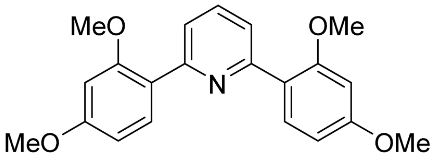

2,6-Dibromopyridine (6.74 g, 28.46 mmol) and 2,4-dimethoxyphenyl boronic acid (15.54 g, 85.37 mmol) were added to a flask containing [$Pd_2(OAc)_6$] (24.9 mg, 0.5 mol%) and K_3PO_4 (18.44 g, 86.87 mmol). Ethylene glycol (400 mL) was added, and the reaction mixture heated to 80 °C for 1.5 hr with vigorous stirring. The reaction mixture was cooled to room temperature and the solid precipitate isolated by filtration and washed with water (150 mL), and ethanol (50 mL), after which it was air dried. The resulting grey solid was dissolved in warm CH_2Cl_2 (~125 mL) and filtered through Celite®. The filtrate was collected and the solvent removed under reduced pressure. The crystalline, off-white residue was crystallised from the minimum amount of hot ethanol to give the pure product as white needle-like crystals. Yield = 9.10 g (91%)

^{1}H NMR (400 MHz, $CDCl_3$) δ^H ppm: 7.95 (2H, d, $^3J_{HH}$ = 8.6 Hz), 7.72 (2H, AB_2), 7.67 (1H, AB_2), 6.64 (2H, dd, $^3J_{HH}$ = 8.6 Hz, $^4J_{HH}$ = 2.4 Hz), 6.57 (2H, d, $^4J_{HH}$ = 2.4 Hz), 3.87 (6H, s), 3.86 (6H, s).

^{13}C{^{1}H} NMR (100.6 MHz, $CDCl_3$) δ^C ppm: 161.05, 158.18, 154.91, 135.16, 132.20, 122.56, 122.12, 104.99, 98.75, 55.53, 55.38.

2,4-[L]-OH: 2,6-*Bis*(2,4-dihydroxyphenyl)pyridine

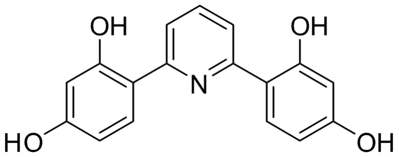

2,6-*Bis*(2,4-dimethoxyphenyl)pyridine (8.81 g, 25.07 mmol) was added to molten pyridinium chloride (37.28 g, 322.60 mmol) at 200 °C and stirred for 16 hr. The still molten mixture was carefully added to distilled water (200 mL) and the resulting bright yellow precipitate was isolated by filtration and air-dried. It was used without further purification. Yield: 10.55 g (143%, product heavily contaminated with water)

^{1}H NMR (400 MHz, DMSO-d$_6$) δ^H ppm: 10.37 (2H, br s), 8.23 (1H, t, $^3J_{HH}$ = 8.2 Hz), 7.96 (2H, d, $^3J_{HH}$ = 8.2 Hz), 7.71 (4H, d, $^3J_{HH}$ = 8.8 Hz), 6.55 (2H, d, $^4J_{HH}$ = 2.5 Hz), 6.48 (2H, dd, $^3J_{HH}$ = 7.2 Hz, $^4J_{HH}$ = 2.6 Hz).

2,4-[L]: 2,6-Bis(2,4-didodecyloxyphenyl)pyridine

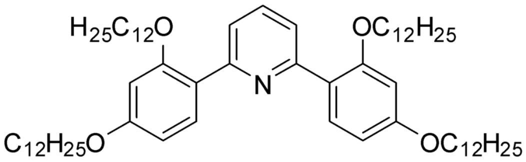

2,6-*Bis*(2,4-dihydroxyphenyl)pyridine (7.69 g, 26.04 mmol), 1-bromododecane (47.5 mL, 49.2 g, 167 mmol) and K_2CO_3 (57.66 g, 417.2 mmol) were heated to reflux in 3:1 2-butanone:methanol (500 mL) for 113 hr, then filtered and the filtrate reduced to dryness *in vacuo* to give an brown oil. The product was purified *via* column chromatography (silica, petroleum ether (40-60 °C (R_f = 0.00), then 7:3 CH_2Cl_2:petroleum ether (40-60 °C), (R_f = 0.15), then 5% ethyl acetate in petroleum ether (40-60 °C) (R_f = 0.36)) and a second column (silica, petroleum ether (40-60 °C (R_f = 0.00), then 7:3 CH_2Cl_2:petroleum ether (40-60 °C), (R_f = 0.15)), to give an orange oil, which solidified on standing to give an off-white solid. Yield: 6.64 g (26%)

^{1}H NMR (400 MHz, $CDCl_3$) δ^H ppm: 8.00 (2H, d $^3J_{HH}$ = 8.6 Hz), 7.79 (2H, d, $^3J_{HH}$ = 7.9 Hz), 7.63 (1H, t, $^3J_{HH}$ = 7.7 Hz), 7.62 (2H, dd, $^3J_{HH}$ = 8.6 Hz, $^4J_{HH}$ = 2.4 Hz), 6.55 (2H, d, $^4J_{HH}$ = 2.4 Hz), 4.02 (4H, t, $^3J_{HH}$ = 6.5 Hz), 4.01, (4H, t $^3J_{HH}$ = 6.6 Hz), 1.81 (8H, m), 1.46 (8H, m), 1.4 – 1.2 (64H, br s), 0.90 (6H, t, $^3J_{HH}$ = 7.1 Hz), 0.90 (6H, t, $^3J_{HH}$ = 7.0 Hz).

$^{13}C\{^1H\}$ NMR (100.6 MHz, $CDCl_3$) δ^C ppm: 160. 52, 157.79, 154.84, 134.81, 132.51, 122.42, 121.95, 105.58, 100.09, 68.47, 68.04, 31.91, 29.66, 29.63, 29.60, 29.58, 29.55, 29.46, 29.41, 29.35, 29.30, 29.28, 29.20, 26.14, 26.04, 22.67, 14.09.

2,5-[L]-OMe: 2,6-*Bis*(2,5-dimethoxyphenyl)pyridine

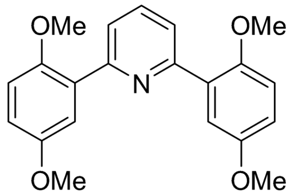

2,6-Dibromopyridine (6.74 g, 28.46 mmol) and 2,5-dimethoxyphenyl boronic acid (15.54 g, 85.37 mmol) were added to a flask containing [$Pd_2(OAc)_6$] (24.6 mg, 0.5 mol%) and K_3PO_4 (18.12 g, 85.37 mmol). Ethylene glycol (300 mL) was added, and the reaction mixture heated to 80 °C for 2 hr with vigorous stirring. The reaction mixture was cooled to room temperature, the solid precipitate isolated by filtration and washed with water (200 mL), after which it was air dried. The resulting grey solid was dissolved in warm CH_2Cl_2 (~100 mL) and filtered through Celite®. The filtrate was collected and the solvent removed under reduced pressure. The crystalline, off-white residue was crystallised from the minimum amount of hot ethanol to give the pure product as white needle-like crystals. Yield = 8.98 g (90%)

^{1}H NMR (400 MHz, $CDCl_3$) δ^H ppm: 7.81 (2H, AB_2), 7.73 (1H, AB_2), 7.56 (2H, d, $^4J_{HH}$ = 2.4 Hz), 6.93 (4H, m), 3.84 (6H, s), 3.83 (6H, s).

^{13}C{^{1}H} NMR (100.6 MHz, $CDCl_3$) δ^C ppm: 155.01, 153.90, 151.48, 135.40, 130.19, 123.21, 116.30, 115.19, 113.08, 56.43, 55.78.

2,5-[L]-OH: 2,6-*Bis*(2,5-dihydroxyphenyl)pyridine

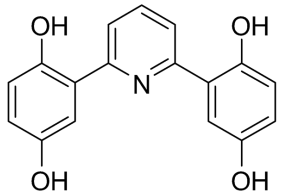

2,6-*Bis*(2,5-dimethoxyphenyl)pyridine (8.69 g, 24.73 mmol) was added to molten pyridinium chloride (30.79 g, 266.44 mmol) at 200 °C and stirred for 16 hr. The still molten mixture was carefully added to distilled water (200 mL) and the resulting bright yellow precipitate was isolated by filtration and air-dried. It was used without further purification. Yield: 8.48 g (116%, product contaminated with water)

^1H NMR (400 MHz, DMSO-D6) δ^H ppm: ~9.00 (2H, s br), 8.10 (1H, t, $^3J_{HH}$ = 8.2 Hz), 7.92 (2H, d, $^3J_{HH}$ = 8.0 Hz), 7.17 (2H, s), 6.83 (2H, d, $^3J_{HH}$ = 8.7 Hz), 6.78 (2H, d, $^3J_{HH}$ = 8.7 Hz).

2,5-[L]: 2,6-*Bis*(2,5-didodecyloxyphenyl)pyridine

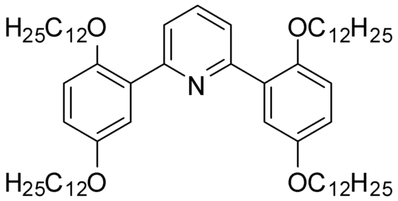

2,6-*Bis*(2,5-dihydroxyphenyl)pyridine (11.56 g, 39.15 mmol), 1-bromododecane (60.2 mL, 62.45 g, 250.56 mmol) and K_2CO_3 (86.71 g, 627.40 mmol) were heated to reflux in 2-butanone (300 mL) and methanol (100 mL) for 10 days, then cooled to room temperature and filtered. The resulting solid was washed with water (60 cm), and acetone. The remaining insoluble tan solid was crystallised from CH_2Cl_2/acetone twice to give an off-white microcrystalline solid. The crude filtrate was collected, reduced to dryness under reduced pressure and then extracted into CH_2Cl_2 and acetone added. The resulting solution was cooled at 5 °C, before being filtered and washed with acetone to isolate an off-white microcrystalline solid. Yield = 21.20 g (56%)

^1H NMR (400 MHz, $CDCl_3$) δ^H ppm: 7.86 (2H, AB_2), 7.67 (1H, AB_2), 7.59 (2H, d, $^4J_{HH}$ = 3.1 Hz), 6.92 (2H, d, $^3J_{HH}$ = 8.8 Hz), 6.87 (2H, dd, $^3J_{HH}$ = 8.8 Hz, $^4J_{HH}$ = 2.3 Hz), 3.97 (4H, t, $^3J_{HH}$ = 6.5 Hz), 3.94 (4H, t, $^3J_{HH}$ = 6.6 Hz), 1.75 (8H, m), 1.42 (8H, m), 1.27 (72H, br m), 0.87 (6H, t, $^3J_{HH}$ = 6.1 Hz), 0.87 (6H, t, $^3J_{HH}$ = 6.3 Hz).

$^{13}C\{^1H\}$ NMR (100.6 MHz, $CDCl_3$) δ^C ppm: 154.95, 153.46, 150.97, 134.99, 130.47, 123.14, 116.87, 115.92, 114.62, 69.73, 68.55, 31.91, 29.68, 29.66, 29.63. 29.61, 29.58, 29.47, 29.43, 29.35, 29.33, 26.13, 26.11, 22.67, 14.10.

2,5-[L]-C4: 2,6-*Bis*(2,5-butoxyphenyl)pyridine

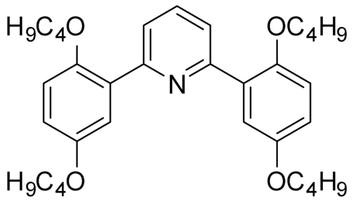

Sodium methoxide (0.95 g, 17.57 mmol) in methanol (30 mL) was added to 2,6-*bis*(2,5-dihydroxyphenyl)pyridine (1.26 g, 4.26 mmol) and 1-bromobutane (2.9 mL, 3.70 g, 27.01 mmol) in dry THF (100 mL) and was heated to reflux for 168 hr, with a further four equivalents of NaOMe (0.92 g, 1.7068 mmol) added over this time period. The reaction was cooled to room temperature and distilled water (50 mL) added, before the resulting solution was concentrated under reduced pressure. The remaining orange solution was extracted into CH_2Cl_2 (75 mL) and washed with distilled water (2 x 50 mL). The organic layer was dried over $MgSO_4$, filtered and reduced to dryness. The brown oil was purified by column chromatography (silica, petroleum ether (40 – 60 °C R_f = 0.00, then 95:5 petroleum ether (40-60 °C):ethyl acetate, R_f = 0.28) to give a buttercup yellow solid. Unfortunately the titular product and the trialkylated product eluted together in a 1:2 ratio by ^{1}H NMR. The mixed product, 1-bromododecane (0.25 mL, 0.32 g, 2.34 mmol) and K_2CO_3 (0.8071 g, 5.84 mmol) were heated to reflux in 2-butanone (75 mL) and methanol (25 mL) for 10 days, then cooled to room temperature and filtered. The pale yellow filtrate was reduced to dryness and purified *via* column chromatography (silica, petroleum ether (40 – 60 °C), R_f = 0.0, then 7:3 CH_2Cl_2:petroleum ether (40-60 °C):CH_2Cl_2, R_f = 0.59), then a second and third column (silica, :3 CH_2Cl_2:petroleum ether (40-60 °C): CH_2Cl_2, R_f = 0.59) to give an oil, which solidified on standing to give colourless crystals. Yield: 0.1506 g (7%)

^{1}H NMR (400 MHz, $CDCl_3$) δ^H ppm: 7.85 (2H, AB_2), 7.69 (1H, AB_2), 7.59 (2H, d, $^4J_{HH}$ = 3.0 Hz), 6.93 (2H, d, $^3J_{HH}$ = 9.0 Hz), 6.87 (2H, dd, $^3J_{HH}$ = 8.8 Hz, $^4J_{HH}$ = 3.1 Hz), 3.99 (4H, t, $^3J_{HH}$ = 6.4 Hz), 3.95 (4H, t, $^3J_{HH}$ = 6.5 Hz), 1.74 (8H, m), 1.45 (8H, m), 0.96 (6H, t, $^3J_{HH}$ = 7.3 Hz), 0.92 (6H, t, $^3J_{HH}$ = 7.4 Hz).

3,5-[L]-OMe: 2,6-*Bis*(3,5-dimethoxyphenyl)pyridine

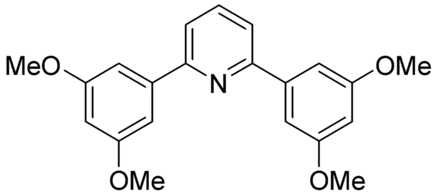

2,6-Dibromopyridine (6.74 g, 28.46 mmol) and 3,5-dimethoxyphenyl boronic acid (15.54 g, 85.37 mmol) were added to a flask containing $[Pd_2(OAc)_6]$ (21.1 mg, 0.5 mol%) and K_3PO_4 (18.12 g, 85.37 mmol). Ethylene glycol (400 mL) was added, and the reaction mixture heated to 80 °C for 2 hr with vigorous stirring. The reaction mixture was cooled to room temperature, the solid precipitate isolated by filtration and washed with water (150 mL), after which it was air dried. The resulting grey solid was dissolved in warm CH_2Cl_2 (~100 mL) and filtered through Celite®. The filtrate was collected and the solvent removed under reduced pressure. The crystalline, off-white residue was crystallised from the minimum amount of hot ethanol to give the pure product as white rhombic crystals. Yield = 8.92 g (89%)

^{1}H NMR (400 MHz, $CDCl_3$) δ^H ppm: 7.80 (1H, AB_2), 7.66 (2H, AB_2), 7.32 (4H, d, $^4J_{HH}$ = 2.3 Hz), 6.56 (2H, t, $^4J_{HH}$ = 2.3 Hz), 3.89 (12H, s).

^{13}C{^{1}H} NMR (100.6 MHz, $CDCl_3$) δ^C ppm: 161.03, 156.32, 141.53, 137.45, 119.11, 105.10, 101.08, 55.43.

3,5-[L]-OH: 2,6-*Bis*(3,5-dihydroxyphenyl)pyridine

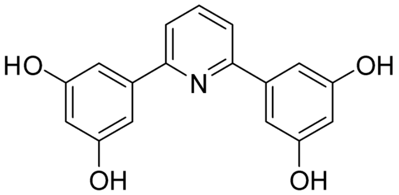

2,6-*Bis*(3,5-dimethoxyphenyl)pyridine (7.10 g, 20.20 mmol) was added to molten pyridinium chloride (29.47 g, 255.0 mmol) at 200 °C and stirred for 16 hr. The still molten mixture was carefully added to distilled water (200 mL) and the resulting red/pink precipitate was isolated by filtration and air-dried. It was used without further purification. Yield: 7.13 g (120%, product contaminated with water)

^{1}H NMR (400 MHz, DMSO-d$_6$) δ^H ppm: 9.40 (2H, br s), 7.89 (1H, t, $^3J_{HH}$ = 7.9 Hz), 7.70 (2H, d, $^3J_{HH}$ = 7.8 Hz), 7.01 (4H, d, $^4J_{HH}$ = 2.6 Hz), 6.31 (2H, t, $^4J_{HH}$ = 2.6 Hz).

3,5-[L]: 2,6-Bis(3,5-didodecyloxyphenyl)pyridine

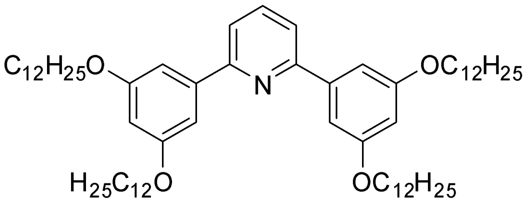

2,6-*Bis*(3,5-dihydroxyphenyl)pyridine (5.96 g, 20.18 mmol), 1-bromododecane (31 mL, 32.18 g, 125.11 mmol) and K_2CO_3 (48.81 g, 353.2 mmol) were heated to reflux in 2-butanone (300 mL) for 16 hr, then cooled to room temperature and filtered to isolate a brown filtrate, which was reduced to dryness. Methanol was added and the solution was cooled at 5 °C to give a brown solid, which was isolated by filtration and purified *via* column chromatography (silica, 95:5 petroleum ether (40-60 °C):ethyl acetate, R_f = 0.36) to give a pale orange oil, which solidified on standing to a pale pink crystalline solid. The solid was crystallised from hexane/ethyl acetate and a small amount of methanol to give off-white needle-like crystals, then from CH_2Cl_2/ethyl acetate to give white needle-like crystals. The residue was purified *via* column chromatography (silica, petroleum ether (40 – 60 °C), R_f = 0.0, then 9:1 petroleum ether (40-60 °C):ethyl acetate, R_f = 0.59), then crystallised from CH_2Cl_2/ethyl acetate to give off-white crystals. Yield = 5.25 g (27%)

^{1}H NMR (400 MHz, $CDCl_3$) δ^H ppm: 7.87 (1H, AB_2), 7.65 (2H, AB_2), 7.29 (4H, d, $^4J_{HH}$ = 2.3 Hz), 6.54 (2H, t, $^4J_{HH}$ = 2.3 Hz), 4.04 (8H, t, $^3J_{HH}$ = 6.5 Hz), 1.84 (8H, m), 1.48 (8H, m), 1.4-1.2 (64H, br m), 0.89 (12H, t, $^3J_{HH}$ = 6.8 Hz).

^{13}C{^{1}H} NMR (100.6 MHz, $CDCl_3$) δ^C ppm: 160.54, 156.42, 141.14, 137.30, 118.96, 105.56, 102.08, 68.12, 31.91, 31.89, 29.68, 29.64, 29.62, 29.60, 29.93, 29.35, 29.33, 26.09, 22.68, 14.11.

Mercury Complexes

Warning: *Mercury salts and organomercury complexes are toxic. Great care, as outlined in the Supplementary Information, should be taken in preparing and handling materials of this type. Furthermore, while an extensive study is reported here, evidently there are other confirmatory experiments that could have been carried out on some occasions. However, in order to reduce the handling of mercury, not all of these were attempted.*

4-[Hg]: [Mercury(II)(2,6-*Bis*(4-dodecyloxyphenyl)pyridine)chloride]

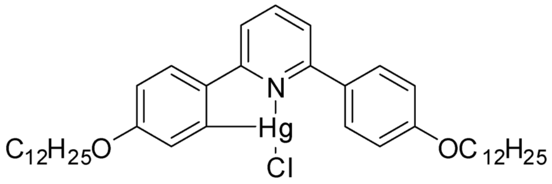

2,6-*Bis*(4-dodecyloxyphenyl)pyridine (7.00 g, 11.67 mmol) and $Hg(OAc)_2$ (7.40 g, 23.22 mmol) were added to ethanol (500 mL) and heated to vigorous reflux for 24 hr. The mixture was then cooled to 50 °C and a solution of LiCl (0.9893 g, 23.34 mmol) in methanol (50 mL) was added; the resulting mixture was allowed to stir for 15 min. Distilled water (200 mL) was added and the resulting solution cooled to room temperature, stirred overnight and filtered. The precipitate was washed with copious amounts of water and air-dried. The dried precipitate was dissolved in boiling CH_2Cl_2 (~200 mL) and filtered through Celite®, after which the solvent was removed from the filtrate under reduced pressure. The solid residue was crystallised from $CHCl_3$ and acetone, the resulting free ligand precipitate removed *via* filtration and the filtrate concentrated *in vacuo* to give a colourless solid, which was used without further purification. (1.87 g, 77% estimated [Hg(C^N^CH)Cl]).

^{1}H NMR (500 MHz, $CDCl_3$) δ^H ppm: 7.88 (1H, d, $^3J_{HH}$ = 8.7 Hz), 7.85 (2H, AA'XX', J = 8.8 Hz), 7.78 (1H, dd, $^3J_{HH}$ = 7.7 Hz, $^3J_{HH}$ = 7.7 Hz), 7.64 (1H, d, $^3J_{HH}$ = 7.9 Hz), 7.59 (1H, d, $^3J_{HH}$ = 7.9 Hz), 7.07 (1H, d, $^4J_{HH}$ = 2.7 Hz), 7.02 (2H, AA'XX', J = 8.8 Hz), 6.93 (1H, dd, $^3J_{HH}$ = 8.7 Hz, $^4J_{HH}$ = 2.6 Hz), 4.03 (2H, t, $^3J_{HH}$ = 6.6 Hz), 4.02 (2H, t, $^3J_{HH}$ = 6.6 Hz), 1.82 (4H, m), 1.48 (4H, m), 1.4-1.2 (32H, br m), 0.89 (6H, t, $^3J_{HH}$ = 7.1 Hz), 0.89 (6H, t, $^3J_{HH}$ = 7.1 Hz).

$^{13}C\{^1H\}$ NMR (125.8 MHz, $CDCl_3$) δ^C ppm: 160.27, 159.97, 157.66, 157.17, 149.83, 138.15, 135.65, 130.85, 129.05, 128.86, 123.23, 119.55, 118.35, 114.95, 130.85, 129.05, 128.86, 123.23, 119.55, 118.35, 114.95, 114.73, 68.29, 68.12, 31.91, 29.66, 29.63, 29.59, 29.57, 29.49, 29.38, 29.34, 29.26, 29.21, 26.05, 26.01, 22.68, 14.11.

^{199}Hg NMR (89.6 MHz, $CDCl_3$) δ^{Hg} ppm: -1023.43 (d, $^3J_{HHg}$ = 234.8 Hz)

2-[Hg]: [Mercury(II)(2,6-*Bis*(2-dodecyloxyphenyl)pyridine)chloride]

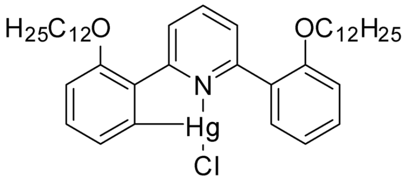

2,6-*Bis*(2-dodecyloxyphenyl)pyridine (12.39 g, 20.65 mmol) and $Hg(OAc)_2$ (13.53 g, 42.46 mmol) were added to ethanol (500 mL) and heated to vigorous reflux for 24 hr. The mixture was then cooled to 50 °C and a solution of LiCl (0.9893 g, 23.34 mmol) in methanol (50 mL) was added; the resulting mixture was allowed to stir for 15 min. Distilled water (200 mL) was added and the resulting solution cooled to room temperature and filtered. The precipitate (yellow solid/oil) was washed with copious amounts of water and a small amount of ethanol. The precipitate was dissolved in boiling $CHCl_3$ (~500 mL) and filtered through Celite®, after which the solvent was removed under reduced pressure to give the product as a very viscous pearlescent yellow oil. The residue was used without further purification. (~16 g, 86% estimated [Hg(C^N^CH)Cl]).

^1H NMR (400 MHz, $CDCl_3$) δ^H ppm: 8.13 (1H, dd, $^3J_{HH}$ = 7.9 Hz, $^4J_{HH}$ = 1.2 Hz), 7.82 (1H, dd, $^3J_{HH}$ = 7.8 Hz, $^4J_{HH}$ =1.1 Hz), 7.73 (1H, dd, $^3J_{HH}$ = 7.6 Hz, $^3J_{HH}$ = 7.6 Hz), 7.72 (1H, dd, $^3J_{HH}$ = 7.6 Hz, $^4J_{HH}$ = 2.0 Hz), 7.39 (1H, ddd, $^3J_{HH}$ = 8.2 Hz, $^3J_{HH}$ = 7.3 Hz, $^4J_{HH}$ = 1.8 Hz), 7.38 (1H, dd, $^3J_{HH}$ = 8.2 Hz, $^3J_{HH}$ = 7.1 Hz), 7.04 (4H, m), 4.06 (2H, t, $^3J_{HH}$ = 6.5 Hz), 4.03 (2H, t, $^3J_{HH}$ = 6.5 Hz), 1.83 (2H, m), 1.74 (2H, m), 1.47 (2H, m), 1.4-1.1 (34 H, broad m), 0.88 (3H, t, $^3J_{HH}$ = 7.1 Hz), 0.87 (3H, t, $^3J_{HH}$ = 7.1 Hz).

$^{13}C\{^1H\}$ NMR (125.8 MHz, $CDCl_3$) δ^C ppm: 158.28, 156.68, 155.83, 155.20, 153.53, 135.71, 132.22, 131.46, 130.13, 129.87, 129.55, 128.43, 124.16, 124.14, 120.86, 113.00, 112.65, 68.73, 68.56, 31.88, 29.65, 29.63, 29.61, 29.58, 29.54, 29.33, 29.28, 29.24, 29.22, 26.19, 26.17, 22.66, 14.10.

^{199}Hg NMR (89.6 MHz, $CDCl_3$) δ^{Hg} ppm: -1020 (dd, $^{3}J_{HHg}$ = 203.0 Hz, $^{4}J_{HHg}$ = 69.9 Hz).

R_f = 0.53 (CH_2Cl_2:petroleum ether (40 – 60 °C) 6:4)

3-[Hg]: [Mercury(II)(2,6-*Bis*(3-dodecyloxyphenyl)pyridine)chloride] and 5-[Hg]: [Mercury(II)(2,6-*Bis*(5-dodecyloxyphenyl)pyridine)chloride]

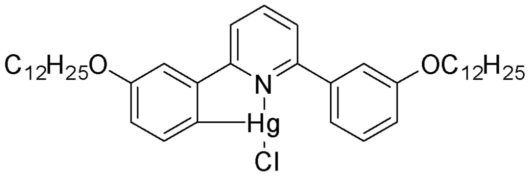

3-[Hg]

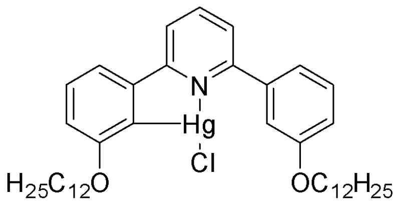

5-[Hg]

2,6-*Bis*(3-dodecyloxyphenyl)pyridine (7.00 g, 11.67 mmol) and $Hg(OAc)_2$ (7.41 g, 23.25 mmol) were added to ethanol (500 mL) and heated to a vigorous reflux for 24 hr. The reaction mixture was cooled to 50 °C and a solution of LiCl (0.9908 g, 23.37 mmol) in methanol (50 mL) was added; the resulting mixture was allowed to stir for 15 min. Distilled water (200 mL) was added and the resulting solution cooled to room temperature and filtered. The resulting sticky grey/green solid was washed with copious amounts of water, a small amount of ethanol and air-dried. Yield: 9.74 g (77% estimated [Hg(C^N^CH)Cl]) (82.8%)

The two isomers were present in a 1:0.72 ratio by ^{1}H NMR spectroscopy.

3-[Hg]:

^{1}H NMR (400 MHz, $CDCl_3$) δ^{H} ppm: 7.87 (1H, dd, $^{3}J_{HH}$ = 7.9 Hz, $^{3}J_{HH}$ = 7.9 Hz), 7.75 (1H, dd, $^{3}J_{HH}$ = 8.0 Hz, $^{4}J_{HH}$ = 0.9 Hz), 7.72 (1H, dd, $^{3}J_{HH}$ = 7.8 Hz, $^{4}J_{HH}$ = 1.0 Hz), 7.49 (1H, d, $^{3}J_{HH}$ = 2.6 Hz), 7.47 (1H, dd, $^{4}J_{HH}$ = 1.5 Hz, $^{4}J_{HH}$ = 1.5 Hz), 7.42 (3H, m), 7.02 (1H, ddd, $^{3}J_{HH}$ = 8.2 Hz, $^{4}J_{HH}$ = 2.4 Hz, $^{4}J_{HH}$ = 1.0 Hz), 7.02 (1H, d, $^{3}J_{HH}$ = 8.2 Hz), 4.07 (2H, t, $^{3}J_{HH}$ = 6.7 Hz), 4.05 (2H, t, $^{3}J_{HH}$ = 6.5 Hz), 1.802(4H, m), 1.49 (4H, m), 1.4 – 1.2 (32H, br m), 0.88 (3H, t, $^{3}J_{HH}$ = 6.9 Hz), 0.88 (3H, t, $^{3}J_{HH}$ = 6.8 Hz).

$^{13}C\{^1H\}$ NMR (100.6 MHz, $CDCl_3$) δ^C ppm: 159.78, 159.53, 157.91, 157.49, 144.72, 140.07, 139.63, 138.39, 138.34, 129.73, 121.10, 120.01, 119.92, 115.80, 115.09, 115.06, 113.58, 68.21, 68.13, 31.91, 29.66, 29.62, 29.59, 39.57, 29.45, 29.37, 29.34, 29.24, 26.08, 26.03, 22.67, 14.11.

^{199}Hg NMR (89.6 MHz, $CDCl_3$) δ^{Hg} ppm: -991.17 (d, $^3J_{HHg}$ = 190.8 Hz)

R_f = 0.44 (1:1 CH_2Cl_2:petroleum ether (40 – 60 °C)

5-[Hg]:

^1H NMR (400 MHz, $CDCl_3$) δ^H ppm: 7.86 (1H, dd, $^3J_{HH}$ = 7.8 Hz, $^3J_{HH}$ = 7.8 Hz), 7.78 (1H, dd, $^3J_{HH}$ = 8.0 Hz, $^4J_{HH}$ = 1.0 Hz), 7.71 (1H, dd, $^3J_{HH}$ = 7.6 Hz, $^4J_{HH}$ = 1.2 Hz), 7.58 (1H, d, $^3J_{HH}$ = 6.8 Hz), 7.51 (1H, ddd, $^3J_{HH}$ = 7.7 Hz, $^4J_{HH}$ = 1.6 Hz, $^4J_{HH}$ = 1.6 Hz), 7.44 (1H, dd, $^4J_{HH}$ = 2.6 Hz, $^4J_{HH}$ = 2.6 Hz), 7.42 (1H, dd, $^3J_{HH}$ = 7.8 Hz, $^3J_{HH}$ = 7.8 Hz), 7.41 (1H, dd, $^3J_{HH}$ = 7.8 Hz, $^3J_{HH}$ = 7.8 Hz), 7.02 (1H, ddd, $^3J_{HH}$ = 8.2 Hz, $^4J_{HH}$ = 2.8 Hz, $^4J_{HH}$ = 1.0 Hz), 7.00 (1H, dd, $^3J_{HH}$ = 7.2 Hz, $^4J_{HH}$ = 1.0Hz), 4.08 (2H, t, $^3J_{HH}$ = 6.5 Hz), 4.03 (2H, t, $^3J_{HH}$ = 6.4 Hz), 1.80 (4H, m), 1.47 (4H, m), 1.4 – 1.2 (32H, br m), 0.88 (3H, t, $^3J_{HH}$ = 6.9 Hz), 0.87 (3H, t, $^3J_{HH}$ = 6.9 Hz).

$^{13}C\{^1H\}$ NMR (100.6 MHz, $CDCl_3$) δ^C ppm: 161.69, 159.50, 157.91, 157.13, 144.40, 139.75, 138.21, 137.95, 130.57, 129.73, 121.02, 120.28, 120.04, 119.97, 115.67, 113.82, 112.06, 68.59, 68.12, 13.91, 29.67, 29.63, 29.61, 29.56, 29.45, 29.37, 29.35, 29.25, 26.08, 22.68, 14.11.

$^{199}Hg\{^1H\}$ NMR (89.6 MHz, $CDCl_3$) δ^{Hg} ppm: -993.39

R_f = 0.5 (4:6 CH_2Cl_2:petroleum ether (40 – 60 °C)), 0.56 (1:1 CH_2Cl_2:petroleum ether (40 – 60 °C), 0.72 (CH_2Cl_2:petroleum ether (40 – 60 °C) 6:4).

APCI MS (m/z): Expected for $C_{41}H_{60}ClHgNO_2$ = 835.40; Observed: 836.4126 $[M + H]^+$

3,4-[Hg]: [Mercury(II)(2,6-*Bis*(3,4-didodecyloxyphenyl)pyridine)chloride]

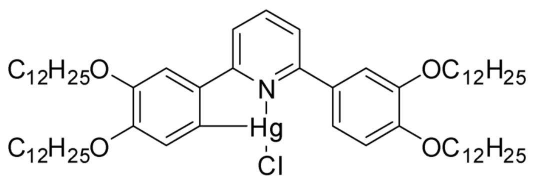

2,6-*Bis*(3,4-dodecyloxyphenyl)pyridine (0.50 g, 0.52 mmol) and $Hg(OAc)_2$ (0.60 g, 1.88 mmol) were added to ethanol (150 mL) and heated to vigorous reflux for 24 hr. The resulting solution was then cooled to 50 °C and a solution of LiCl (0.0803 g, 1.8941 mmol) in methanol (50 mL) was added. The resulting mixture was allowed to stir for 15 min. Distilled water (50 mL) was added and the resulting solution cooled to room temperature and filtered. The off-white precipitate was washed with copious amounts of water, a small amount of ethanol and air-dried. Yield: 0.46 g (37%)

^{199}Hg NMR (89.6 MHz, $CDCl_3$) δ^{Hg} ppm: -998.63

2,3-[Hg₂]: [Dimercury(II)(2,6-*Bis*(2,3-didodecyloxyphenyl)pyridine)dichloride]

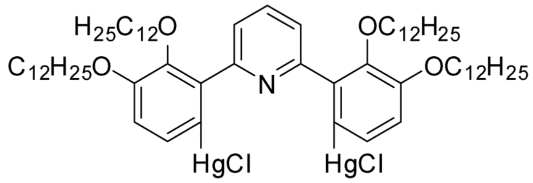

2,6-*Bis*(2,3-dodecyloxyphenyl)pyridine (4.02 g, 4.15 mmol) and $Hg(OAc)_2$ (2.92 g, 9.19 mmol) were added to ethanol (500 mL) and heated to vigorous reflux for 24 hr. The reaction mixture was cooled to 50 °C and a solution of LiCl (0.3526 g, 8.3172 mmol) in methanol (50 mL) was added; the resulting mixture was allowed to stir for 15 min. Distilled water (200 mL) was added and the resulting solution cooled to room temperature and filtered. The resulting white precipitate was filtered, washed with copious amounts of water and air-dried. Yield = 4.45 g (75%)

^{1}H NMR (400 MHz, $CDCl_3$) δ^{H} ppm: 8.12 (2H, d, $^{3}J_{HH}$ = 7.9 Hz), 7.79 (1H, AB_2), 7.11 (2H, d, $^{3}J_{HH}$ = 8.0 Hz), 7.01 (2H, AB_2), 4.05 (4H, t, $^{3}J_{HH}$ = 6.3 Hz), 3.90 (4H, broad m), 1.88 (4H, m), 1.55 (4H, m), 1.42-1.18 (72H, broad m), 0.88 (6H, t, $^{3}J_{HH}$ = 6.4 Hz), 0.87 (6H, t, $^{3}J_{HH}$ = 7.3 Hz).

$^{13}C\{^1H\}$ NMR (100.6 MHz, $CDCl_3$) δ^C ppm: 157.13, 153.19, 148.04, 137.35, 136.67, 132.38, 125.47, 113.93, 74.41, 68.60, 31.92, 31.90, 30.32, 29.71, 29.67, 29.65, 29.51, 29.43, 29.40, 29.37, 29.34, 26.18, 26.15, 22.68, 14.11.

^{199}Hg NMR (89.6 MHz, $CDCl_3$) δ^{Hg} ppm: -1012 (d, $^3J_{HHg}$ = 154.1 Hz)

ACPI MS: m/z = 968.8454 [ligand], 1204.7689 $[M+H]^+$, 1438.7045 $[M+H^+]$, 2334.5929 [Dimer]

Elemental Analysis: Found (%): C 53.5, H 7.4, N 1.2; Calc (%) for $C_{65}H_{107}Cl_2Hg_2NO_4$: C 54.3, H 7.5, N 1.0. Formally impure (further purification was not undertaken), but confirms assignment as dimercurated complex.

2,3-[Hg₂]-C4: [Dimercury(II)(2,6-*Bis*(2,3-dibutoxyphenyl)pyridine)dichloride]

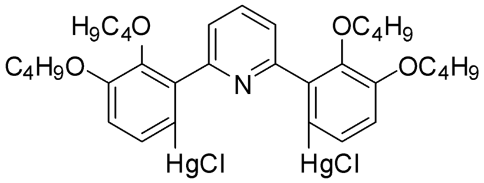

2,6-*Bis*(2,3-butoxyphenyl)pyridine (1.00 g, 1.92 mmol) and $Hg(OAc)_2$ (1.31 g, 4.11 mmol) were added to ethanol (200 mL) and heated to vigorous reflux for 24 hr. The resulting mixture was cooled to 50 °C and a solution of LiCl (0.19 g, 4.48 mmol) in methanol (50 mL) was added and the resulting mixture allowed to stir for 15 min. Distilled water (200 mL) was added and the resulting solution cooled to room temperature and filtered. The white precipitate was washed with copious amounts of water, a small amount of ethanol (<5 mL) and air-dried. Yield = 1.0443 g (55%)

^1H NMR (400 MHz, $CDCl_3$) δ^H ppm: 8.12 (2H, d, $^3J_{HH}$ = 7.9 Hz), 7.80 (1H, t, $^3J_{HH}$ = 7.9 Hz), 7.11 (2H, d, $^3J_{HH}$ = 8.1 Hz), 7.03 (2H, d, $^3J_{HH}$ = 8.1 Hz), 4.06 (4H, t, $^3J_{HH}$ = 6.5 Hz), .380 (4H, m), 1.87 (4H, m), 1.55 (8H, m), 1.34 (4H, m), 1.01 (6H, t, $^3J_{HH}$ = 7.3 Hz), 0.82 (6H, t, $^3J_{HH}$ = 7.3 Hz).

Elemental Analysis: Found (%): C 40.0 H 4.4, N 1.8; Calc (%) for $C_{33}H_{43}Cl_2Hg_2NO_4$: C 40.1, H 4.4, N 1.4.

2,4-[Hg]: [Mercury(II)(2,6-*Bis*(2,4-dibutyloxyphenyl)pyridine)chloride] and 2,4-[Hg_2]: [DiMercury(II)(2,6-*Bis*(2,4-dibutyloxyphenyl)pyridine)dichloride]

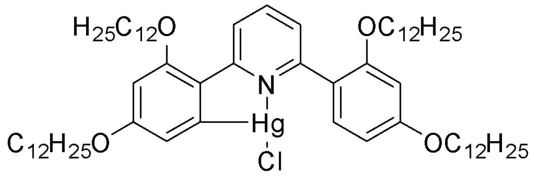

2,4-[Hg]

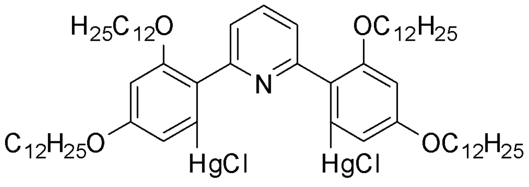

2,4-[Hg_2]

Using 2 molar equivalents of mercury

2,6-*Bis*(2,4-dodecyloxyphenyl)pyridine (1.70 g, 1.76 mmol) and $Hg(OAc)_2$ (1.18 g, 3.70 mmol) were added to ethanol (500 mL) and heated to a vigorous reflux for 24 hr. The reaction mixture was cooled to 50 °C and a solution of LiCl (0.1488 g, 3.51 mmol) in methanol (50 mL) was added; the resulting mixture was allowed to stir for 15 min. Distilled water (200 mL) was added and the resulting solution cooled to room temperature and filtered. The resulting caramel-like precipitate was washed with copious amounts of water and air-dried. Yield = 1.21 g (57%)

Highly complex ^{1}H NMR spectra. ~1:1 **2,4-[Hg]**: **2,4-[Hg_2]** estimated.

^{199}Hg NMR (89.6 MHz, $CDCl_3$) δ^{Hg} ppm: -1010.78, -1041.72

Using 1 molar equivalent of mercury

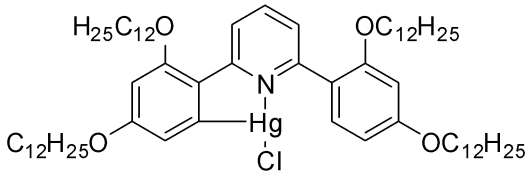

2,4-[Hg]

2,6-*Bis*(2,4-dodecyloxyphenyl)pyridine (4.19 g, 4.33 mmol) and $Hg(OAc)_2$ (1.48 g, 4.64 mmol) were added to ethanol (500 mL) and heated to a vigorous reflux for 24 hr. The reaction mixture was cooled to 50 °C and a solution of LiCl (0.3666 g, 8.65 mmol) in methanol (50 mL) was added; the resulting mixture was allowed to stir for 15 min. Distilled water (200 mL) was added and the

resulting solution cooled to room temperature and filtered. The resulting caramel-like precipitate was washed with copious amounts of water and air-dried. Yield = 1.86 g (36%)

^{1}H NMR (400 MHz, $CDCl_3$) δ^{H} ppm: 7.97 (1H, d, $^{3}J_{HH}$ = 8.6 Hz), 7.96 (1H, d, $^{4}J_{HH}$ = 2.2 Hz), 7.77 (1H, d, $^{3}J_{HH}$ = 7.8 Hz), 7.76 (1H, d, $^{3}J_{HH}$ = 7.8 Hz), 7.63 (1H, AB_2), 6.60 (1H, dd, $^{3}J_{HH}$ = 8.7 Hz, $^{4}J_{HH}$ = 2.3 Hz), 6.55 (1H, s), 6.53 (1H, d, $^{4}J_{HH}$ = 2.4 Hz), 4.00 (8H, m), 1.80 (8H, m), 1.45 (8H, m), 1.35 – 120 (52H, br m), 0.88 (12H, t, $^{3}J_{HH}$ = 6.8 Hz), 0.88 (12H, t, $^{3}J_{HH}$ = 7.1 Hz).

^{199}Hg NMR (89.6 MHz, $CDCl_3$) δ^{Hg} ppm: -1041.72.

2,5-[Hg]: [Mercury(II)(2,6-*Bis*(2,5-didodecyloxyphenyl)pyridine)chloride] and 2,5-[Hg_2]: [DiMercury(II)(2,6-*Bis*(2,5-didodecyloxyphenyl)pyridine)dichloride]

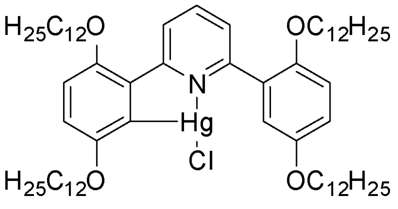

2,5-[Hg]

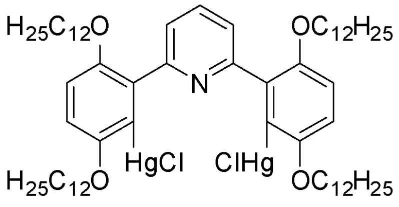

2,5-[Hg 2]

2,6-*Bis*(2,5-dodecyloxyphenyl)pyridine (3.00 g, 3.10 mmol) and $Hg(OAc)_2$ (2.06 g, 6.46 mmol) were added to ethanol (500 mL) and heated to a vigorous reflux for 24 hours. The resulting solution was cooled to 50 °C and a solution of LiCl (0.2630 g, 6.20 mmol) in methanol (50 mL) was added. The resulting mixture was allowed to stir for 15 min. Distilled water (200 mL) was added and the resulting solution cooled to room temperature and filtered. The waxy tan precipitate was washed with copious amounts of water and air-dried. Total Yield: 2.41 g

^{1}H NMR spectroscopy indicates that **2,5-[Hg]** and **2,5-[Hg_2]** were formed in a 5:4 ratio. Complexes can be separated *via* column chromatography (silica; 2:3 CH_2Cl_2: petroleum ether (40 – 60 °C), then 1:1 CH_2Cl_2: petroleum ether (40 – 60 °C)).

2,5-[Hg]

Yield: 1.24 g (33%)

^{1}H NMR (400 MHz, $CDCl_3$) δ^H ppm: 8.14 (1H, d, $^3J_{HH}$ = 8.0 Hz), 7.84 (1H, d, $^3J_{HH}$ = 7.6 Hz), 7.71 (1H, q, $^3J_{HH}$ = 8.1 Hz, $^3J_{HH}$ = 8.1 Hz), 7.29 (1H, d, $^4J_{HH}$ = 2.9 Hz), 6.94 (4H, s), 3.97 (8H, m), 1.75 (8H, m), 1.43 (8H, m), 1.35 – 1.15 (64H, m), 0.87 (12H, t, $^3J_{HH}$ = 6.8 Hz).

^{13}C{^{1}H} NMR (100.6 MHz, $CDCl_3$) δ^C ppm: 155.58, 155.16, 154.87, 153.60, 152.51, 150.87, 142.84, 135.67, 133.20, 129.43, 124.27, 124.05, 117.36, 116.39, 114.92, 114.77, 112.54, 69.75, 69.70, 69.15, 68.81, 31.89, 29.65, 29.61, 29.59, 29.55, 29.44, 29.41, 29.37, 29.33, 29.31, 26.20, 26.16, 26.14, 29.09, 26.04, 14.08.

R_f = 0.63 (1:1 CH_2Cl_2:petroleum ether (40 – 60 °C))

2,5-[Hg₂]

Yield: 1.17 g (26%)

^{1}H NMR (400 MHz, $CDCl_3$) δ^H ppm: 8.04 (1H, d, $^3J_{HH}$ = 7.9 Hz), 7.74 (1H, t, $^3J_{HH}$ = 8.1 Hz), 6.95-6.90 (4H, m), 3.95 (8H, t, $^3J_{HH}$ = 6.6 Hz), 1.75 (8H, m), 1.43 (8H, m), 1.35 – 1.15 (64H, m), 0.87 (12H, t, $^3J_{HH}$ = 6.8 Hz).

^{13}C{^{1}H} NMR (100.6 MHz, $CDCl_3$) δ^C ppm: 156.41, 155.31, 152.42, 143.26, 136.06, 133.33, 132.10, 132.00, 128.51, 128.39, 124.97, 116.08, 113.09, 70.46, 69.31, 31.87, 29.44, 29.41, 29.29, 29.27, 29.24, 29.28, 26.31, 14.08.

R_f = 0.69 (1:1 CH_2Cl_2:petroleum ether (40 – 60 °C))

2,5-[Hg]: [Mercury(II)(2,6-*Bis*(2,5-didodecyloxyphenyl)pyridine)chloride] and 2,5-[Hg_2]: [DiMercury(II)(2,6-*Bis*(2,5-didodecyloxyphenyl)pyridine)dichloride]

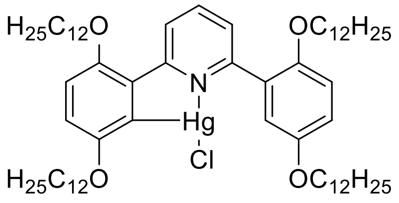

2,5-[Hg]

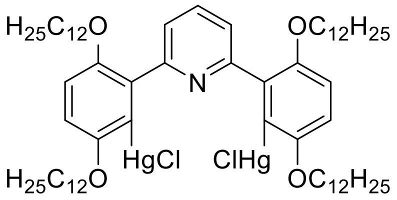

2,5-[Hg_2]

2,6-*Bis*(2,5-dodecyloxyphenyl)pyridine (6.00 g, 6.19 mmol) and $Hg(OAc)_2$ (4.20 g, 13.18 mmol) were added to ethanol (400 mL) and heated to a mild reflux for 24 hours. The resulting solution was cooled to 50 °C and a solution of LiCl (0.7824 g, 18.46 mmol) in methanol (50 mL) was added. The resulting mixture was allowed to stir for 15 min. Distilled water (200 mL) was added and the resulting solution cooled to room temperature and filtered. The waxy tan precipitate was washed with copious amounts of water and air-dried.

^{1}H NMR spectroscopy indicates that **2,5-[Hg]** and **2,5-[Hg_2]** were formed in a 4:1 ratio.

2,5-[Hg] Yield: 4.58 g (61.5 %)

2,5-[Hg_2] Yield: 1.32 g (14.8%)

2,5-[Hg]-C4: [Mercury(II)(2,6-*Bis*(2,5-dibutoxyphenyl)pyridine)chloride] and 2,5-[Hg_2]-C4: [DiMercury(II)(2,6-*Bis*(2,5-dibutoxyphenyl)pyridine)dichloride]

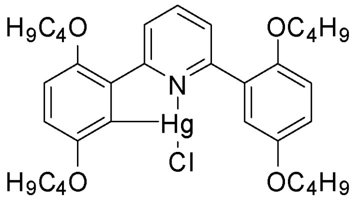

2,5-[Hg]-C4

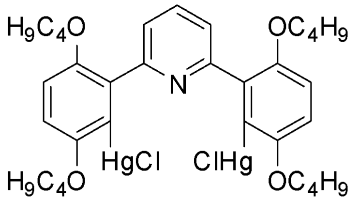

2,5-[Hg_2]-C4

2,6-*Bis*(2,5-butoxyphenyl)pyridine (0.13 g, 0.22 mmol) and $Hg(OAc)_2$ (0.17 g, 0.53 mmol) were added to ethanol (50 mL) and heated to a vigorous reflux for 24 hr. The solution was cooled to 55 °C and a solution of LiCl (0.0259 g, 0.6109 mmol) in methanol (10 mL) was added. The resulting mixture was allowed to stir for 15 min. Distilled water (50 mL) was added and the resulting solution cooled to room temperature and filtered. The viscous gold oil precipitate was washed with copious amounts of water and air-dried. ^{1}H NMR spectroscopy indicates that **2,5-[Hg]-C4** and

2,5-[Hg$_2$]-C4 were formed in a 4:3 ratio. The products were separated by column chromatography (silica, 6:4 CH_2Cl_2:petroleum ether (40 – 60 °C)) to give **2,5-[Hg]-C4** as an oil, which solidified on standing to give colourless crystals and **2,5-[Hg$_2$]-C4** as an oil, which solidified on standing to give a white solid.

2,5-[Hg]-C4

Yield: 0.0516 g (43%)

^{1}H NMR (400 MHz, $CDCl_3$) δ^H ppm: 8.11 (1H, dd, $^3J_{HH}$ = 7.5 Hz, $^4J_{HH}$ = 0.6 Hz), 7.84 (1H, dd, $^3J_{HH}$ = 7.6 Hz, $^4J_{HH}$ = 1.1 Hz), 7.72 (1H, dd, $^3J_{HH}$ = 7.9 Hz, $^3J_{HH}$ = 7.9 Hz), 7.29 (1H, d, $^4J_{HH}$ = 2.8 Hz), 6.92 (4H, m), 3.98 (1H, t, $^3J_{HH}$ = 6.6 Hz), 3.97 (1H, t, $^3J_{HH}$ = 6.4 Hz), 3.94 (1H, t, $^3J_{HH}$ = 6.6 Hz), 3.94 (1H, t, $^3J_{HH}$ = 6.4 Hz), 1.74 (6H, m), 1.67 (2H, m), 1.47 (6H, m), 1.36 (2H, m), 0.94 (9H, t, $^3J_{HH}$ = 7.4 Hz), 0.90 (3H, t, $^3J_{HH}$ = 7.4 Hz).

^{13}C{^{1}H} NMR (100.6 MHz, $CDCl_3$) δ^C ppm: 155.57, 155.15, 154.95, 153.61, 152.48, 150.88,142.80,135.75, 133.31, 129.44, 124.30, 124.08, 117.35, 116.46, 115.00, 114.79, 112.56, 69.47, 69.43, 68.84, 68.54, 31.48, 31.43, 31.39, 19.39, 19.31, 19.23, 13.90, 13.82, 13.79.

R_f = 0.38 (6:4 CH_2Cl_2:petroleum ether (40 – 60 °C))

^{199}Hg{^{1}H} NMR (89.6 MHz, $CDCl_3$) δ^{Hg} ppm: -1008.68

2,5-[Hg$_2$]-C4

Yield: 0.0802 g (38%)

^{1}H NMR (400 MHz, $CDCl_3$) δ^H ppm: 8.03 (1H, d, $^3J_{HH}$ = 7.9 Hz), 7.74 (1H, t, $^3J_{HH}$ = 7.7 Hz), 6.94 (4H, s), 3.95 (4H, t, $^3J_{HH}$ = 6.5 Hz), 3.95 (4H, t, $^3J_{HH}$ = 6.5 Hz), 1.72 (8H, m), 1.44 (8H, m), 0.95 (6H, t, $^3J_{HH}$ = 7.4 Hz), 0.90 (6H, t, $^3J_{HH}$ = 7.4 Hz).

$^{13}C\{^1H\}$ NMR (100.6 MHz, $CDCl_3$) δ^C ppm: 156.37, 155.27, 152.39, 143.19, 136.15, 133.27, 124.95,1 16.01, 113.07, 70.08, 68.97, 31.39, 31.35, 19.31, 13.84, 13.79.

$^{199}Hg\{^1H\}$ NMR (89.6 MHz, $CDCl_3$) δ^{Hg} ppm: -1010.38

R_f = 0.46 (6:4 CH_2Cl_2:petroleum ether (40 – 60 °C))

3,5-[Hg]: [Mercury(II)(2,6-*Bis*(3,5-didodecyloxyphenyl)pyridine)chloride] and 3,5-[Hg$_2$]]: [DiMercury(II)(2,6-*Bis*(3,5-dibutyloxyphenyl)pyridine)dichloride]

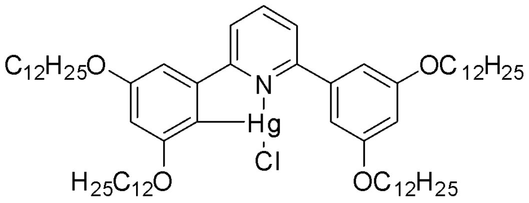

3,5-[Hg]

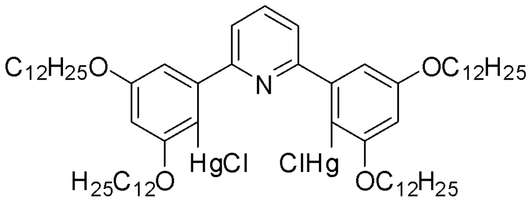

3,5-[Hg 2]

2,6-*Bis*(3,5-dodecyloxyphenyl)pyridine (3.07 g, 3.17 mmol) and $Hg(OAc)_2$ (1.98 g, 6.21 mmol) were added to ethanol (500 mL) and heated to a vigorous reflux for 24 hr. The resulting solution was cooled to 50 °C and a solution of LiCl (0.2635 g, 6.22 mmol) in methanol (50 mL) was added. The resulting mixture was allowed to stir for 15 min. Distilled water (200 mL) was added and the resulting solution cooled to room temperature and filtered. The resulting golden viscous oil was washed with copious amounts of water and air-dried. Yield: **3,5-[Hg]** = 0.35 g (9%), **3,5-[Hg$_2$]** = 0.21 g (3%)

1:0.5 **3,5-[Hg]**:**3,5-[Hg$_2$]** given by ^1H NMR.

^1H NMR (500 MHz, $CDCl_3$) δ^H ppm: 7.86 (0.5(1)H, t, $^3J_{HH}$ = 7.9 Hz), 7.85 (1H, d, $^3J_{HH}$ = 7.7 Hz), 7.81 (1H, d, $^3J_{HH}$ = 7.9 Hz), 7.72 (1(2)H, d, $^3J_{HH}$ = 7.6 Hz), 7.69 (1H, dd, $^3J_{HH}$ = 7.7 Hz, $^3J_{HH}$ =7.7 Hz), 7.04 (1.8 H, m), 6.97 (1H, d, $^4J_{HH}$ = 2.4 Hz), 6.59 (2.8H, m), 4.1 – 4.0 (12H, m), 1.47 (12H, m), 1.4-1.2 (104H, m), 0.89 (18H, m).

$^{13}C\{^1H\}$ NMR (100.6 MHz, $CDCl_3$) δ^C ppm: 162.68, 162.53, 161.49, 160.50, 158.78, 157.76, 157.13, 144.82, 144.14, 140.15, 138.68, 138,08, 131.30, 129.79, 121.53, 121.07, 119.98, 106.35, 105.79, 102.43, 100.40, 100.04, 68.79, 68.53, 68.33, 68.27, 68.14, 31.89, 29.65, 29.61, 29.59, 29.57, 29.54, 29.43, 29.41, 29.39, 29.33, 29.30, 29.27, 26.16, 26.06, 26.04, 22.65, 14.08.

$^{199}Hg\{^1H\}$ NMR (89.6 MHz, $CDCl_3$) δ^{Hg} ppm: -971.65, -954.42.

NMR data are for inseparable mixture and therefore non-integral ^1H NMR integrations reflect this. Data given in brackets reflect the 'true' integration values for the dimercury complex pyridyl system.

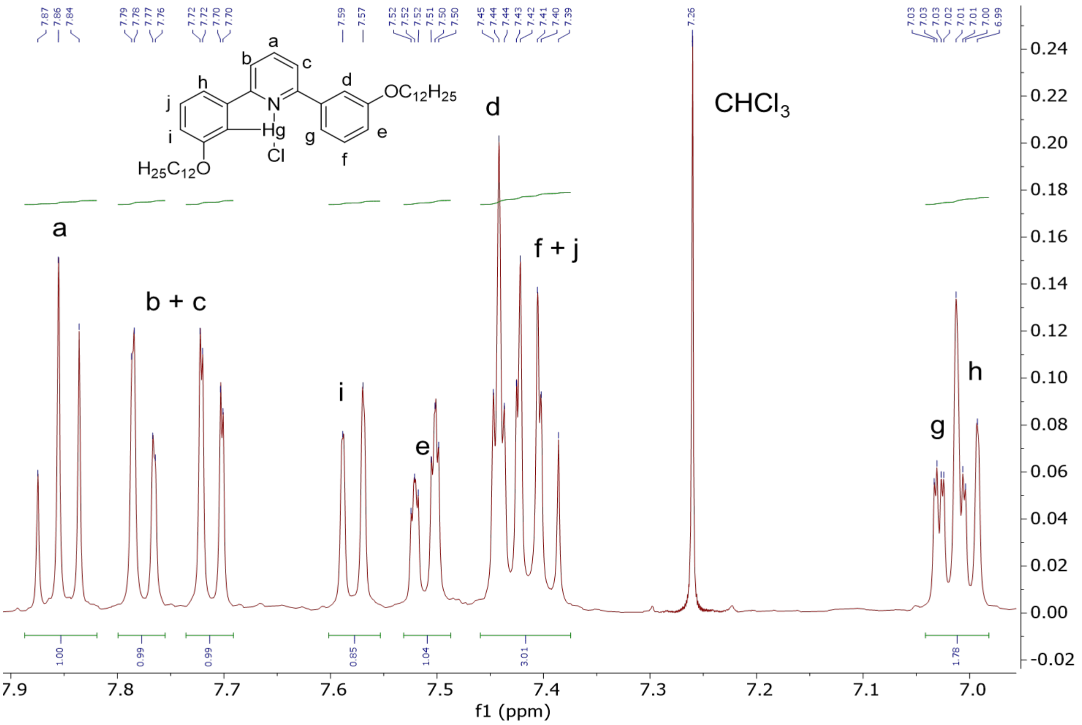

Figure S1 ^{1}H NMR (400 MHz, CDCl$_3$) of the 5-position isomer of Compound **5-[Hg]**, showing the aromatic region.

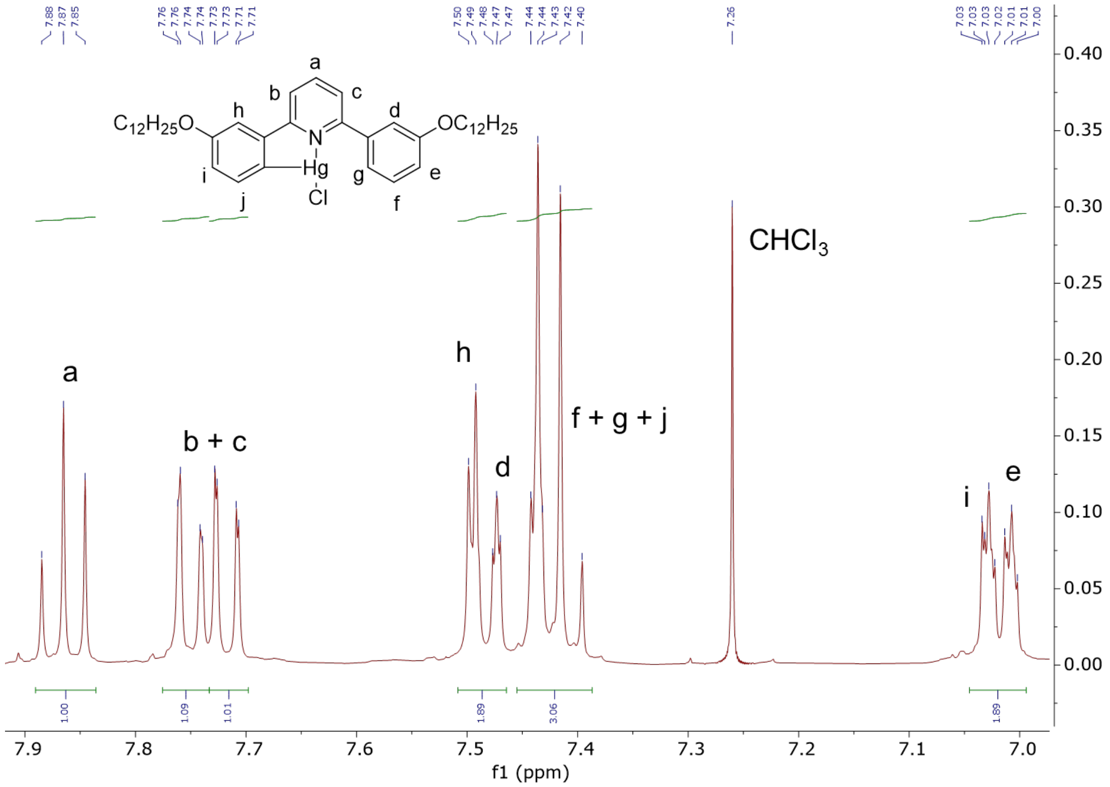

Figure S2 ^{1}H NMR (400 MHz, CDCl$_3$) of the 3-position isomer Compound **3-[Hg]**, showing the aromatic region.

Gold Complexes

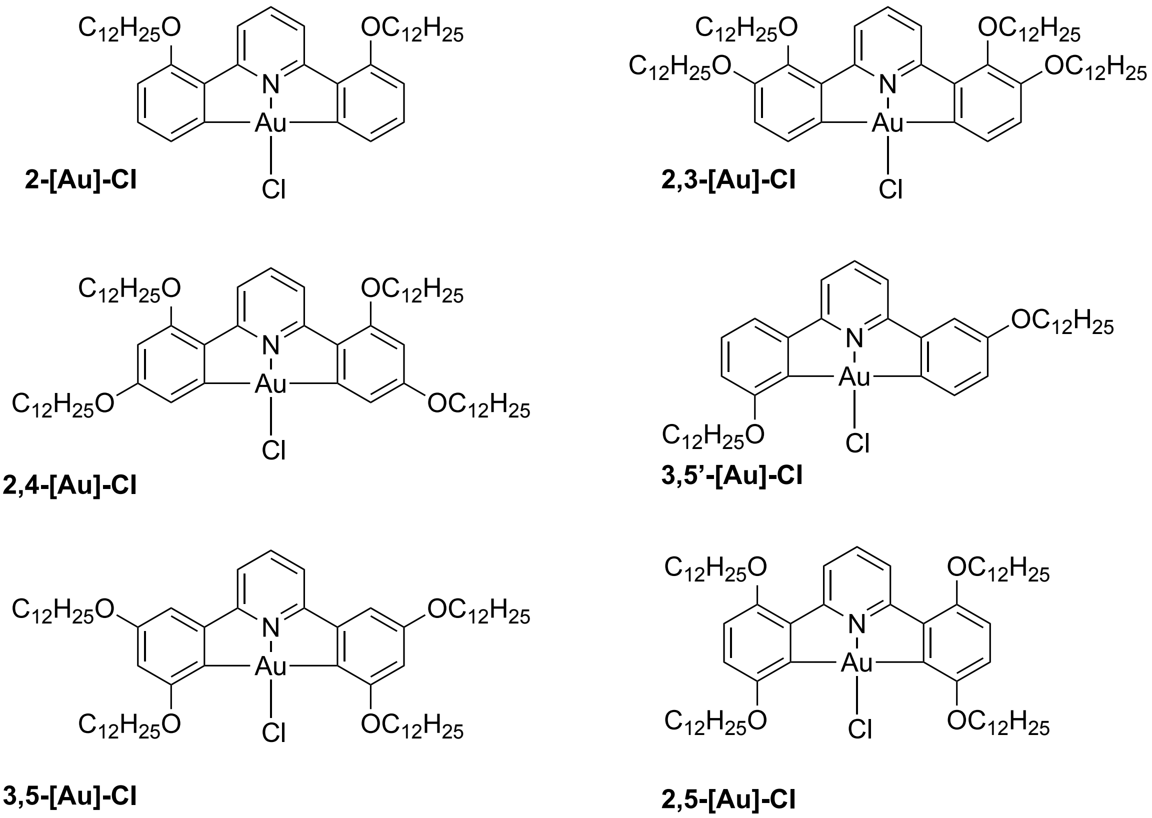

Figure S3 Numbering system for the chlorogold(III) complexes.

2-[Au]-Cl: [Gold(III)(2,6-*Bis*(2-dodecyloxyphenyl)pyridine)chloride]

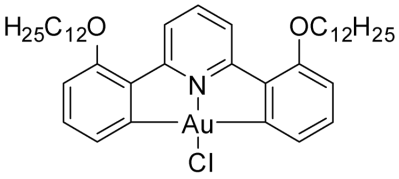

K[$AuCl_4$] (1.3211 g, 3.4971 mmol) and the mixed **2-[Hg]** product (2.92 g, 86.0% estimated **2-[Hg]**) were heated to reflux in acetonitrile (400 mL) under a dinitrogen atmosphere for 24 hr. Then the mixture was cooled to room temperature, distilled water (100 mL) added and filtered to isolate the product as a dirty yellow solid, which was purified *via* column chromatography (silica, CH_2Cl_2:petroleum ether (40 – 60 °C) 6:4, R_f = 0.71) to give a bright yellow crystalline solid, which was crystallised from CH_2Cl_2 and ethyl acetate to give yellow, needle-like crystals. Yield: 1.1463 g (40%)

^{1}H NMR (400 MHz, $CDCl_3$) δ^H ppm: 8.19 (2H, d, $^3J_{HH}$ = 8.5 Hz), 7.74 (1H, t, $^3J_{HH}$ = 8.2 Hz), 7.51 (2H, dd, $^3J_{HH}$ = 7.2 Hz, $^4J_{HH}$ = 1.0 Hz), 7.34 (2H, dd, $^3J_{HH}$ = 8.3 Hz, $^3J_{HH}$ = 7.2 H), 6.72 (2H, dd, $^3J_{HH}$ = 8.4 Hz, $^4J_{HH}$ = 0.9 Hz), 4.05 (4H, t, $^3J_{HH}$ = 6.6 Hz), 1.90 (4H, quint, $^3J_{HH}$ = 7.1 Hz), 1.51 (4H, m), 1.30 (32H, m), 0.88 (6H, t, $^3J_{HH}$ = 6.7 Hz)

$^{13}C\{^1H\}$ NMR (125.8 MHz, $CDCl_3$) δ^C ppm: 170.92, 164.14, 157.71, 142.42, 134.79, 132.09, 125.25, 121.62, 110.73, 68.36, 31.92, 29.67, 29.66, 29.61, 29.59, 29.40, 29.36, 29.13, 26.19, 22.69, 14.11.

APCI MS (m/z): Expected for $C_{41}H_{59}AuClNO_2$ = 830.39727; Observed: 830.400227 $[M + H]^+$ (Error = 2.9 mDa)

Elemental Analysis: Found (%): C 58.8, H 7.1, N 1.7; Calc (%) for $C_{41}H_{59}AuClNO_2$: C 59.3, H 7.2, N 1.7.

3/5-[Au]-Cl:[Gold(III)((2-(3-dodecyloxyphenyl)-6-(5'-dodecyloxyphenyl))pyridine)chloride]

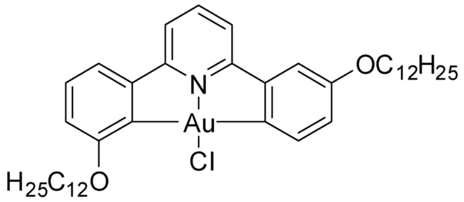

Na[$AuCl_4$] (1.1805 g, 2.9676 mmol) and the mixed **3-[Hg]** and **5-[Hg]** product (3.04 g, 77.4% estimated [Hg(C^N^CH)Cl])) were heated to reflux in acetonitrile (400 mL) under a dinitrogen atmosphere for 24 hr. The reaction mixture was cooled to room temperature and the resulting dirty yellow/white precipitate isolated by filtration and washed with ethanol. The product was dissolved in CH_2Cl_2 (74 mL) and filtered through Celite® to remove solid gold(0). After reduction to dryness the crude product was purified *via* column chromatography (silica, CH_2Cl_2:petroleum ether (40 – 60 °C) 4:6, then CH_2Cl_2:petroleum ether (40 – 60 °C) 6:4) , followed by a second column to give two products: a pale yellow crystalline solid, which on recrystallisation from CH_2Cl_2/ethyl acetate gave a white crystalline solid identified as **5-[Hg]**, and a yellow crystalline solid, identified as **3/5-[Au]-Cl**.

3/5-[Au]-Cl was purified further *via* column chromatography (silica, then CH_2Cl_2:petroleum ether (40 – 60 °C) 6:4, R_f = 0.5) twice, then crystallised from CH_2Cl_2/ethyl acetate to give a yellow microcrystalline solid. Yield: 0.4964 g (20%)

^{1}H NMR (400 MHz, $CDCl_3$) δ^H ppm: 7.76 (1H, dd, $^3J_{HH}$ = 7.9 Hz, $^3J_{HH}$ = 7.9 Hz), 7.70 (1H, d, $^3J_{HH}$ = 9.8 Hz), 7.34 (1H, dd, $^3J_{HH}$ = 8.2 Hz, $^4J_{HH}$ = 1.2 Hz), 7.29 (1H, dd, $^3J_{HH}$ = 8.1 Hz, $^4J_{HH}$ = 1.0 Hz), 7.10 (1H, dd, $^3J_{HH}$ = 7.7 Hz, $^3J_{HH}$ = 7.7 Hz), 7.05 (1H, dd, $^3J_{HH}$ = 7.8 Hz, $^4J_{HH}$ = 1.5 Hz), 6.90 (1H, dd, $^3J_{HH}$ = 7.9 Hz, $^4J_{HH}$ = 1.4 Hz), 6.85 (2H, m), 3.93 (2H, t, $^3J_{HH}$ = 6.6 Hz), 3.85 (2H, t, $^3J_{HH}$ = 6.4 Hz), 1.92 (2H, m), 1.73 (2H, m), 1.4 – 1.2 (32H, br m), 0.88 (3H, t, $^3J_{HH}$ = 6.9 Hz), 0.87 (3H, t, $^3J_{HH}$ = 4.8 Hz).

^{13}C{^{1}H} NMR (100.6 MHz, $CDCl_3$) δ^C ppm: 165.27, 164.08, 163.26, 159.25, 158.63, 158.46, 148,59, 147.71, 142.40, 132.88, 128.16, 117.93, 117.51, 117.27, 116.75, 116.26, 111.14, 69.14, 67.96, 31.93, 31.92, 29.72, 29.69, 29.65, 29.63, 29.49, 29.38, 29.36, 29.29, 29.23, 26.32, 26.02, 22.69, 14.12.

R_f = 0.07 (CH_2Cl_2:petroleum ether (40 – 60 °C) 4:6), 0.5 (6:4 CH_2Cl_2:petroleum ether (40 – 60 °C)).

APCI MS (m/z): Expected for $C_{41}H_{59}AuClNO_2$ = 830.3973; Observed: 830.3982 $[M + H]^+$ (Error = -0.9 mDa)

Elemental Analysis: Found (%): C 58.7, H 6.9, N 1.3; Calc (%) for $C_{54}H_{74}AuNO_2$: 59.3, H 7.2, N 1.7. (Carbon is 0.6% out.)

2,3-[Au]-Cl: [Gold(III)(2,6-*Bis*(2,3-didodecyloxyphenyl)pyridine)chloride]

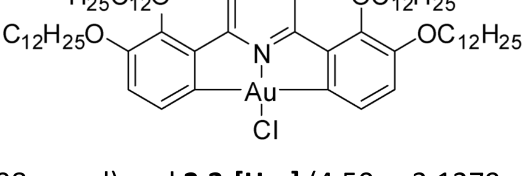

Na[$AuCl_4$] (1.4678 g, 3.6898 mmol) and **2,3-[Hg_2]** (4.50 g, 3.1279 mmol) were heated to reflux in 1:1 chloroform:acetonitrile (400 mL) under nitrogen for 24 hr. The reaction mixture was cooled to room temperature and reduced to dryness under reduced pressure, then extracted into

acetonitrile (50 mL) and the insoluble solid isolated by filtration. The brown solid was washed sequentially with distilled water (50 mL) and ethanol (25 mL), dried *in vacuo* and crystallised from CH_2Cl_2. The resulting dirty yellow solid was isolated by filtration, washed with ethyl acetate and then redissolved in CH_2Cl_2 and a hot filtrate was done to give a yellow solid on solvent removal, which was crystallised from chloroform/hexane to give the titular product as a microcrystalline yellow solid. Yield: 1.041 g (24%)

^{1}H NMR (400 MHz, $CDCl_3$) δ^H ppm: 8.31 (2H, d, $^3J_{HH}$ = 8.3 Hz), 7.84 (1H, t, $^3J_{HH}$ = 8.4 Hz), 7.55 (2H, d, $^3J_{HH}$ = 8.4 Hz), 7.01 (2H, d, $^3J_{HH}$ = 7.9 Hz), 4.08 (4H, t, $^3J_{HH}$ = 6.8 Hz), 3.98 (4H, t, $^3J_{HH}$ = 6.4 Hz), 1.83 (8H, m), 1.54-1.19 (72H, broad m), 0.88 (6H, t, $^3J_{HH}$ = 5.9 Hz), 0.88 (6H, t, $^3J_{HH}$ = 6.5 Hz).

^{13}C{^{1}H} NMR (125.8 MHz, $CDCl_3$) δ^C ppm: 164.45, 160.42, 151.62, 148.31, 142.72, 140.14, 128.31, 122.08, 116.53, 73.40, 68.86, 31.92, 30.37, 29.70, 29.66, 29.65, 29.55, 29.45, 29.37, 26.23, 26.10, 22.69, 14.11.

APCI MS (m/z): Expected for $C_{78}H_{122}AuNO_4$ = 1198.7627; Observed: 1198.7565 $[M + H]^+$ (Error = -5.2 mDa)

Elemental Analysis: Found (%): C 65.0, H 8.8, N 0.9; Calc (%) for $C_{65}H_{107}AuClNO_4$: C 65.1, H 9.0, N 1.2.

2,3-[Au]-Cl-C4: [Gold(III)(2,6-*Bis*(2,3-dibutoxyphenyl)pyridine)chloride]

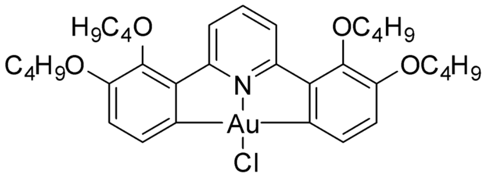

K[$AuCl_4$] (0.3433 g, 0.9085 mmol) and **2,3-[Hg_2]-C4** (0.90 g, 0.90 mmol) were heated to reflux in 1:1 chloroform:acetonitrile (200 mL) under a dinitrogen atmosphere for 24 hr. The reaction mixture was cooled to room temperature and reduced to dryness under reduced pressure, then extracted into acetonitrile (50 mL) and the insoluble solid isolated by filtration. The dirty tan solid was washed sequentially with distilled water (50 mL) and ethanol (25 mL) and dried *in vacuo*. It was dissolved in CH_2Cl_2 and filtered through Celite® to remove elemental gold. The resulting clear

bright yellow solution was reduced to dryness under reduced pressure and the yellow, needle-like crystals crystallised from CH_2Cl_2/hexane. The yellow solid was purified *via* column chromatography (silica; then 6:4 CH_2Cl_2: petroleum ether (40 – 60 °C) (R_f = 0.65)) and crystallised from CH_2Cl_2/ethyl acetate to give bright yellow, long needle-like crystals. Yield: 0.3094 g (47%)

^{1}H NMR (400 MHz, $CDCl_3$) δ^H ppm: 8.30 (2H, d, $^3J_{HH}$ = 8.2 Hz), 7.84 (1H, t, $^3J_{HH}$ = 8.2 Hz), 7.54 (2H, d, $^3J_{HH}$ = 8.0 Hz), 7.01 (2H, d, $^3J_{HH}$ = 8.2 Hz), 4.10 (4H, t, $^3J_{HH}$ = 6.9 Hz), 3.99 (4H, t, $^3J_{HH}$ = 6.4 Hz), 1.82 (8H, m), 1.00 (6H, t, $^3J_{HH}$ = 7.5 Hz), 1.00 (6H, t, $^3J_{HH}$ = 7.3 Hz).

2,4-[Au]-Cl: [Gold(III)(2,6-*Bis*(2,4-didodecyloxyphenyl)pyridine)chloride

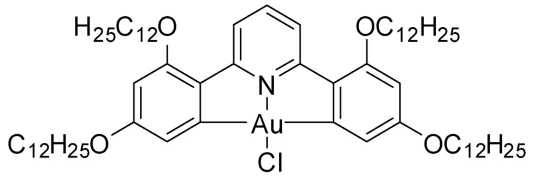

Na[$AuCl_4$] (1.2818 g, 3.2222 mmol) and the mixed **2,4-[Hg]** product (3.89 g, 3.23 mmol) were heated in 1:1 chloroform:acetonitrile (600 mL) under a dinitrogen atmosphere at reflux for 24 hr. The solution was cooled to room temperature and reduced to dryness to give a brown solid, which was extracted into acetonitrile (50 mL). The insoluble solid was isolated by filtration and extracted into CH_2Cl_2 (~50 mL), then filtered to remove insoluble Au(0) and the filtrate reduced to dryness to give a brown solid, which was purified *via* column chromatography (silica; 1:1 CH_2Cl_2: petroleum ether (40 – 60 °C) (R_f = 0.53)) twice to give a yellow solid. The solid was crystallised from CH_2Cl_2/ethyl acetate to give bright yellow small needles. The product was purified *via* column chromatography (silica; 4:6 CH_2Cl_2: petroleum ether (40 – 60 °C) (R_f = 0.41)) to give a yellow solid, which was crystallised from chloroform/ethyl acetate to give bright yellow small needles. Yield: 0.0849 g (5%)

^{1}H NMR (400 MHz, $CDCl_3$) δ^H ppm: 7.93 (2H, d, $^3J_{HH}$ = 8.1 Hz), 7.60 (2H, t, $^3J_{HH}$ = 8.0 Hz), 7.13 (2H, d, $^4J_{HH}$ = 2.9 Hz), 6.24 (2H, d, $^4J_{HH}$ = 2.2 Hz), 4.07 (4H, t, $^3J_{HH}$ = 6.3 Hz), 4.01 (4H, t, $^3J_{HH}$ = 6.9 Hz), 1.88 (8H, m) 1.78 (8H, m), 1.5-1.2 (72H, br m), 0.88 (6H, t, $^3J_{HH}$ = 6.6 Hz), 0.88 (6H, t, $^3J_{HH}$ = 6.6 Hz).

$^{13}C\{^1H\}$ NMR (125.8 MHz, $CDCl_3$) δ^C ppm: 172.69, 164.05, 162.30, 159.43, 142.08, 127.40, 118.77, 110.02, 98.50, 68.40, 68.14, 31.96, 31.92, 29.79, 29.75, 29.70, 29.68, 29.66, 29.64, 29.61, 29.59, 29.48, 29.43, 29.38, 29.39, 29.08, 26.16, 26.07, 22.73, 22.69, 14.12.

APCI MS (m/z): Expected for $C_{78}H_{122}AuNO_4$ = 1198.7627; Observed: 1198.7588 $[M + H]^+$ (Error = 3.9 mDa)

2,5-[Au]-Cl: [Gold(III)(2,6-*bis*(2,5-didodecyloxyphenyl)pyridine)chloride]

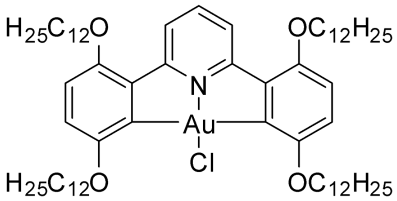

Na[$AuCl_4$] (0.7971 g, 2.00 mmol) and the mixed **2,5-[Hg]** product (2.41 g, 2.00 mmol) were heated in 1:1 chloroform:acetonitrile (200 mL) under a dinitrogen atmosphere at reflux for 24 hr. The reaction mixture was cooled to room temperature and reduced to dryness under reduced pressure, then extracted into acetonitrile (50 mL) and the insoluble solid isolated by filtration. The brown solid was washed sequentially with distilled water (25 mL) and ethanol (25 mL), dried *in vacuo*, dissolved in CH_2Cl_2 and filtered to remove solid gold(0). The filtrate was reduced to dryness under reduced pressure and triturated with diethyl ether three times to solidify to a brown solid, which was crystallised three times from CH_2Cl_2/ethyl acetate to give a bright yellow microcrystalline solid. Yield: 0.4071 g (17%)

^1H NMR (400 MHz, $CDCl_3$) δ^H ppm: 8.39 (2H, d, $^3J_{HH}$ = 82 Hz), 7.75 (2H, t, $^3J_{HH}$ = 8.4 Hz), 6.98 (2H, d, $^3J_{HH}$ = 9.1 Hz), 6.73 (2H, d, $^3J_{HH}$ = 9.2 Hz), 4.04 (4H, t, $^3J_{HH}$ = 6.6 Hz), 3.94 (4H,), 0.88 (6H, t, $^3J_{HH}$ = 7.0 Hz), t, $^3J_{HH}$ = 6.8 Hz), 1.88 (8H, m) 1.50 (8H, m), 0.88 (6H, t, $^3J_{HH}$ = 7.0 Hz).

$^{13}C\{^1H\}$ NMR (100.6 MHz, $CDCl_3$) δ^C ppm: 164.13, 157.52, 157.08, 152.83, 141.57, 135.56, 122.04, 121.45, 112.03, 72.14, 68.19, 31.93, 31.90, 29.74, 29.69, 29.66, 29.64, 29.58, 29.56, 29.38, 29.34, 29.23, 26.29, 29.23, 22.69, 22.67, 14.11.

APCI MS (m/z): Expected for $C_{65}H_{107}AuClNO_4$ = 1198.7627; Observed: 1198.7588 $[M + H]^+$ (Error = -3.9 mDa)

Elemental Analysis: Found (%): C 65.0, H 8.8, N 0.9; Calc (%) for $C_{65}H_{107}AuClNO_4$: C 65.1, H 9.0, N 1.2.

Attempted Synthesis of [Gold(III)(2,6-bis(3,5-didodecyloxyphenyl)pyridine)chloride (3,5-[Au]-Cl)

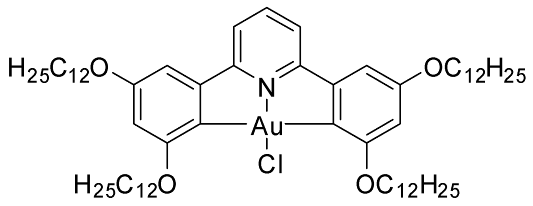

Na[$AuCl_4$] (0.1849 g, 0.46 mmol) and the mixed **3,5-[Hg]** product (0.56 g, 0.46 mmol) were heated to reflux in 1:1 chloroform:acetonitrile (200 mL) under a dinitrogen atmosphere for 24 hr. The reaction mixture was cooled to room temperature and reduced to dryness under reduced pressure, then the soluble salts extracted into acetonitrile (10 mL) to give a remaining viscous golden oil, which was determined by ^{1}H NMR spectroscopy to be the unreacted mercury(II) complexes.

Attempted Direct Auration

(Bu_4N)[$AuCl_4$]

H[$AuCl_4$] (0.4958 g, 1.4591 mmol) was dissolved in distilled water (25 mL) and solid (Bu_4N)Cl (0.4113 g, 1.4799 mmol) was added and stirred at room temperature for 3 hr in the dark. The resulting yellow precipitate was isolated by vacuum filtration, washed with distilled water (10 mL) and air dried. Yield = 0.680 g (80%)

^{1}H NMR (400 MHz, $CDCl_3$) δ^H ppm: 3.45 (8H, m), 1.83 (8H, m), 1.44 (8H, m), 0.98 (12H, t, $^3J_{HH}$ = 4.6 Hz).

Elemental Analysis: Found (%): C 33.0, H 6.1, N 2.2; Calc (%) for $C_{16}H_{36}AuCl_4N$: C 33.1, H 6.2, N 2.4.

Attempted Direct Auration using K[AuCl$_4$]

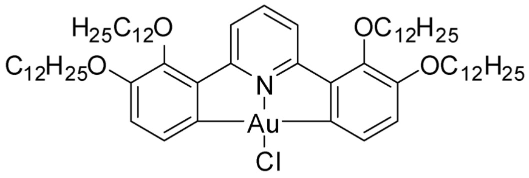

Attempt 1

K[AuCl$_4$] (0.1532 g, 0.4054 mmol) and 2,6-*bis*(2,3-didodecyloxyphenyl)pyridine (0.1532 g, 0.1582 mmol) in 1:1 chloroform:acetonitrile (200 mL) were heated to reflux under a dinitrogen atmosphere for 24 hr, then cooled to room temperature and reduced to dryness under reduced pressure. The residue was extracted into acetonitrile (25 mL) and the insoluble solid filtered off and washed with water (25 mL), then ethanol (15 mL) to give a pale orange solid, which was purified *via* column chromatography (silica, 95:5 petroleum ether (40 – 60 °C):ethyl acetate, then CH_2Cl_2).

APCI MS: m/z = 968.8414 $[M + H]^+$ (free ligand)

^{1}H NMR spectroscopy indicated only free ligand present.

Attempt 2

2,6-*Bis*(2,3-didodecyloxyphenyl)pyridine (0.1456 g, 0.1511 mmol) and K[AuCl$_4$] (0.0784 g, 0.2075 mmol) in 1:1 chloroform:acetonitrile (50 mL) were heated to reflux under a dinitrogen atmosphere for 5 days, then cooled to room temperature and reduced to dryness under reduced pressure. The residue was extracted into acetonitrile (25 mL) and the insoluble solid filtered off and washed with water (25 mL), then ethanol (15 mL) to give a pale orange solid.

^{1}H NMR spectroscopy indicated only free ligand present.

Auration *via* Palladium Complex Intermediate

2,3-[Pd$_2$]

2,6-*Bis*(2,3-didodecylphenyl)pyridine (0.5004 g, 0.5166 mmol) and K$_2$[PdCl$_4$] (0.1125 g, 0.3446 mmol) were heated to reflux in ethanol (50 mL) for 24 hr. The solution was cooled to room

temperature and the resulting yellow precipitate was isolated *via* filtration. The solid was washed by distilled water (15 mL), then ethanol (2.5 mL) and air dried. A second crop was isolated *via* the same method. The combined product was purified by column chromatography (silica, 95:5 petroleum ether (40 – 60 °C):ethyl acetate, R_f = 0.03, then 7:3 CH_2Cl_2: petroleum ether (40 – 60 °C), R_f = 0.47) to give a yellow oil, which solidified on standing to give a golden glassy solid. Yield: 0.1188 g (31%)

^{1}H NMR (400 MHz, $CDCl_3$) δ^H ppm: 8.48 (2H, d, $^3J_{HH}$ = 8.3 Hz), 7.89 (2H, t, $^3J_{HH}$ = 8.6 Hz), 7.46 (2H, t, $^3J_{HH}$ = 7.6 Hz), 7.38 (2H, d, $^3J_{HH}$ = 8.6 Hz), 7.16 (2H, dd, $^3J_{HH}$ = 7.4 Hz, $^3J_{HH}$ = 7.4 Hz), 7.04 (2H, d, $^3J_{HH}$ = 7.4 Hz), 6.97 (2H,d, $^3J_{HH}$ = 7.4 Hz), 6.66 (2H, d, $^3J_{HH}$ = 8.7 Hz), 4.07 (2H, br s), 4.00 (2H, br s), 3.92 (2H, t, $^3J_{HH}$ = 6.3 Hz), 1.80 (16H, m), 1.46 (16H, m), 1.4 – 1.0 (136H, br m), 0.86 (24H, m).

MALDI-TOF MS (m/z): Expected for $C_{130}H_{216}ClN_2O_8Pd_2$ = 2180.430948; Observed: 2180.435632 [M + H]$^+$ (Error = -0.2 mDa).

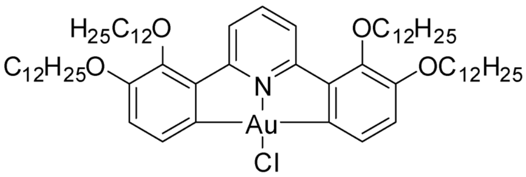

2,3-[Au]-Cl

2,3-[Pd₂] (0.0995, 0.0448 mmol) and Na[$AuCl_4$] (0.0365 g, 0.0918 mmol) were heated to reflux in 1:1 chloroform:acetonitrile (50 mL) for 24 hr under a dinitrogen atmosphere. The solution was cooled to room temperature and reduced to dryness under reduced pressure. The off-white to purple solid was extracted into acetonitrile (15 mL) and the insoluble solid isolated *via* filtration, washed with acetonitrile (10 mL) and air dried. The purple solid was dissolved in CH_2Cl_2 and filtered through Celite® to remove elemental gold and the resulting yellow solution reduced to dryness. The remaining mustard yellow solid was recrystallised from CH_2Cl_2/hexane to give a pale yellow solid. Yield: 0.0467 g (48%)

^1H NMR (400 MHz, $CDCl_3$) δ^H ppm: 8.31 (2H, d, $^3J_{HH}$ = 8.1 Hz), 7.84 (1H, t, $^3J_{HH}$ = 8.5 Hz), 7.55 (2H, d, $^3J_{HH}$ = 7.9 Hz), 7.01 (2H, d, $^3J_{HH}$ = 9.1 Hz), 4.08 (4H, t, $^3J_{HH}$ = 7.0 Hz), 3.98 (4H, t, $^3J_{HH}$ = 6.8 Hz), 1.83 (8H, m), 1.47 (8H, m), 1.4-1.1 (68H, br m), 0.88 (6H, t, $^3J_{HH}$ = 6.9 Hz), 0.88 (6H, t, $^3J_{HH}$ = 6.9 Hz).

APCI MS (m/z): Expected for $C_{78}H_{122}AuNO_4$ = 1198.7627; Observed: 1198.7599 $[M + H]^+$ (Error = -2.8 mDa)

2-[Pd$_2$]

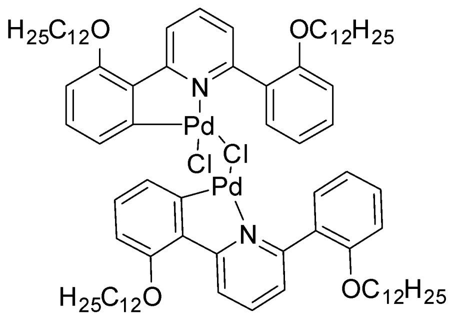

2,6-*Bis*(2-dodecylphenyl)pyridine (0.5014 g, 0.8358 mmol) and $K_2[PdCl_4]$ (0.1818 g, 0.5570 mmol) were heated to reflux in ethanol (50 mL) for 24 hr. The solution was cooled to room temperature and the resulting precipitate was isolated *via* filtration. The white solid was washed by distilled water (25 mL), then ethanol (2.5 mL) and air dried and shown by ^1H NMR spectroscopy to be the free ligand. A second crop of pale yellow crystals was isolated *via* the same method and was shown by ^1H NMR spectroscopy to be the impure product. The product was purified by column chromatography (silica, 95:5 petroleum ether (40 – 60 °C):ethyl acetate, R_f = 0.03, then 95:5 CH_2Cl_2: methanol, R_f = 0.82) to give a yellow oil, which solidified on standing to give a golden glassy solid. Yield: 0.0575 g (31%)

^1H NMR (400 MHz, $CDCl_3$) δ^H ppm: 8.46 (2H, dd, $^3J_{HH}$ = 8.3 Hz, $^4J_{HH}$ = 1.2 Hz), 7.89 (2H, dd, $^3J_{HH}$ = 8.2 Hz, $^3J_{HH}$ = 8.2 Hz), 7.47 (2H, ddd, $^3J_{HH}$ = 8.8 Hz, $^3J_{HH}$ = 7.3 Hz, $^4J_{HH}$ = 1.3 Hz), 7.40 (6H, m), 7.20 (4H, m), 6.95 (2H, dd, $^3J_{HH}$ = 8.2 Hz, $^3J_{HH}$ = 8.2 Hz), 6.66 (2H, dd, $^3J_{HH}$ = 7.4 Hz, $^4J_{HH}$ = 1.0 Hz), 4.56 (4H, t, $^3J_{HH}$ = 6.4 Hz), 4.08 (4H, t, $^3J_{HH}$ = 6.6 Hz), 1.90 (8H, m), 1.50 (4H, m), 1.39 (4H, m), 1.3 – 1.1 (64 H br m), 0.88 (6H, t, $^3J_{HH}$ = 6.8 Hz), 0.87 (6H, t, $^3J_{HH}$ = 7.2 Hz).

MALDI-TOF MS (m/z): Expected for $C_{82}H_{120}ClN_2O_4Pd_2$ = 1443.701559; Observed: 1443.701559 [M + H]$^+$ (Error = 1.7 mDa)

2-[Au]-Cl

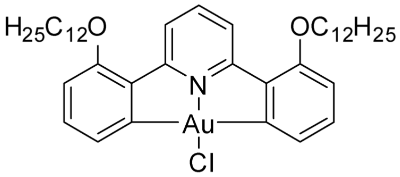

2-[Pd$_2$] (0.0531, 0.0358 mmol) and Na[AuCl$_4$] (0.0289 g, 0.0726 mmol) were heated to reflux in 1:1 chloroform:acetonitrile (50 mL) for 24 hr under a dinitrogen atmosphere. The solution was cooled to room temperature and reduced to dryness under reduced pressure. The off-white to purple solid was extracted into acetonitrile (20 mL) and the insoluble solid isolated *via* filtration, washed with distilled water (10 mL), then acetonitrile (5 mL) and air dried to give small yellow needles. Yield: 0.0177 g (30%)

^{1}H NMR (400 MHz, $CDCl_3$) δ^H ppm: 8.15 (2H, d, $^3J_{HH}$ = 8.2 Hz), 7.70 (1H, t, $^3J_{HH}$ = 8.2 Hz), 7.46 (2H, dd, $^3J_{HH}$ = 7.2 Hz, $^4J_{HH}$ = 1.0 Hz), 7.30 (2H, dd, $^3J_{HH}$ = 8.2 Hz, $^3J_{HH}$ = 7.2 Hz), 6.67 (2H, dd, $^3J_{HH}$ = 8.5 Hz, $^4J_{HH}$ = 1.0 Hz), 4.01 (4H, t, $^3J_{HH}$ = 6.9 Hz), 1.88 (4H, m), 1.50 (4H, m), 1.3-1.2 (32H, m), 0.88 (6H, t, $^3J_{HH}$ = 6.8 Hz).

APCI MS (m/z): Expected for $C_{41}H_{59}AuClNO_2$ = 830.39727; Observed: 830.3973 [M + H]$^+$ (Error = 4.7 mDa)

Auration *via* Rhodium Catalysis

[Cp*RhCl$_2$]$_2$

$RhCl_3.3H_2O$ (2.5022 g, 9.5047 mmol) and 1,2,3,4,5-pentamethylcyclopentadiene (3.0 mL, 2.61 g, 19.26 mmol) were heated to reflux in methanol (100 mL) for 72 hours under a dinitrogen atmosphere. The resulting solution was cooled to room temperature and the brick-red precipitate isolated *via* filtration. The filtrate was concentrated to ~25 mL and the brick-red precipitate

isolated *via* filtration. The combined solid was washed with diethyl ether (3 x 50 mL) and dried to give a brick-red solid. A third crop of red solid was isolated from the filtrate. Yield: 2.0606 g (35%)

^{1}H NMR (400 MHz, $CDCl_3$) δ^H ppm: 1.62 (15H, s).

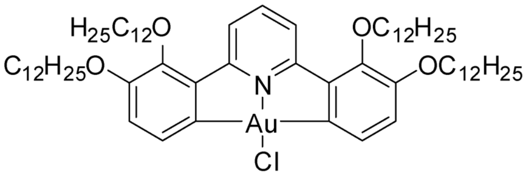

2,3-[Au]-Cl

$Na[AuCl_4]$ (0.1024 g, 0.2574 mmol), NaOBz (0.0447 g, 0.3102 mml) and $[Cp^*RhCl_2]_2$ (4.14 mg, 6.70 µmol) were added to a solution of 2,6-*bis*(2,3-didodecylphenyl)pyridine (0.2951 g, 0.3047 mmol) in ethyl acetate (20 mL) and heated at 60 °C for 19 hr. The resulting cloudy straw coloured solution was cooled to room temperature and the light brown precipitate isolated *via* filtration and washed with distilled water (20 mL), ethanol (10 mL) and air dried. The solid was dissolved in chloroform and filtered through a Celite® plug to remove elemental gold and the resulting yellow solution reduced to dryness to give a pale yellow microcrystalline solid. Yield: 0.0316 g (11%)

^{1}H NMR (400 MHz, $CDCl_3$) δ^H ppm: 8.31 (2H, d, $^3J_{HH}$ = 8.2 Hz), 7.84 (1H, t, $^3J_{HH}$ = 8.4 Hz), 7.55 (2H, d, $^3J_{HH}$ = 8.0 Hz), 7.01 (2H, d, $^3J_{HH}$ = 8.0 Hz), 4.08 (4H, t, $^3J_{HH}$ = 6.9 Hz), 3.98 (4H, t, $^3J_{HH}$ = 6.4 Hz), 1.83 (8H, m), 1.47 (8H, m), 1.4-1.1 (68H, br m), 0.88 (6H, t, $^3J_{HH}$ = 6.8 Hz), 0.88 (6H, t, $^3J_{HH}$ = 6.8 Hz).

APCI MS (m/z): Expected for $C_{78}H_{122}AuNO_4$ = 1198.7627; Observed: 1198.7627 $[M + H]^+$ (Error = 6.7 mDa).

(Unsuccessful) Attempted Direct Auration using $(Bu_4N)[AuCl_4]$

Attempt 1

2,6-*Bis*(2,3-didodecyloxyphenyl)pyridine (0.4039 g, 0.4170 mmol) and $(Bu_4N)[AuCl_4]$ (0.2428 g, 0.4054 mmol) in 1:1 chloroform:acetonitrile (200 mL) were heated to reflux under a dinitrogen

atmosphere for 5 hr, then cooled to room temperature and reduced to dryness under reduced pressure. The residue was extracted into acetonitrile (25 mL) and the insoluble solid filtered off and washed with ethanol (10 mL) to give a pale orange solid.

^{1}H NMR spectroscopy indicated only free ligand present.

Attempt 2

2,6-*Bis*(2,3-didodecyloxyphenyl)pyridine (0.1456 g, 0.1503 mmol) and $(Bu_4N)[AuCl_4]$ (0.1215 g, 0.2090 mmol) in 1:1 chloroform:acetonitrile (50 mL) were heated to reflux under a dinitrogen atmosphere for 5 days, then cooled to room temperature and reduced to dryness under reduced pressure. The residue was extracted into acetonitrile (25 mL) and the insoluble solid filtered off and washed with water (25 mL), then ethanol (15 mL) to give an off-white solid.

^{1}H NMR spectroscopy indicated only free ligand present.

Attempt 3

2,6-*Bis*(2,3-didodecyloxyphenyl)pyridine (0.2022 g, 0.2085 mmol), $(Bu_4N)[AuCl_4]$ (0.1212 g, 0.2085 mmol) and $(Bu_4N)(AcO)$ (0.0629 g, 0.2086 mmol) in acetone (50 mL) were heated to reflux under a dinitrogen atmosphere for 7 days, then cooled to room temperature and reduced to dryness under reduced pressure. The residue was extracted into acetonitrile (25 mL) and the insoluble solid filtered off and washed with acetonitrile to give an off-white solid.

^{1}H NMR spectroscopy indicated only free ligand present.

Attempted Direct Auration using $H[AuCl_4]$

Attempt 1

2,6-*Bis*(2,3-didodecyloxyphenyl)pyridine (0.1464 g, 0.1511 mmol) and $H[AuCl_4]$ (0.0712 g, 0.2095 mmol) in 1:1 chloroform:acetonitrile (50 mL) were heated to reflux under a dinitrogen atmosphere for 5 days, then cooled to room temperature and reduced to dryness under reduced pressure. The residue was extracted into acetonitrile (25 mL) and the insoluble solid filtered off and washed with

water (25 mL), then ethanol (15 mL) to give a pale orange solid. ^{1}H NMR spectroscopy indicated only free ligand present.

Attempted Direct Auration *via* Lithium Species

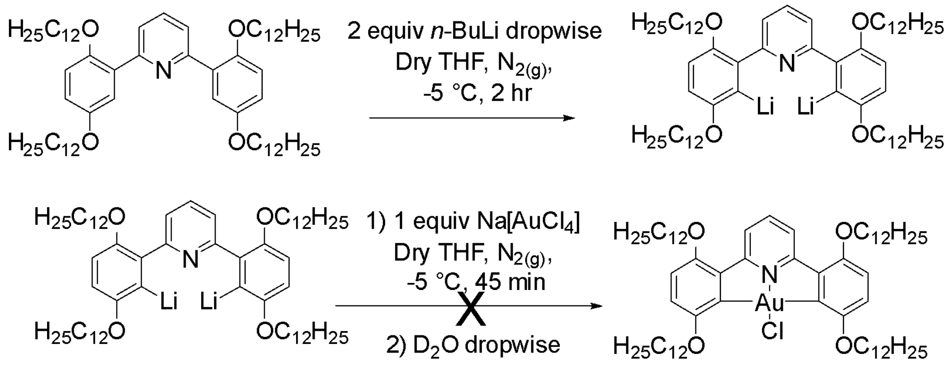

2,6-*Bis*(2,3-didodecyloxyphenyl)pyridine (0.1001 g, 0.1033 mmol) in dry THF (10 mL) was cooled in an ice/salt bath. *n*-BuLi (0.13 mL, 0.21 mmol, 1.6 M in hexanes) was added dropwise and the resulting solution stirred at <–5 °C for 2 hr under a dinitrogen atmosphere to give a dark brown solution. $Na[AuCl_4]$ (0.0362 g, 0.0910 mmol) in dry THF (5 mL) was cooled in an ice/salt bath. The dilithium species solution was added dropwise and stirred at <-5 °C for 45 min under a dinitrogen atmosphere. The reaction was quenched by the addition of D_2O (10 mL) resulting in a dark solution, which was extracted into CH_2Cl_2 950 mL). The organic layer was washed with distilled water (25 mL), then saturated brine (25 mL), before it was dried over $MgSO_4$, filtered and reduced to dryness to give an off-white solid. ^{1}H NMR indicated that only free ligand was present. Yield: 0.0 g (0%)

X-ray Structures of Ligands

Single crystals, suitable for X-ray diffraction, were grown of the new ligands and two of their tetrahydroxy precursors (labelled as ***x*-[L]-OH**). They were grown using vapour diffusion of an anti-solvent into a solution of the compound in a solvent.

2,3-[L]-OH

2,3-[L]-OH crystallised from ethyl acetate and hexane as yellow needles in the monoclinic space group ($P2_1/c$) (Fig. S4). One of the phenyl rings is almost coplanar (8.49°) with the plane of the pyridine ring, driven by an internal hydrogen bond between one hydroxyl group and the pyridyl nitrogen ($d_{N\text{-}H}$ = 2.537(1) Å). The plane of the other phenyl group makes an angle of 44.64° with that of the pyridyl ring.

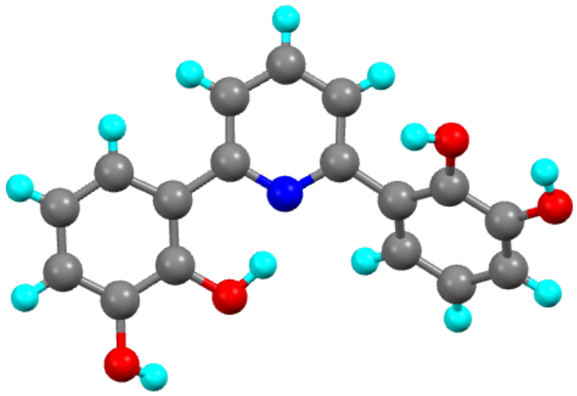

Figure S4 Structure of **2,3-[L]-OH**.

2,3-[L]

2,3-[L] crystallised from CH_2Cl_2 and ethanol in an orthorhombic space group (*Aea*2) (Fig. S5). The phenyl rings make angles of 50.55° and 35.28° to the central pyridyl ring. The methyl unit of the 3,3'-methoxy groups are effectively in the plane of the phenyl rings, while those in the 2,2'-positions are perpendicular to it, with the O–CH_3 bond making a dihedral angle of 79.7° to the ring,

with these two methoxy groups on opposite sides of the molecule. This structure has been reported previously (Klein, A.; Butsch, K.; Neudörfl, J. *Inorg. Chim. Acta*, **2010**, *363*, 3282-3290).

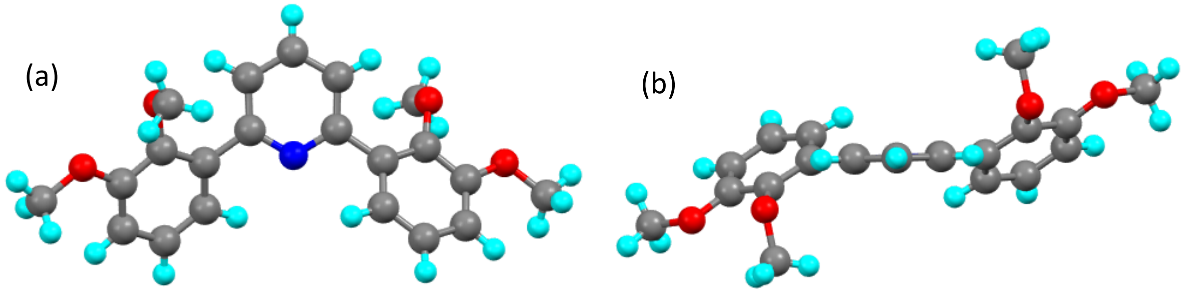

Figure S5 Molecular structure of **2,3-[L]** (a) viewed from above (b) viewed from the side to show the disposition of the 2,2'-methoxy groups.

2,4-[L]

2,4-[L] crystallises from both chloroform and ethanol in an orthorhombic space group ($P2_12_12_1$). The methoxy functional groups are co-planar with the phenyl rings, which make angles of 40.83° and 32.98° to the plane of the pyridyl ring (Figures S6a & S6b). Both phenyl rings are twisted in the same direction, with evidence of a steric barrier between the *m*-H on the pyridyl ring and the methoxy substituent in the 2,2′-positions on viewing in space-filling model (Fig. S6c), giving an achiral atropoisomer.

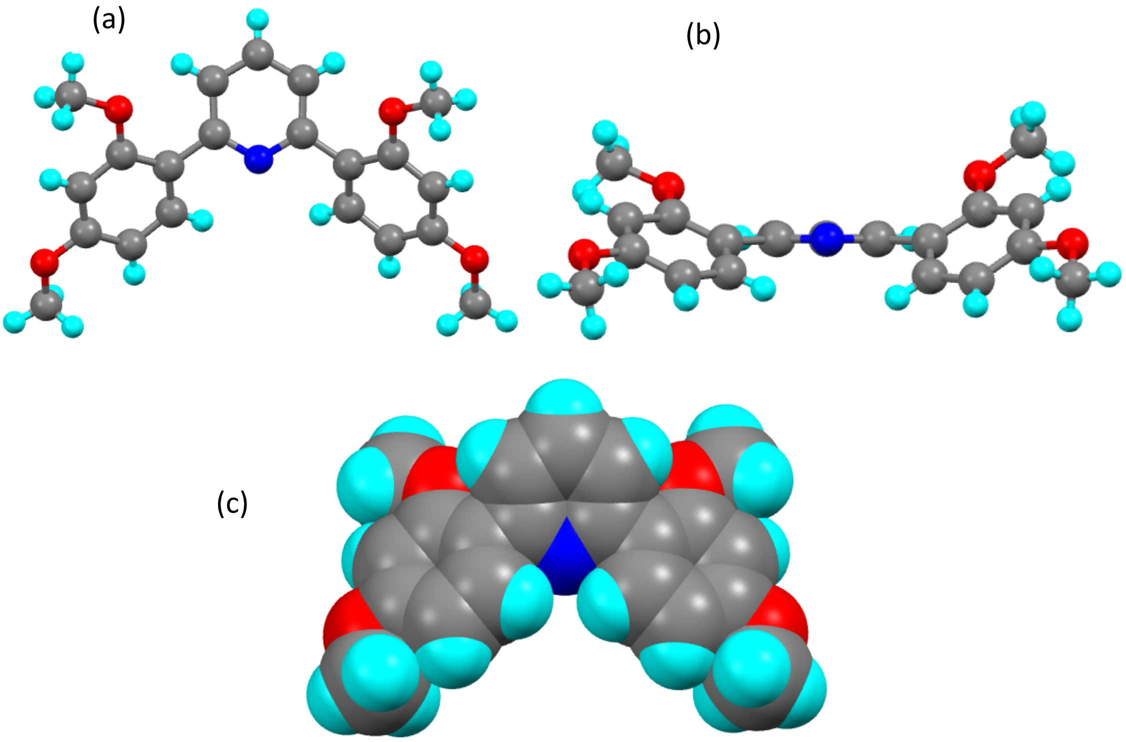

Figure S6 Structure of **2,4-[L]** (a) from above; (b) side-on view and (c) space-filling model.

2,5-[L]

2,5-[L] crystallises from chloroform and ethanol in orthorhombic space group (*Fdd*2). Twisting of the phenyl rings (each in opposite sense to the pyridine ring) gives an angle of 40.54° to the plane of the pyridyl ring (Figure S7).

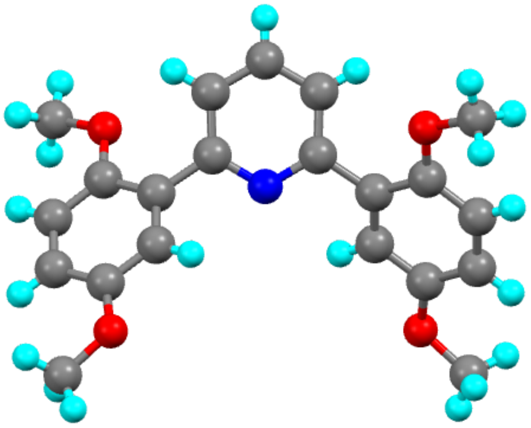

Figure S7 Molecular structure of**2,5-[L]**.

3-[L]

The structure has a large (*ca* 4400 Å^3) unit cell with Z = 12, respectively in the triclinic space group *P*-1. Half of the ligands have the two phenyl rings oriented in the same way with respect to the central pyridine ring, while half do not and the planes of the two phenyl rings subtend angles between *ca* 23 and 75°. In ten of the twelve ligands, the two methoxy groups point in opposite directions (Figure S8a), while in the other two the point in the same direction (Figure S8b).

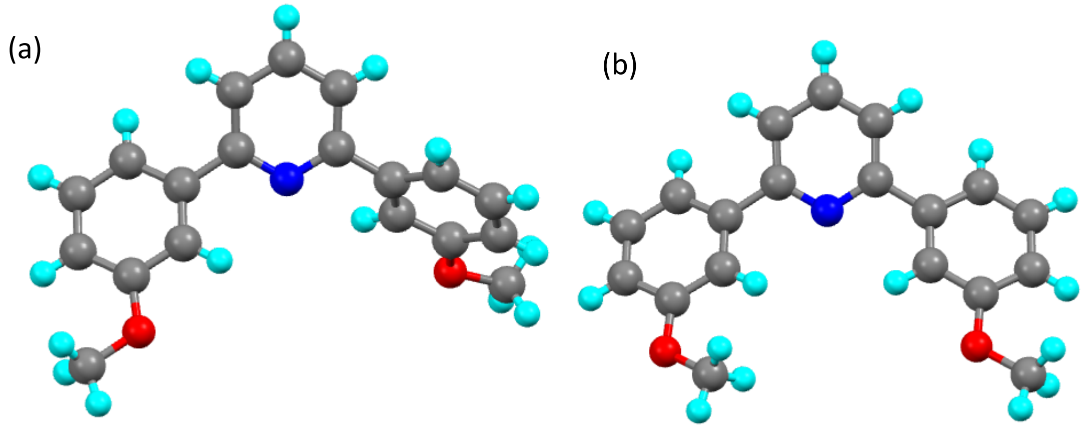

Figure S8 Molecular structure of **3-[L]**; (a) the molecule with the biggest twist angle between phenyl rings (b) the molecule with the *syn* methoxy group.

3,5-[L]

3,5-[L] crystallises from chloroform and ethanol in triclinic space group *P*-1. The phenyl rings are twisted out of the pyridyl ring plane subtending angles of 34.1(2)° and 23.3(2)° (Fig. S9).

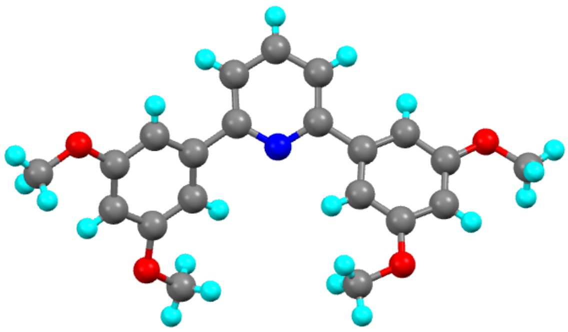

Figure S9 Molecular structure of **3,5-[L]**.

4-[L]

4-[L] crystallised from dichloromethane and ethanol in the tetragonal space group ($P4_12_12$) (Figure S10). The phenyl rings make angles of 21.19° and 21.57° to the central pyridyl ring. The structure of this ligand has been reported previously, (Isfahani, A. L.; Mohammadpoor-Baltork, I.; Mirkhani, V.; Khosropour, A. R.; Moghadam, M.; Tangestaninejad, S.; Kia, R. *Adv. Synth. Catal.*, **2013**, *355*, 957-972) where it was resolved in the tetragonal space group ($P4_32_12$), with slightly higher unit cell dimensions (7.9181(11) *vs* 7.83812(15) Å and 24.418(5) *vs* 24.0906(8) Å). However, in that case data collection was at a higher temperature (291(2) K *vs* 110.00(10) K), which might account for the discrepancy.

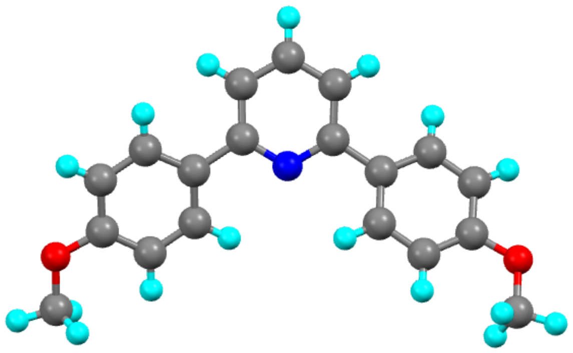

Figure S10 Molecular structure of **4-[L]**.

3,4-[L]

3,4-[L] crystallised from dichloromethane and ethanol in the monoclinic space group ($P2_1/n$) (Fig. S11). Rotation about the pyridine-phenyl bonds led to different relative disposition of the pairs of methoxy groups.

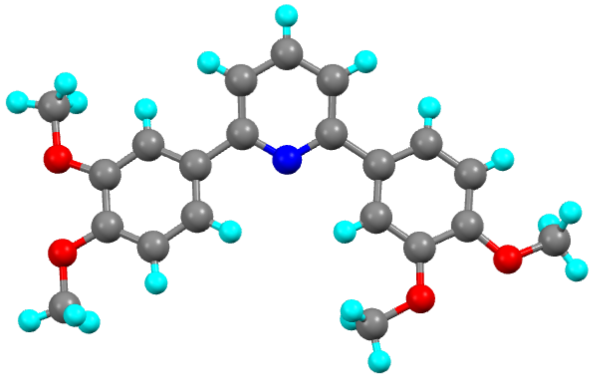

Figure S11 Molecular structure of **3,4-[L]**.

2,4-[LH]$^+$Cl$^-$

This salt crystallised during one preparation and is included here for completeness. The hydrochloride salt **2,4-[LH]$^+$Cl$^-$**, with two waters of crystallisation, crystallised from (evidently wet) ethanol/hexane in triclinic space group *P*-1 (Fig. S12). It can be assumed the chloride ion comes from the pyridinium chloride used in the deprotection reaction. The presence of two internal hydrogen bonds between the hydroxyl groups in the 2-position of the phenyl moiety and the N-H unit results in one phenyl ring being brought into the plane of the pyridyl unit, while the other is twisted at an angle of 29.98° to the plane of the other rings. Hydrogen bonding occurs between the chloride ion, the two water molecules and the hydroxyl groups.

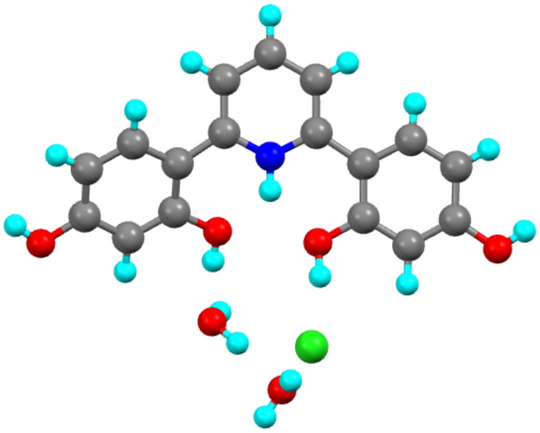

Figure S12 Molecular structure of the hydrochloride salt of 2,6-bis(2,4-dihydroxyphenyl)pyridine.

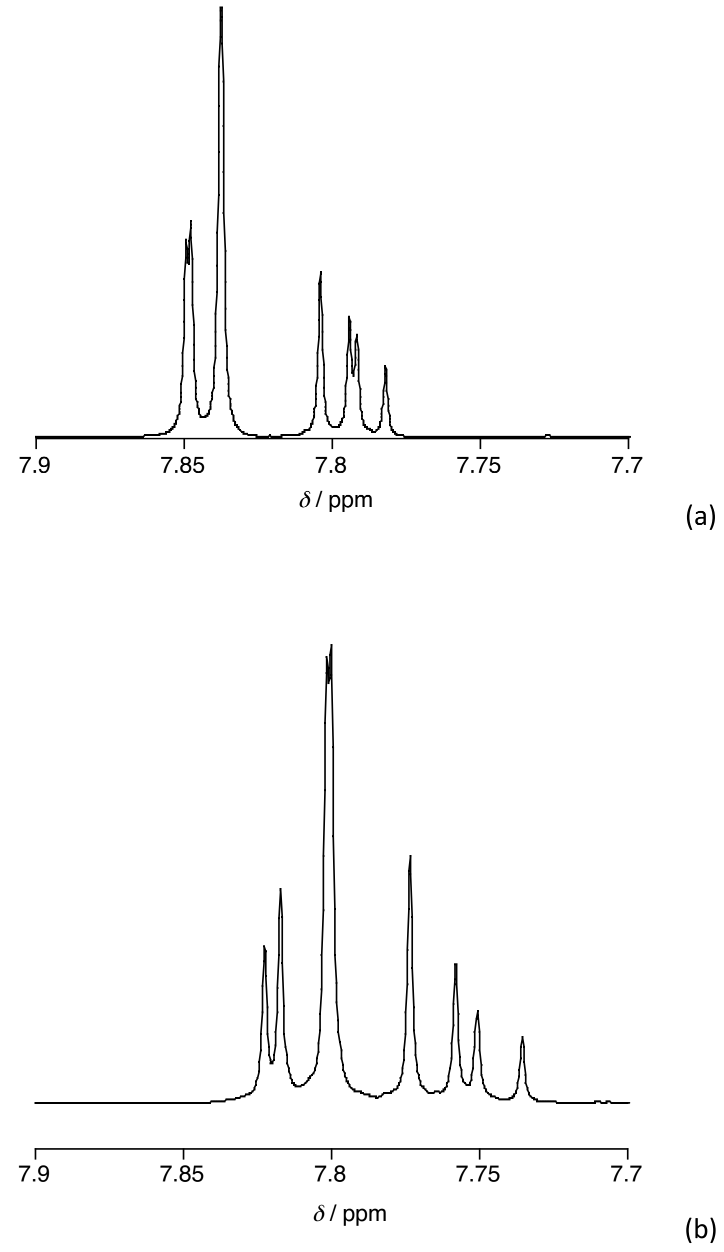

Figure S13 ^{1}H NMR spectrum of the pyridyl hydrogen atoms of **2,3-[L]-OMe** recorded in $CDCl_3$ at (a) 700 MHz and (b) 400 MHz (from McEllin, A. J.; Goult, C. A.; Whitwood, A. C.; Lynam, J. M.; Bruce, D. W. On the mercuration, palladation, transmetalation and direct auration of a C^N^C pincer ligand. *Dalton Trans.* **2022**, *52*, 872-876. DOI: 10.1039/d2dt04114f).

Temperature-Dependent ^{1}H NMR Spectroscopy of 2,3-[L]

To investigate the apparent atropoisomerism present in the compounds, variable temperature ^{1}H NMR experiments were carried out for **2,3-[L]-OMe** and **2,3-[L]**, in $CDCl_3$ from 253.0 K to 313.0 K at 500 MHz. For **2,3-[L]-OMe** spectra were obtained between 253 – 313 K (Figure S13 – S15), in

which range no change was observed in the peak shape nor in the nature of the splitting of the *m*-pyridyl hydrogens, indicating no exchange between the atropoisomers on the NMR timescale. Interestingly, the spectra show that the ring hydrogens of the phenyl rings shift remarkable little, while those of the pyridine ring move much more. On this basis, it is proposed that the change in chemical shift reflects increased motion (in effect librational frequency of the phenyl rings about the pyridine–carbon bond) and so an effective change in local environment.

However, while similar shifts are observed over the same temperature range in the spectra of **2,3-[L]** in both the aromatic region and also for the O–CH_2– hydrogens of the dodecyloxy chains, the appearance of the AB_2 spin system of the pyridyl hydrogens changes noticeably so that while remaining second-order, it starts to approach the simple doublet and triplet of a first-order AX_2 system. The origin of this evolution is not immediately apparent.

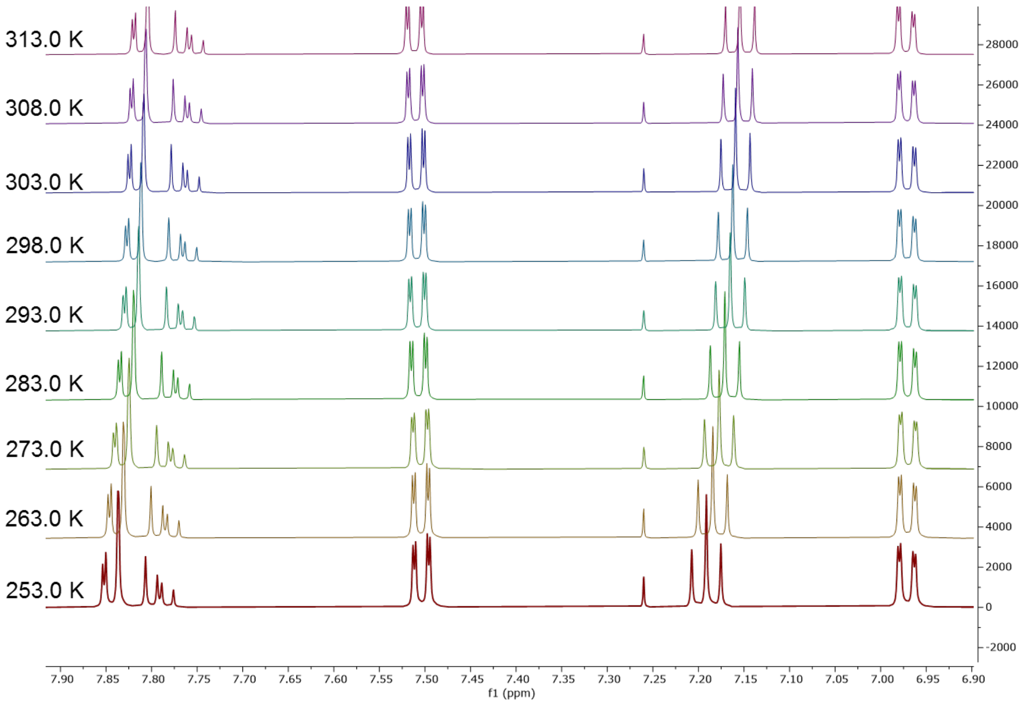

Figure S14 ^{1}H NMR spectra for **2,3-[L]-OMe** (500 MHz, $CDCl_3$) from 253 – 313 K.

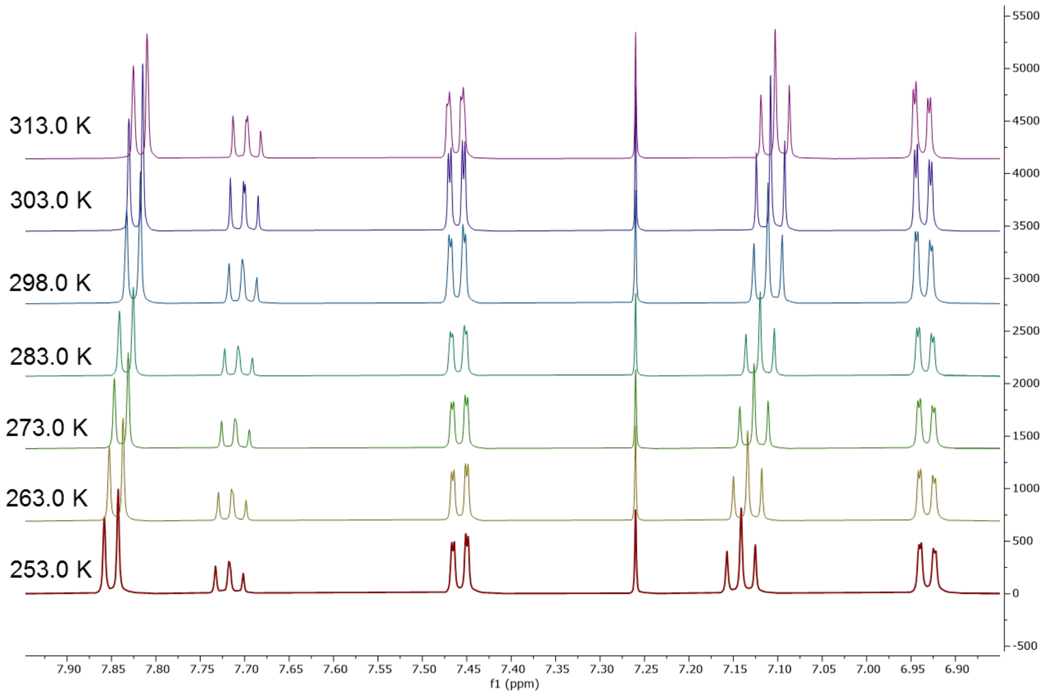

Figure S15 ^{1}H NMR spectra (aromatic region) for **2,3-[L]** (500 MHz, $CDCl_3$) from 253 – 313 K.

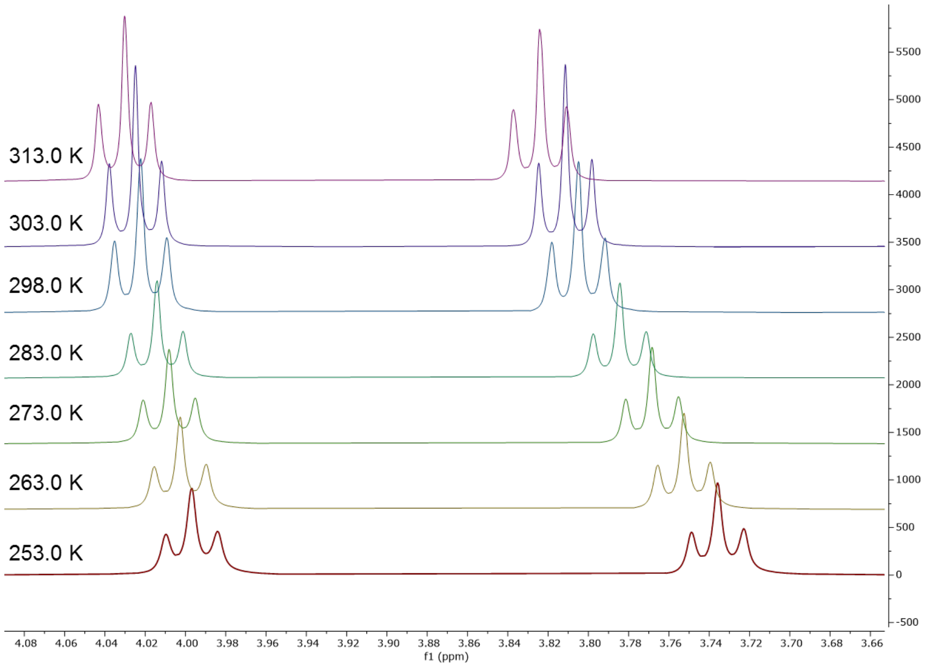

Figure S16 ^{1}H NMR spectra (alkoxy region) for **2,3-[L]** (500 MHz, $CDCl_3$) from 253 – 313 K.

Crystallographic Images Referred to in Main Manuscript

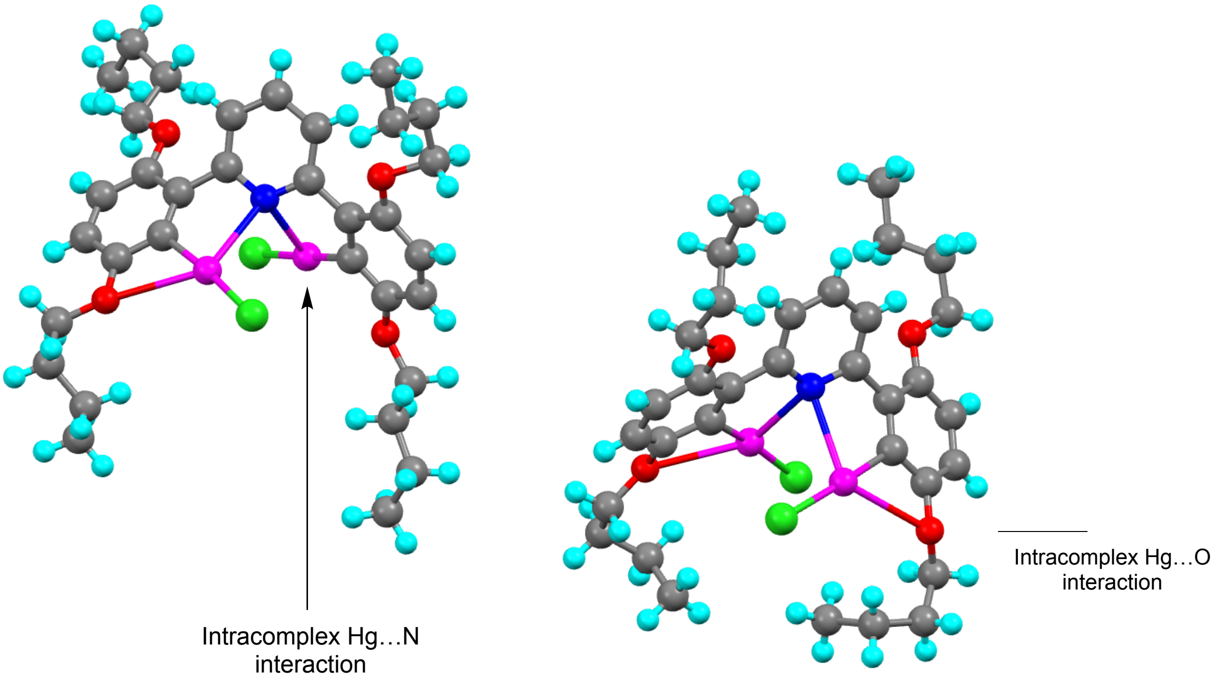

Figure S17 Structure of **2,5-[Hg$_2$]** showing different intra-complex interactions.

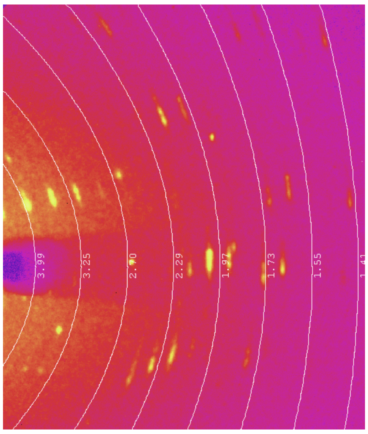

Figure S18 Example of XRD diffraction for **2,5-[Au]-Cl**, showing very broad, streaked reflections due to slippage of the crystal layers.

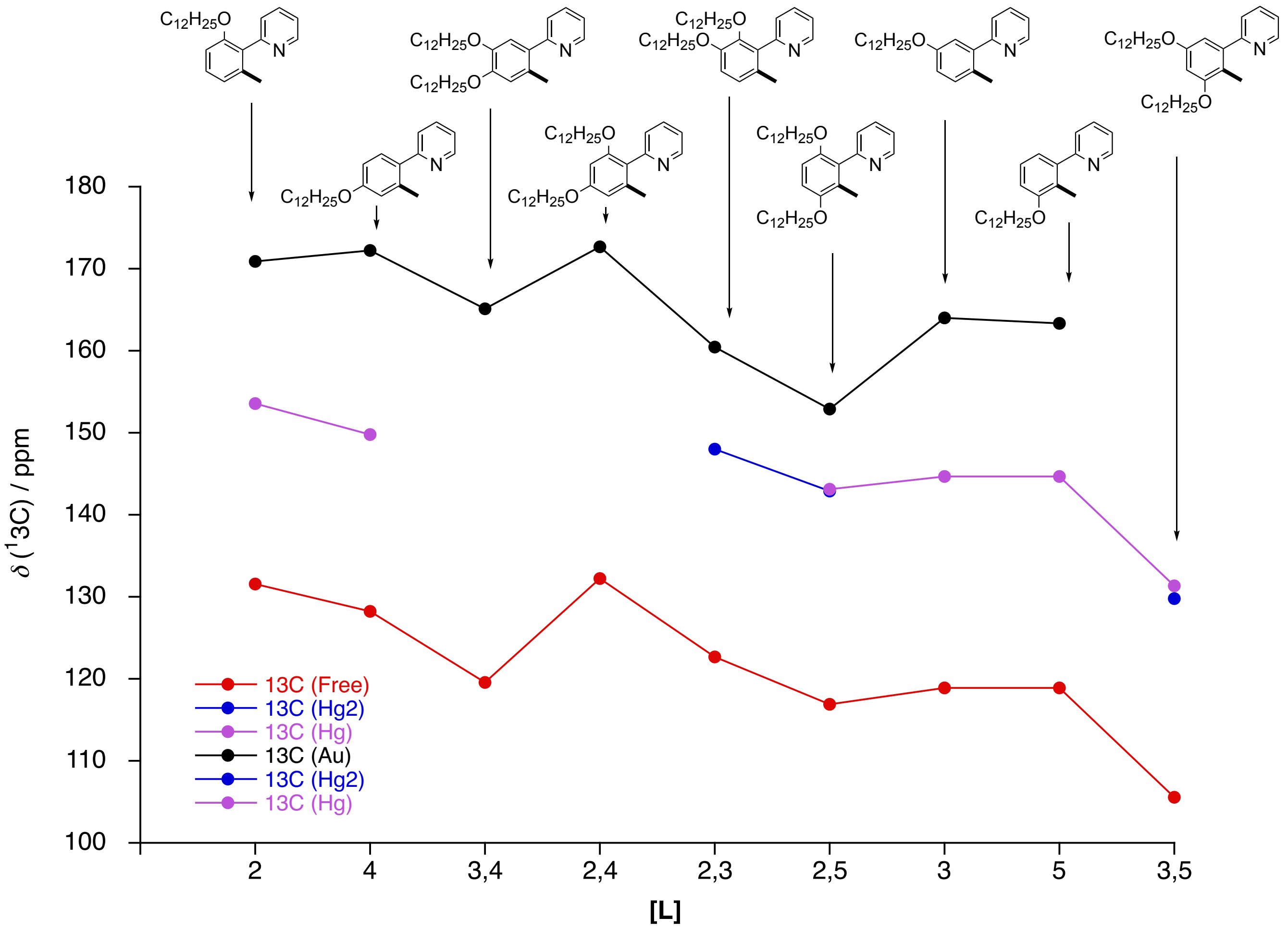

Figure S19 ^{13}C Chemical shift of the metalating/metalated carbon(s) for the free ligands, their mono- and di-mercury complexes and their gold complexes.

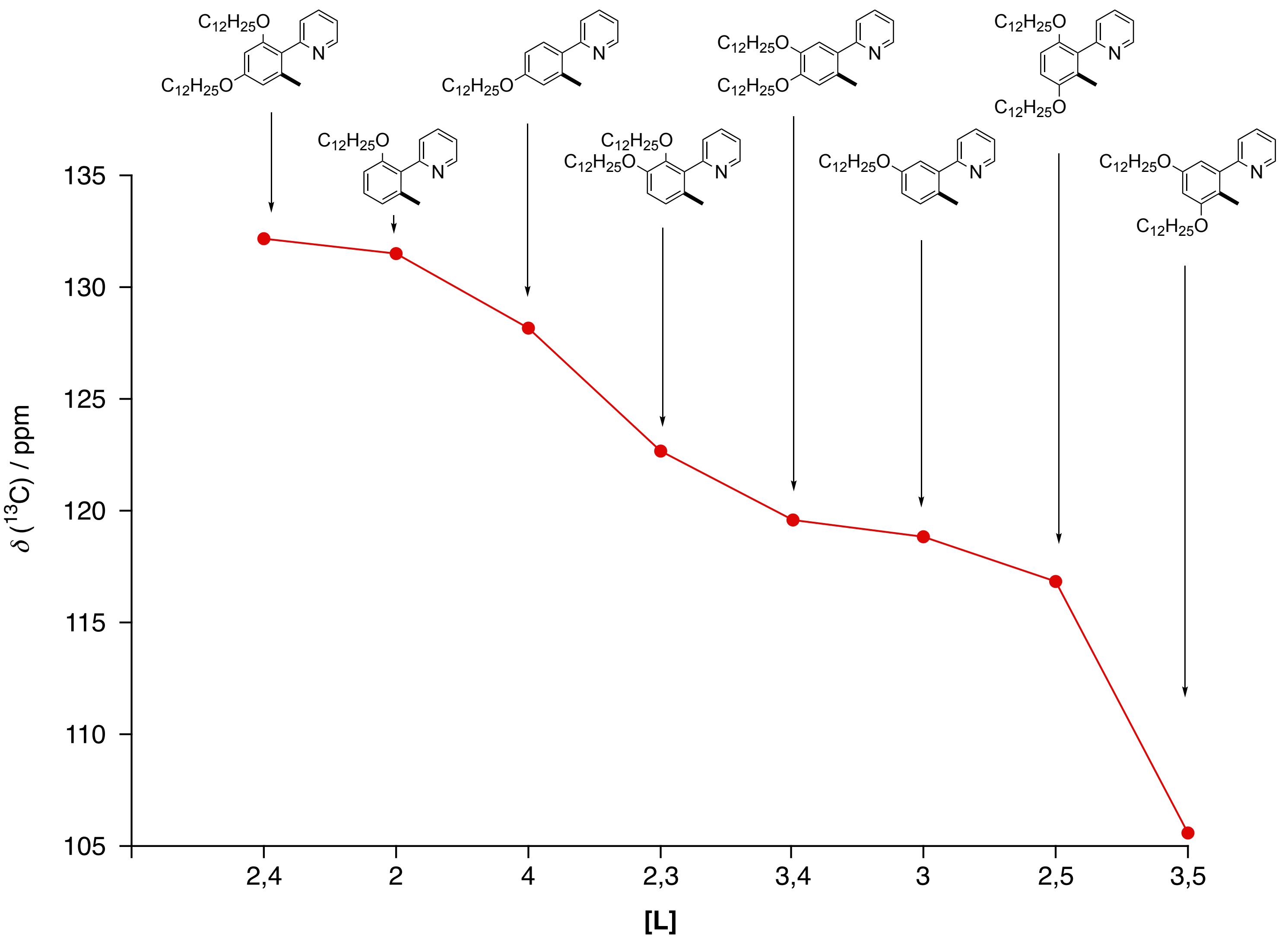

Figure S20 Plot of $^{13}C\{^{1}H\}$ NMR chemical shift of the different diphenylpyridine ligands by decreasing magnitude (thick bond represents the position of the metal.

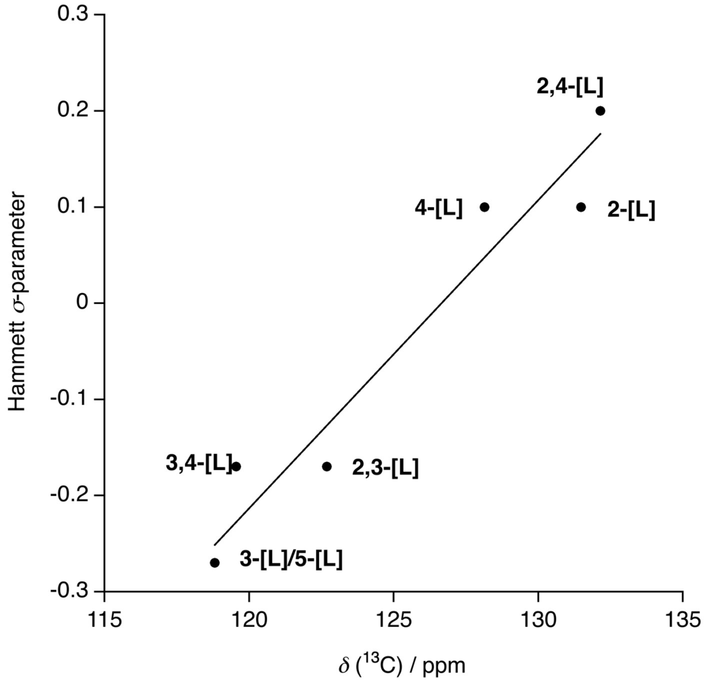

Figure S21 Plot of Hammet σ–parameter *vs* ^{13}C NMR chemical shift. σ–OMe(*m*) = 0.1; σ–OMe(*p*) = –0.27. Best-fit straight line is calculated by the program (Kaleidagraph) and gives $R \approx 0.97$. Hammett data from: Anslyn, E. V.; Dougherty, D. A., Modern Physical Organic Chemistry, University Science Books, **2006**.

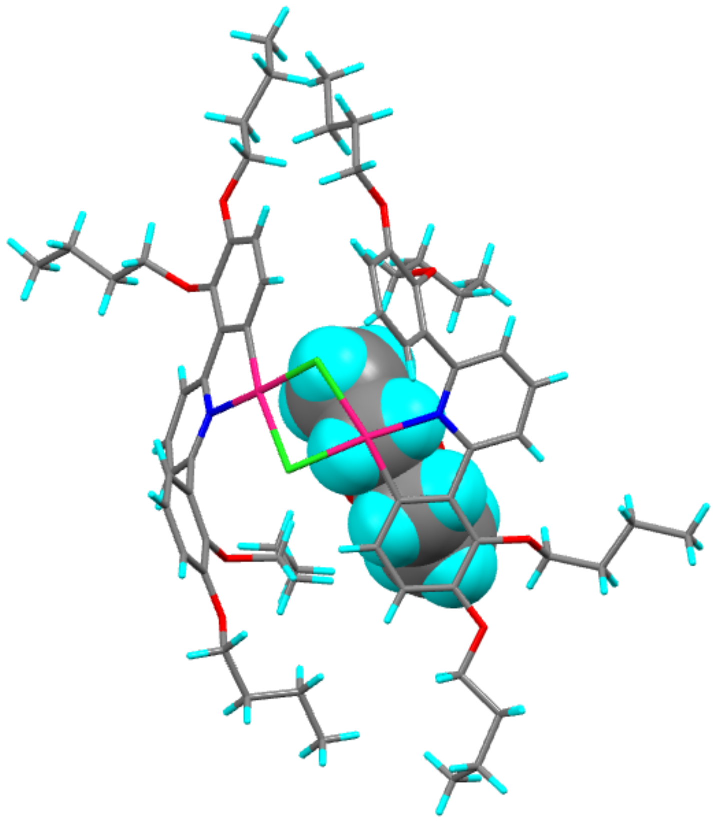

Figure S22 Illustration of the position of the diethyl ether solvate in the single crystal structure of **2,3-[Pd]**.
